# Transcriptome changes induced by abiotic stresses in *Artemisia annua*

**DOI:** 10.1038/s41598-018-21598-1

**Published:** 2018-02-21

**Authors:** Divya Vashisth, Ritesh Kumar, Shubhra Rastogi, Vikas Kumar Patel, Alok Kalra, Madan Mohan Gupta, Anil Kumar Gupta, Ajit Kumar Shasany

**Affiliations:** 1 Biotechnology Division, CSIR-Central Institute of Medicinal and Aromatic Plants, P.O. CIMAP, Lucknow, 226015 UP India; 2 Department of Biochemistry, University of Lucknow, Lucknow, 226007 UP India; 3 Microbial Technology Division, CSIR-Central Institute of Medicinal and Aromatic Plants, P.O. CIMAP, Lucknow, 226015 UP India; 4 Analytical Chemistry Division, CSIR-Central Institute of Medicinal and Aromatic Plants, P.O. CIMAP, Lucknow, 226015 UP India; 5 Genetics and Plant Breeding Division, CSIR-Central Institute of Medicinal and Aromatic Plants, P.O. CIMAP, Lucknow, 226015 UP India

## Abstract

*Artemisia annua* is known to be the source of artemisinin worldwide which is an antimalarial compound but is synthesised in very limited amount in the plant. Most research laid emphasis on the methods of enhancing artemisinin but our study has been planned in a way that it may simultaneously address two problems encountered by the plant. Firstly, to know the effect on the artemisinin content in the era of climate change because the secondary metabolites tend to increase under stress. Secondly, to identify some of the stress responsive genes that could help in stress tolerance of the plant under abiotic stress. Hence, the *A. annua* plants were subjected to four abiotic stresses (salt, cold, drought and water-logging) and it was observed that the artemisinin content increased in all the stress conditions except drought. Next, in order to identify the stress responsive genes, the transcriptome sequencing of the plants under stress was carried out resulting in 89,362 transcripts for control and 81,328, 76,337, 90,470 and 96,493 transcripts for salt, cold, drought, and water logging stresses. This investigation provides new insights for functional studies of genes involved in multiple abiotic stresses and potential candidate genes for multiple stress tolerance in *A. annua*.

## Introduction

The stresses such as drought, salinity, chilling, freezing, water-logging, variable light conditions, nutrient starvation and heat adversely affect the plant growth and productivity^1^. Plants undergo a series of physiological, morphological, molecular and biochemical changes on encountering stress. Plants have a remarkable ability to cope with the changing environmental conditions and try to adapt accordingly, depending upon the extent of stress^1^. Numerous genes and biological pathways are known to be involved in abiotic stress tolerance in many plants, and several others are thought to be involved^2,3^. Many genes and pathways are specific for a single type of stress and several others show co-regulation in different stresses^4,5^. It has also been reported that when a plant is made tolerant to one type of stress, it shows tolerance to some other stress also, indicating involvement of some common molecular mechanisms in different types of stresses^6–8^. Transcriptome sequencing and analysis is a reliable and effort -saving method to understand the global molecular response of the stressed plants^9^ which has been reported in many plants for a single type of stress^10,11^, however only few reports are available for combination and/or multiple stresses. In addition, there are limited studies on the impact of abiotic stresses on the medicinal plants. Hence, the present investigation reports about the response of *Artemisia annua* plant against four abiotic stresses (salt, cold, drought and water-logging/flooding).

*A. annua* L. belongs to Asteraceae family and is well known for its medicinal value to combat malaria^12,13^. Artemisinin is the anti-malarial compound found in the plant which also possesses other activities like anti-parasitic and anti-viral. The natural abundance of artemisinin in *A. annua* is very low (0.01–0.8%). Hence, several strategies have been used to fulfil the demand of high supply at a reduced rate^14,15^. Despite this, a limited success has been achieved for obtaining artemisinin *in vitro* and we are still dependent on *A. annua* plants. The artemisinin content of the plant depends on many parameters including the conditions like salinity stress, water stress, chilling stress etc.^16–18^ indicating that the stress related pathways and mechanisms somehow regulate the artemisinin biosynthesis pathway also affecting the yield of artemisinin. As a result, it becomes desirable to get a comprehension about the regulatory mechanisms involved in controlling artemisinin biosynthesis as well as strategies and mechanisms for increasing the overall plant yield, oil content and trichome density in severe and prolonged stress conditions. In non-model plants like- *A. annua*, identification of genes and pathways that are co-regulated in multiple stress conditions and help the plant to withstand the adverse conditions still need to be worked out and next generation transcriptome sequencing may prove a promising tool for such study. The hardy nature of this plant in tolerating various abiotic stress conditions makes it a strong candidate for carrying out next generation sequencing in order to explore the regulatory mechanism(s) of the artemisinin biosynthetic pathway as well as stress responsive pathways. Due to the limited transcriptomic data comparing multiple stress conditions in non-model plants, the present investigation describes the comparative analysis of *A. annua* leaf transcriptomes under four different stress conditions: cold, drought, salt and water-logging. In future, the data collected in the present study will prove to be a valuable asset for genomic studies of abiotic stresses in *Artemisia sp*.

## Results and Discussion

### Transcriptome sequencing and *de novo* assembly

Transcriptome sequences were generated from the cDNA(complementary DNA) libraries constructed using leaves of *A. annua* control sample and four abiotic stress samples - i.e. salt, cold, drought and water-logging stresses. Leaves from the whole plant were taken to carry out molecular and biochemical analysis. The sequencing of the prepared libraries was carried out on the Illumina NextSeq 500 platform with a sequencing depth range of 100× to 120×. The paired-end sequencing-by-synthesis generated a raw data of 32.9, 45.8, 64.8, 65.4 and 73.3 million reads from control, salt, cold, drought and water-logging stress sample libraries, respectively. Maximum read length was 151-bp for all the samples. After quality checking and processing of the raw reads data, 30.25, 43.17, 60.14, 61.45 and 69.45 million reads of control, salt, cold, drought and water-logging stress samples, respectively, were retained for further assembly (Supplementary file 1; Table S1). Filtered reads were assembled and transcripts were generated using Trinity at a hash length of 25. As a result of assembly total 89,362 transcripts for control sample while 81,328 (salt), 76,337 (cold), 90,470 (drought) and 96,493 (water-logging) transcripts were obtained in different stress samples. The average transcript lengths were of 1198.4, 1032.6, 1001.7, 1008.7 and 1014.3 bp with respective N50 values of 1,697, 1,491, 1,396, 1,424 and 1,438 for control, salt, cold, drought and water-logging stress samples (Supplementary file 1; Table S2). Variations obtained in the assembled transcript numbers from different samples (1 control and 4 stress samples) might be due to variable stress response of the plants, or as a result of technological noises at some stage of sequencing process^11^. The length of assembled transcripts ranged between 300 to >10,000 bases. Maximum number of transcripts was in the size range of 500–999 bp, which was followed by transcripts of 1,000–1,499 bp (Supplementary file 1; Fig. S1), in all the five assembled transcript data files, which is in coherence with *O. sanctum* assembled transcript data as reported by Rastogi *et al*.^19^.

### Assembly of transcripts showing differential expression

**Figure 1:**
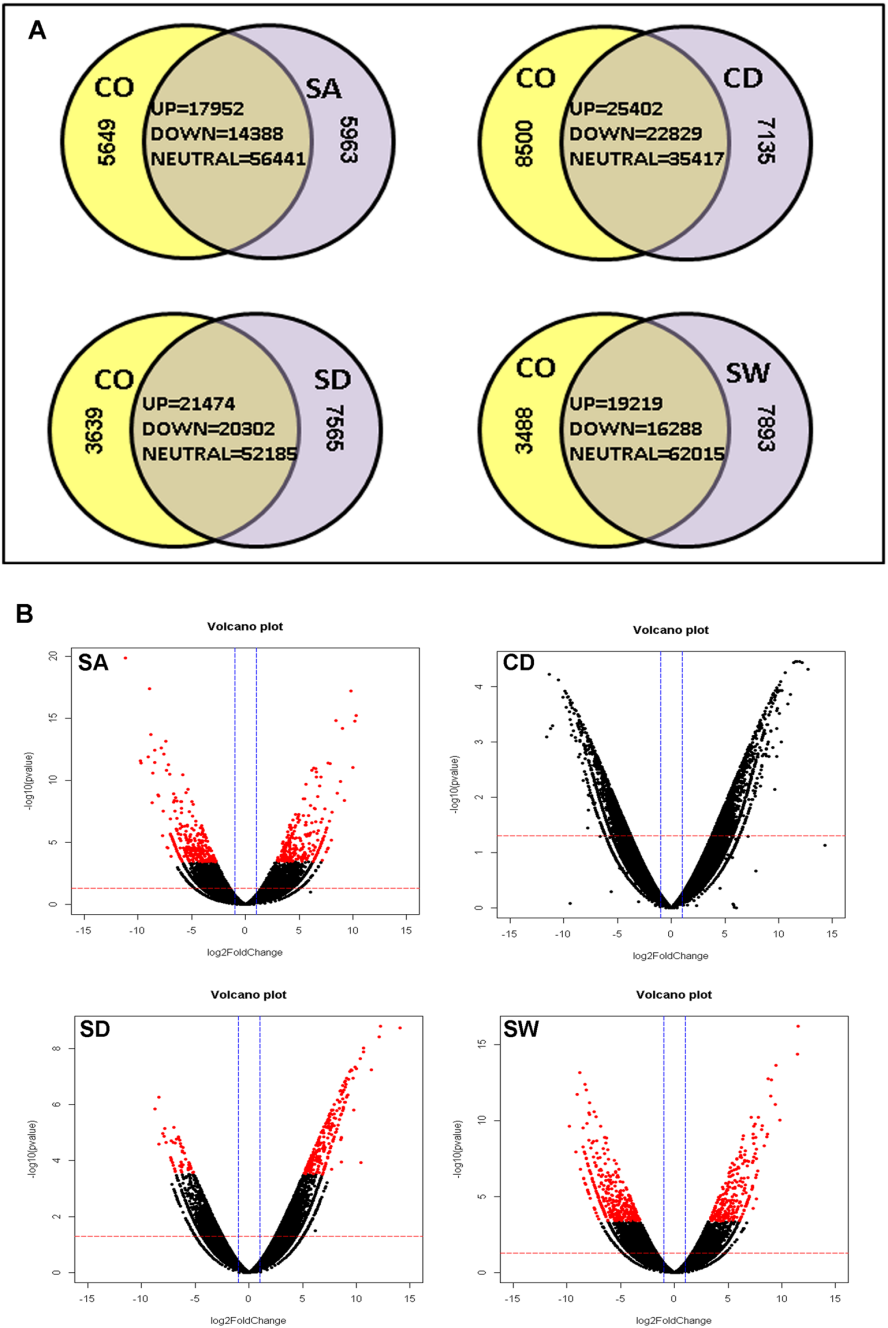
Distribution of DEGs. (**A**)Venn diagrams showing common and exclusive transcripts. The over-lapping regions display the common transcripts (up-regulated, down-regulated and neutral) between control and stress. (**B**) Volcano plots displaying differentially expressed transcripts. Each dot represents a DEG, dots above the red line display the significant DEGs (p < 0.05) and red dots indicate highly significant DEGs (q value < 0.05). Abbreviations used: CO (control sample), SA (salt), CD (cold), SD (drought), SW (water-logging), UP (up-regulated transcripts), DOWN (down-regulated transcripts), neutral (transcripts that did not exhibit a differential expression).

**Figure 2:**
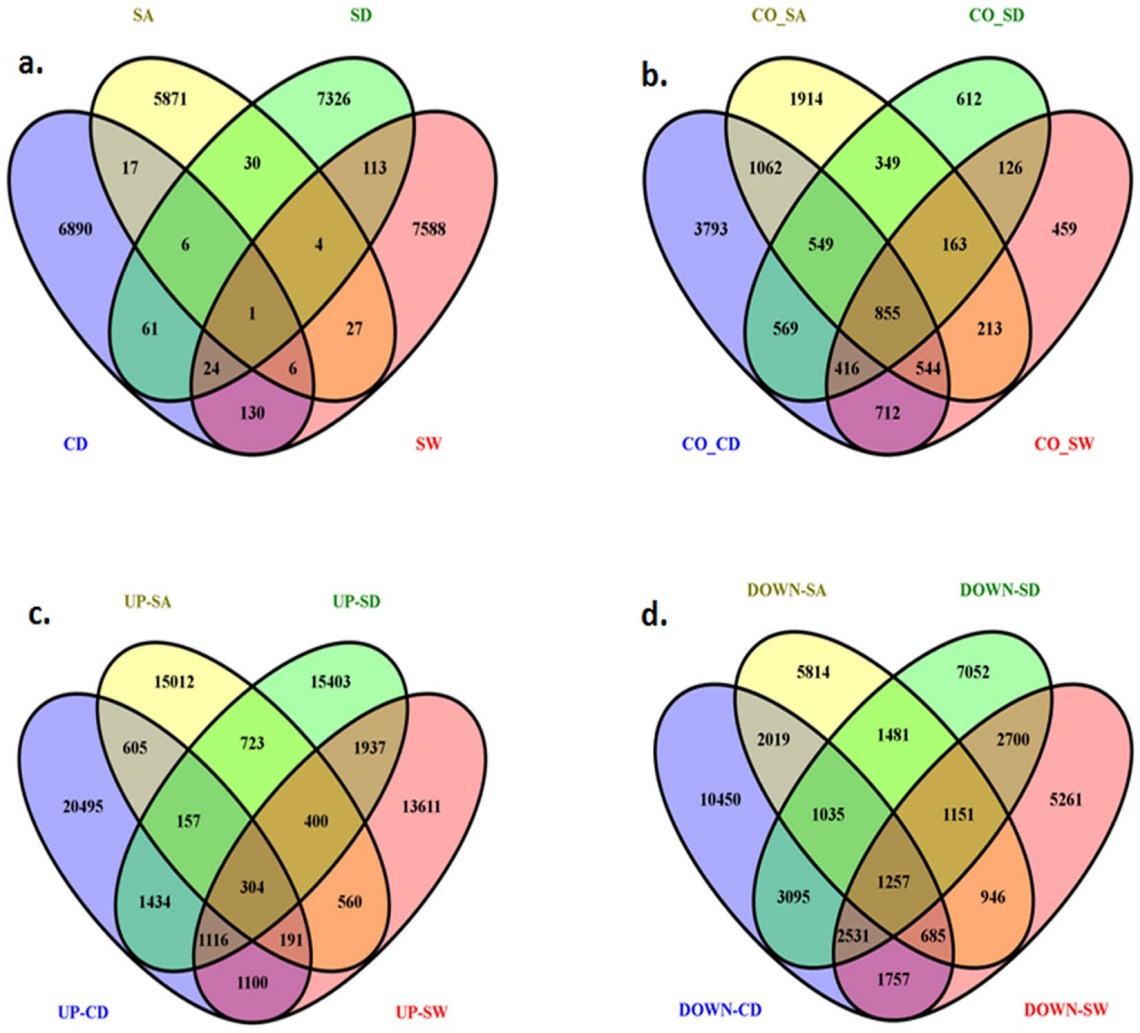
Venn diagrams showing common transcripts among sets of transcripts related to different stresses. Each circle represents a specific set of transcripts. Abbreviations used: (**a**) SA, transcripts exclusively expressed in salt stress; CD, transcripts exclusively expressed in cold stress; SD, transcripts exclusively expressed in drought stress; SW, transcripts exclusively expressed in water-logging. (**b**) CO_SA, transcripts exclusive to control when compared with salt; CO_CD, transcripts exclusive to control when compared with cold; CO_SD, transcripts exclusive to control when compared with drought; CO_SW, transcripts exclusive to control when compared with water-logging. (**c**) UP-SA, transcripts up-regulated in salt stress; UP-CD, transcripts up-regulated in cold stress; UP-SD, transcripts up-regulated in drought stress; UP-SW, transcripts up-regulated in water-logging stress. (**d**) DOWN-SA, transcripts down-regulated in salt stress; DOWN-CD, transcripts down-regulated in cold stress; DOWN-SD, transcripts down-regulated in drought stress; DOWN-SW, transcripts down-regulated in water-logging stress.

Transcripts from all stress samples were clustered with the transcripts from control sample using CD-HIT at 95% identity resulting in master control transcript data comprised of unigenes. A total of 1,01,995 transcripts were obtained for control and salt stress; 99,283 transcripts for control and cold stress; 1,05,165 for control and drought stress while 1,08,903 transcripts for control and water-logging stress. The distribution pattern of these transcripts is presented in Fig. 1. However, only 1,387 (salt), 1,320 (cold), 2,297 (drought) and 1,862 (water-logging) transcripts were significantly (p value < = 0.05) up-regulated and 1,829 (salt), 1,303 (cold), 1,647 (drought) and 2,298 (water-logging) transcripts showed significant (p value < = 0.05) down-regulation (Supplementary file 1; Fig. S2). For all stress DGE (differential gene expression) data, transcripts exclusive to stress, exclusive to control, transcripts up-regulated and down-regulated were analysed for over-lapping to identify common transcripts (Fig. 2).

### Functional annotation and GO classification

To assign putative functions to the assembled sequences, Blast x search was carried out for control and all 4 stress samples against viridiplantae protein sequences available in Uniprot database. In the case of control sample 41,618 transcripts (46.57%) while in case of salt stress 38,047 transcripts (46.78%), in cold stress 39,863 transcripts (52.21%), in drought stress 43,947 transcripts (48.57%) and water-logging stress sample, 45,091 transcripts (46.72%) were annotated (Supplementary file 1; Table S3). Each unigene was assigned one or more GO terms based on the GO term annotation of its corresponding homologue in the uniprot database when compared with the proteins of viridiplantae kingdom. GO annotations were retrieved from uniprot database for 19,601 (control sample), 25,838 (salt), 26,745 (cold), 29,478 (drought) and 30,551 (water-logging) transcripts which were classified into different functional groups under three main categories: molecular function (MF), biological process (BP) and cellular component (CC). Highest proportion of transcripts belonged to ‘unknown groups’ in all the three GO categories, followed by ‘binding activity’, ‘membranes’ and ‘other biological processes’ in all 5 samples (Supplementary file 1; Fig. S3). Reports on *O. sanctum* and *O. basilicum* transcriptomes also represented these three functional groups with maximum percentages of genes^19^. Percentages of genes in ‘unknown molecular functions’, ‘unknown cellular components’ and ‘unknown biological processes’ ranged between 42.35–61.03%, 72.65–78.59% and 67.13–75.78%, respectively, in the 5 sample libraries. The distribution pattern of the unigenes for all the five libraries under different GO terms exhibited similarity.

### KEGG analysis of *A. annua* transcriptomes

To get an overview of the active biological pathways in *A. annua*, mapping of the assembled and annotated sequences from all five samples to the reference canonical pathways in KEGG, using *Arabidopsis thaliana* (thale cress) and *Solanum lycopersicum* (tomato) as reference organisms, was performed. 14,372 (control), 10,585 (salt), 10,691 (cold), 11,012 (drought) and 11,112 (water-logging) transcripts were functionally assigned to 125 KEGG pathways. (Supplementary file 1; Fig. S4). 638 (control), 502 (salt), 553 (cold), 624 (drought) and 638 (water-logging) transcripts exhibited involvement in biosynthesis of various secondary metabolites. Among the secondary metabolism category, ‘Phenylpropanoid biosynthesis’ emerged as the largest group followed by ‘terpenoid backbone biosynthesis’ in all 5 sample libraries (Supplementary file 1; Figs S5 and S6). Transcripts showing a significant (p value < = 0.05) up regulation and down-regulation in all 4 stresses were also mapped to terms in KEGG database (Supplementary file 1; Fig. S7).

### GO functional enrichment and KEGG pathway enrichment analysis of DEGs (differentially expressed genes)

All the DEGs in all four stresses were analysed for identification of enriched GO terms and enriched KEGG pathways. Number of significantly (FDR ≤ 0.05) enriched GO terms for DEGs showing up-regulation were: 25 (cold), 49 (salt), 41 (drought) and 39 (water-logging). In contrast, 22 (cold), 36 (salt), 19 (drought) and 33 (water-logging) GO terms were significantly (FDR ≤ 0.05) enriched for DEGs showing down-regulation (Supplementary file 2). 16 and 12 significantly enriched GO terms were common for up-regulated DEGs and down-regulated DEGs in all stresses, respectively. In the biological process category, ‘carbohydrate metabolic process’ and ‘cellular processes’ were significantly enriched for both up and down-regulated transcripts, suggesting that that genes corresponding to these processes play important role in abiotic stress responses. In the molecular function category, significantly enriched GO terms common for both up and down-regulated DEGs were as: ‘transferase activity’, ‘catalytic activity’, ‘nucleotide binding’, ‘transferase activity, transferring phosphorus-containing groups’, ‘binding’, ‘kinase activity’ and ‘chromatin binding’. This suggests that expression of the transcripts related to gene expression regulation and signal transduction are highly modulated during different abiotic stresses. ‘Membrane’ was identified as the most enriched common cellular component category term for both up and down- regulated DEGs. It has been reported that during adverse environmental conditions, membrane transport and perception systems play important role to maintain cellular homeostasis in plants and expression of genes for membrane transporters, channel proteins, receptor-like protein kinases are up-regulated^20^.

The two most enriched significant (FDR ≤ 0.05) KEGG pathways for up-regulated DEGs were ‘metabolic pathways’ and ‘photosynthesis’ in cold; ‘oxidative phosphorylation’ and ‘metabolic pathways’ in salt stress; ‘biosynthesis of secondary metabolites’ and ‘fatty acid metabolism’ in drought stress and ‘metabolic pathways’ and ‘biosynthesis of secondary metabolites’ in water-logging stress. For down-regulated transcripts, most enriched pathway was ‘N-Glycan biosynthesis’ in cold and water-logging stress; ‘protein processing in endoplasmic reticulum’ in salt stress and monoterpenoid biosynthesis in drought stress (Supplementary file 3). Enriched GO terms and enriched KEGG pathways analysis could provide a probable insight into the molecular mechanism of abiotic stress response.

### Differentially regulated stress-responsive genes during stress

**Figure 3:**
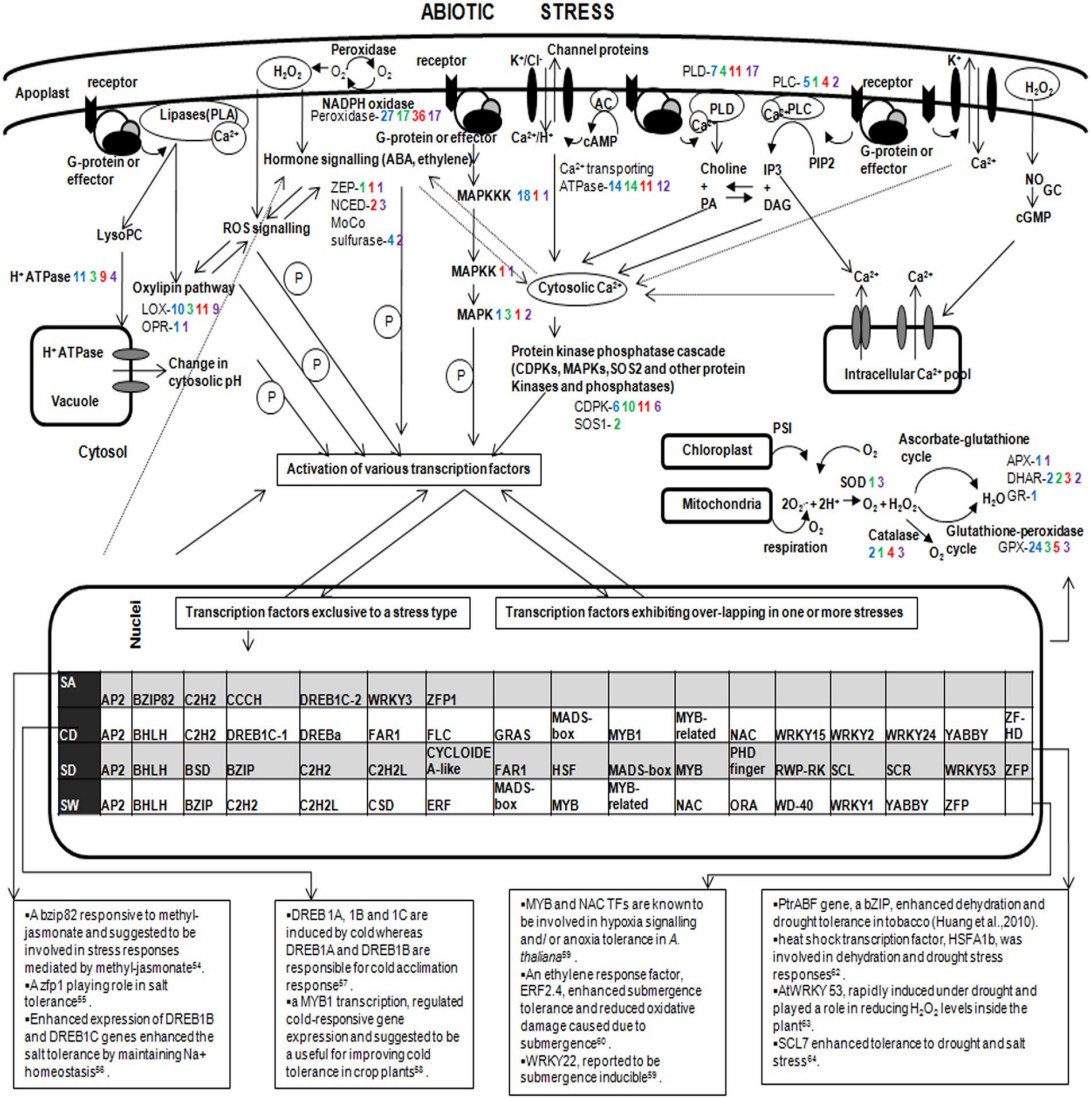
Schematic representation of Signal transduction network during abiotic stresses along with transcription factors specific to each stress. Numbers represent the transcripts found up-regulated or exclusively expressed during stress condition. Blue color indicates cold stress; green, salt stress; red, drought stress; purple, water-logging stress. Abbreviations used: ascorbate peroxidase (APX); dihydroascorbate reductase (DHAR); glutathione reductase (GR); superoxide dismutase (SOD); glutathione peroxidase (GPX); lipoxygenase (LOX);12-Oxophytodienoic acid reductase (OPR); 9-*cis* epoxycarotenoid dioxygenase (NCED); zeaxanthin epoxidase (ZEP); Phospholipase A (PLA); Phospholipase C (PLC); phosphatidylcholine (PC); Phospholipase D (PLD); phosphatidylinositol 4,5-diphosphate (PIP2); lysophosphatidylcholine (lyso PC); phosphatidic acid (PA); inositol 1,4,5- trisphosphate (IP3); diacylglycerol (DAG); mitogen-activated protein kinase kinase kinase (MAPKKK); mitogen-activated protein kinase kinase (MAPKK) and mitogen-activated protein kinase (MAPK)^8,53–64^.

Various stress responsive genes were differentially regulated during different abiotic stresses conferring that the plants were in stress. Large numbers of kinases, peroxidases, genes involved in ABA biosynthesis etc were up-regulated in all stresses. LEA (Late embryogenesis abundant protein) and LEA-like proteins, various desaturases, glyoxalase I family protein, genes involved in oxylipin and polyamine biosynthesis, delta 1-pyrroline-5-carboxylate synthetase, dehydrin etc., which are well known cold stress responsive genes, exhibited up-regulation during cold stress^1,8^. During salt stress, many salt stress responsive genes like SOS1 (Salt overly sensitive 1), various H^+^-ATPases, serine/threonine protein kinases, glutathione-S-transferase, glyoxalase I etc were highly up-regulated as reported in earlier studies^1,8^. Drought stress induced the expression of many genes that are indicative of water stress like Delta 1-pyrroline-5-carboxylate synthetase, aquaporins, glyceraldehyde-3-phosphate dehydrogenase, LEA proteins, dehydrin, heat shock protein, glyoxalase I, glutathione-S-transferase, PR proteins, calcium-dependent protein-kinases, genes involved in ethylene and oxylipin biosynthesis etc^20^. Plants exposed to water-logging stress exhibited enhanced expression of genes involved in ethylene biosynthesis, alcohol dehydrogenases, catalase etc, as demonstrated by the earlier reports on flood stress^21^. Based on known stress genes showing up-regulation or exclusive expression during stress condition, Fig. 3 has been designed representing an overview of stress perception, stress-signalling and stress response.

### Transcription factors

60 different TF family members were identified in *A. annua* leaf transcriptome libraries (Supplementary file 1; Table S4). These sequence specific DNA-binding proteins play an indispensable role in stress signal transduction pathways. Various TF families such as AP2/EREBP, bZIP, MYC, AREB/ABF, MYB, WRKY, HB DREB1/CBF and NAC reportedly regulate stress response in plants^22^.

As the stress is prolonged, many functional genes get up-regulated whereas several regulatory genes do not show differential expression anymore^20^. Samples used for preparation of transcriptome library were exposed to prolonged stresses. This seems to be the reason that many well known TFs did not show significant differential expression (p value ≤ 0.05). Transcripts belonging to 29 TF families exhibited significant up-regulation in one or more stresses (Supplementary file 1; Table S5). NAC and MYB /MYB related TF family members were showing significant up-regulation in all 4 types of stress and these might prove to be potential candidates for studying multiple stress adaptation and stress tolerance. A large number of NAC TFs showing differential expression under stress have been identified in whole-genome expression profiling and transcriptome studies in many plants^23,24^. Also, many transgenic studies performed in different plant species, like *A. thaliana*, *O. sativa*, *N. tabacum*, *G. max* and *T. aestivum*, have shown that manipulation of specific NAC TF can confer stress tolerance to the plants^25^. Many R2R3-type MYB TFs and a few MYB related TFs have been reportedly involved in diverse abiotic stresses^3,26,27^.

Transcription factors exclusively expressed during a stress condition but absent in control sample were also investigated for overlaps in order to identify transcription factors specific to a single type of stress. Transcription factors thus identified are shown in Fig. 3; quite a few of them have been amplified for validation purpose (Supplementary file 1; Fig. S8). Some of the TFs identified this way showed similarity with known stress related TFs, thus, further validating the stress condition of the plants. Besides this, some new TFs were also identified which might provide important leads for studying stress-signalling and stress-tolerance mechanisms in individual stress conditions.

### Genes co-expressed exclusively in stress samples

5,963 (salt), 7,135 (cold), 7,565 (drought) and 7,893 (water-logging) transcripts got exclusively expressed during stress and were not detected in the control sample. These exclusive transcripts were investigated for common ones among the four stresses. 41 transcripts were identified to be expressing in three or more stress samples. Interestingly, only 1 unigene (master control_78545) was found that was getting expressed in all the four stress samples (Supplementary file 1; Table S6). The blast x analysis of this unique sequence against the non-redundant protein sequence (nr) database at NCBI revealed its resemblance with a hypothetical protein B456_003G053000 of *Gossypium raimondii*, to which it was having 56% identity with e value of 0.033 but the query cover percentage was only 10%. The hypothetical protein B456_003G053000 is reportedly predicted peptidyl tRNA hydrolase (PTH). PTH activity is responsible for releasing tRNA from the premature translation termination product peptidyl-tRNA, thus rendering the tRNA and peptide reusable for the protein synthesis process. There is limited information available for PTH in eukaryotic system though it has been extensively studied in bacterial system^28^. Stressful conditions may lead to increase in premature translation termination and accumulation of peptidyl-tRNA in the cytosol, which may interrupt the normal cellular processes and probably the plant cells synthesized this unique transcript to overcome this situation by breaking the ester bond between tRNA and peptide and setting free the tRNA molecules^28^. But, since the query cover for the blast result was too low, therefore, this transcript may be involved in overcoming stress through this proposed mechanism or by some other mechanism. Other transcripts showing expression in any 3 stresses included proteins with ribonuclease III activity, DNA methyltransferase activity, spermidine synthase, putative helicases etc. that are known to be stress responsive genes^29–31^. Since the 41 transcripts showing over-lapping in three or more stresses, as discussed above, could not be detected in the control sample, therefore, either the expression of these transcripts was completely absent or their expression was extremely low or negligible in favourable environmental conditions and hence they can be considered as ‘stress-specific genes’. This study suggests that further characterization and functional analysis of these transcripts may explore some novel ‘stress marker’ genes as well as genes having potential to alter stress-tolerance in the plants.

### Transcripts unexpressed in all the stresses

**Figure 4:**
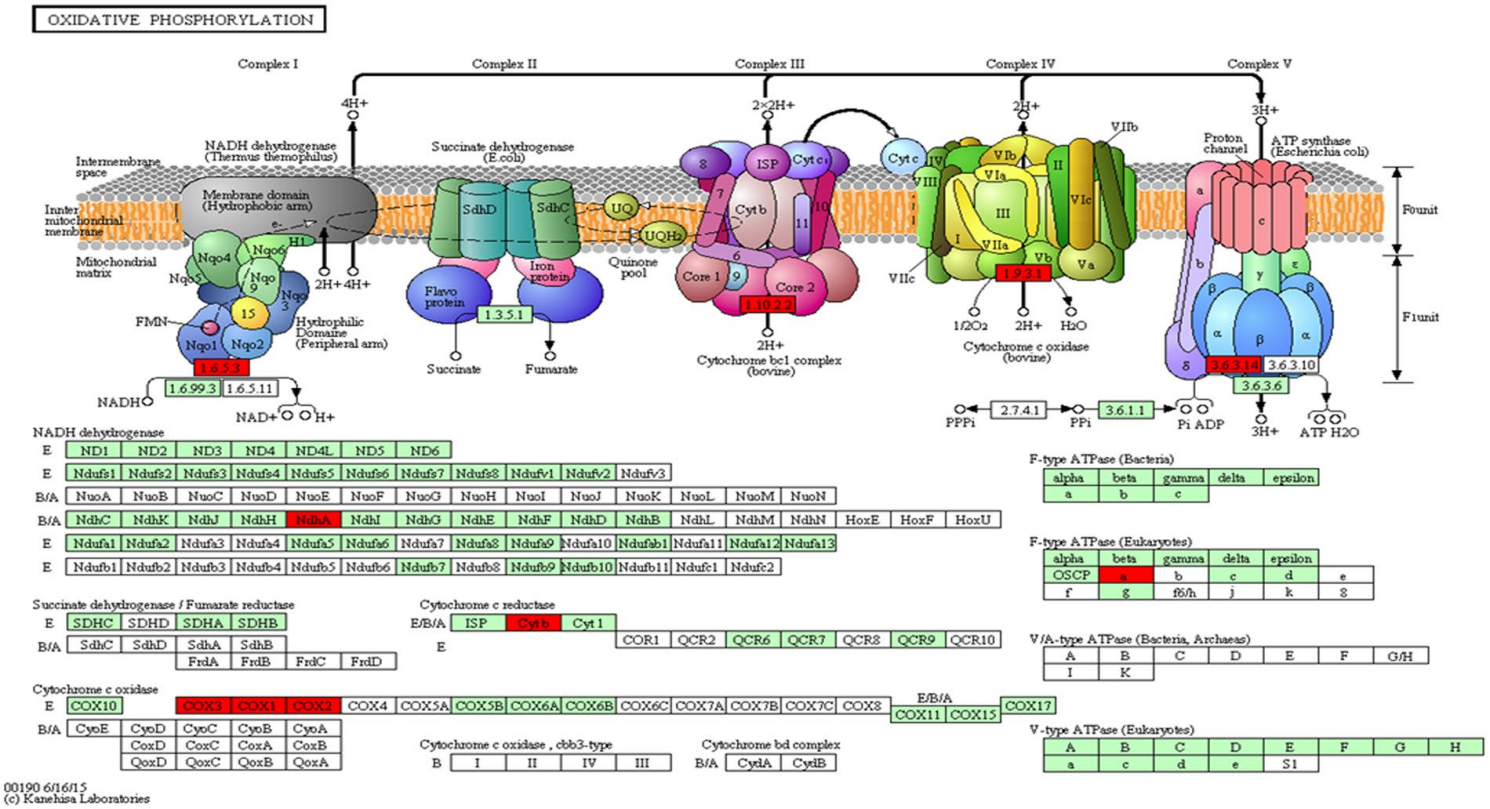
KEGG pathway diagram for oxidative phosphorylation. Oxidative phosphorylation emerged as the most significantly enriched pathway when transcripts showing expression only in control sample were subjected to KOBAS. The upper part of the figure displays the respiratory chain complexes and the lower portion represents their subunits as rectangles (red ones are the transcripts unique to control sample when compared to stress the bottom part, rectangles indicate the subunits of each respiratory chain complex. Red colored boxes represent the genes that were present exclusively in control sample whereas green boxes represent the genes present in reference organism^65–68^.

When transcriptome of each stress sample was compared with the control sample, set of transcripts showing expression only in the control sample and absent during stress was identified for each stress. 5,649, 8,500, 3,639 and 3,488 transcripts exclusively belonged to the control sample when the control transcriptome was compared to the transcriptome of salt, cold, drought and water-logging sample, respectively. The four sets of transcripts identified in this way were further analysed to get the common transcripts between these four sets and a set of 855 transcripts was identified. The transcripts thus identified were subjected to KEGG pathway enrichment analysis using KOBAS (Supplementary file 4). Only 6 terms exhibited significant (p-value ≤ 0.05) enrichment whereas only one term (oxidative phosphorylation) was highly significantly enriched (corrected p-value ≤ 0.001). The transcripts identified for oxidative phosphorylation term were shown in Fig. 4. In plant mitochondria, the oxidation and phosphorylation reactions leading to ATP generation are not always coupled. Under certain circumstances such as abiotic stresses, the link between respiratory electron transport and ADP phosphorylation is impaired or disrupted^32^. Abiotic stresses lead to ROS generation by higher plant mitochondria. Uncoupling of electron transport chain and oxidative phosphorylation acts as a mechanism to regulate this ROS generation. Proteins such as alternative oxidase (AOX), uncoupling proteins etc, help the mitochondria in regulating ROS levels. When energy dissipating alternative oxidase pathway becomes active, which dampens the generation of ROS in mitochondria by preventing over reduction of electron transport chain components, electrons flow from ubiquinone to AOX and two sites of proton pumping (at complexes III and IV) are bypassed^32^. Since the functional role of complex III (cytochrome bc1 complex) and complex IV (cytochrome c oxidase) is not of primary importance in such conditions. Also, when alternative pathways become active in the mitochondria, energy conservation in the form of ATP is hindered because of the absence of some energy conservation site in this pathway and hence, the role of ATP synthase (complex V) also becomes limited. Thus, oxidative phosphorylation and electron transport chain seems to be affected in all 4 abiotic stress conditions. Zsigmond *et al*.^33^, demonstrated that there exists a link between regulation of oxidative respiration and environmental adaptation in *Arabidopsis*^33^. The transcripts identified in the present study could provide putative genes that can be targeted for metabolic engineering to strengthen the plant during stressful conditions or to sustain the normal growth and development of the plant even in unfavourable conditions.

### Differentially expressed genes showing co-regulation

A total of 1,561 transcripts were commonly regulated in all 4 stresses. 304 transcripts were commonly up-regulated, amongst these 4 transcripts annotated as uncharacterized proteins showed significant up-regulation in all the four stress samples (Supplementary file 1; Table S8). Out of 304 transcripts, only 91 could be assigned a putative function. These included Photosystem II protein D1, NADH-dependent glutamate synthase 1, recombination protein DMC1, serine-threonine protein kinases, linoleoyl desaturase, 1-aminocyclopropapne-1-carboxylic acid oxidase, ATP synthase subunit, Glycerol-3-phosphate dehydrogenase, MUTS, ETR2 etc. The photosynthesis process is highly sensitive to environmental stresses. Various stresses lead to an increase in the level of ROS in chloroplast which damage the D1 protein of PSII and also hinder its *de novo* synthesis, thus interfering with PSII repair^34^. Thus, the probable reason of the up-regulation of D1 protein transcripts in all the stresses in the present study was to overcome PSII inhibition during prolonged stress. Under stress conditions, proteolytic activity increases resulting in increased intracellular hyperammonia and toxicity. These excess ammonium ions produced during stress need to be eliminated for plant survival. Glutamate synthase/glutamine synthetase incorporate toxic free ammonium ions into glutamate and glutamine respectively, thus up-regulation of these genes might be a probable mechanism of stress tolerance^35^. Also, glutamate is the precursor molecule for proline (an osmo-protectant), arginine (one of the precursors of putrescine) and GABA. There are several reports showing that high levels of GABA, proline and putrescine accumulate in plant tissues when exposed to various stresses and play an important role in stress-regulation and stress-tolerance^36–38^. An important biochemical mechanism for regulating signalling pathways leading to stress-specific response or stress tolerance is reversible protein phosphorylation. Serine-threonine protein kinases are known to be involved in regulation of signalling cascades and some of these when over-expressed enhanced stress-tolerance of the plant^39^.

1,257 transcripts were commonly down-regulated in different stresses, out of which only 202 transcripts could be assigned a putative function and only 42 transcripts exhibited significant downregulation in all 4 stresses (Supplementary file 1; Table S9). The DEGs that were commonly down-regulated had a wide range of functions. Heat shock proteins (HSPs), N-acetyl glucosaminyl transferase I, NBS-LRR proteins and sucrose transport proteins were largely represented. Most HSPs are generally induced by abiotic stresses but they have been reportedly down-regulated in *Ammopipanthus mongolicus* under drought and cold stress^20^ and HSP60 was found to be commonly down-regulated in cold, salt and mannitol stress in *Arabidopsis*^1^. Downregulation of some of the NBS-LRR proteins (disease resistance proteins with nucleotide-binding site and leucine-rich repeats) in all stresses suggest that probably *A. annua* plants become susceptible to some specific pathogen when exposed to prolonged abiotic stress. Many differentially regulated NBS-LRR proteins have been reported in *Arabidopsis* plants exposed to a combination of drought and heat stress^40^.

**Figure 5:**
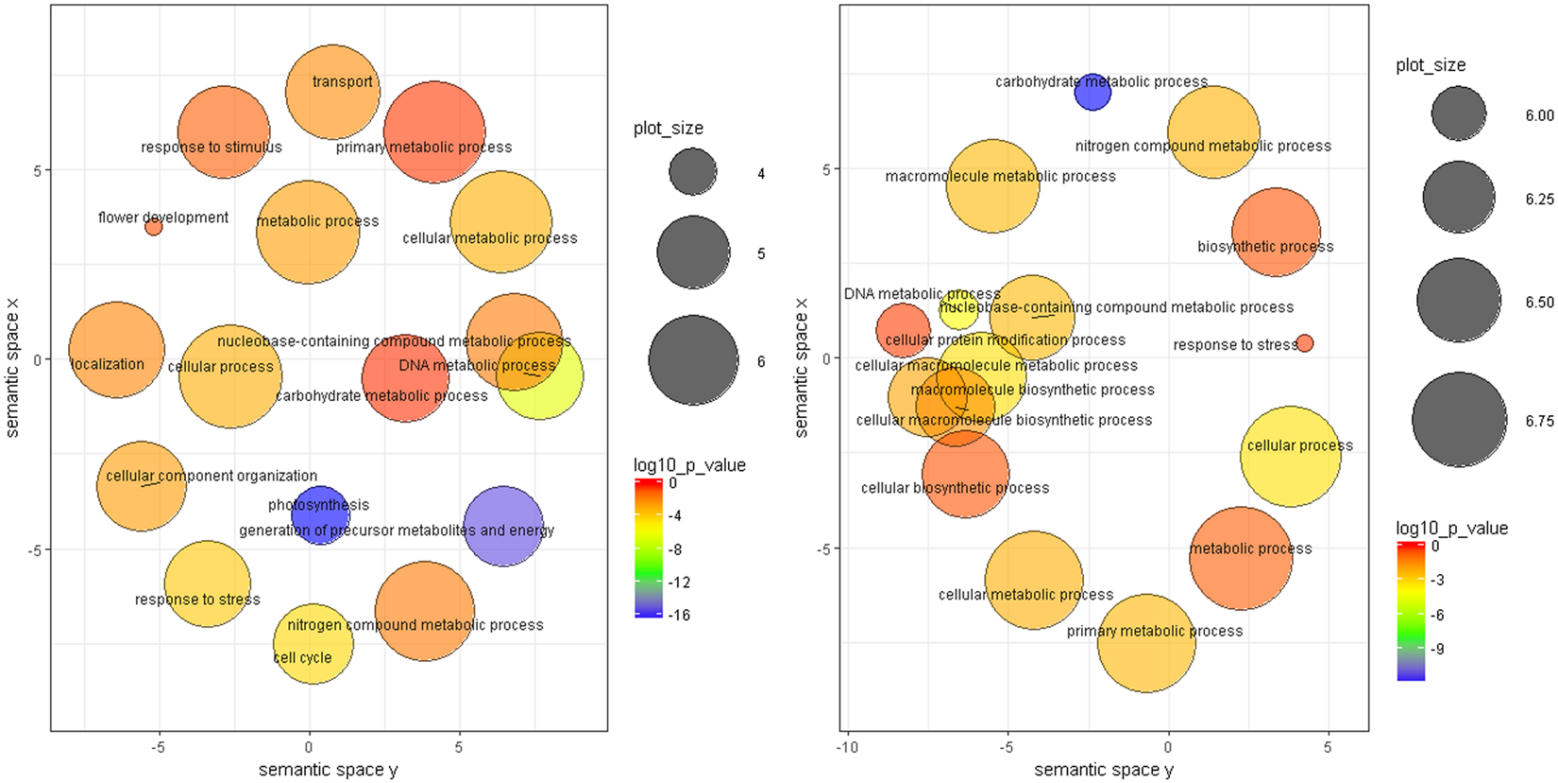
GO enrichment analysis visualized by REVIGO. Significantly enriched GO terms related to biological processes for transcripts exhibiting co-up-regulation (left one) and co-down-regulation (right one). Clustering of circles (representing GO terms) was based on semantic similarities to other GO terms in the gene ontology (larger circles represent more general terms whereas adjoin circles depict close relationship). Size and color of the circle represent the GO term frequency and the log_10_P-value for the enrichment derived from the AgriGO analysis (red higher, blue lower) respectively.

**Figure 6:**
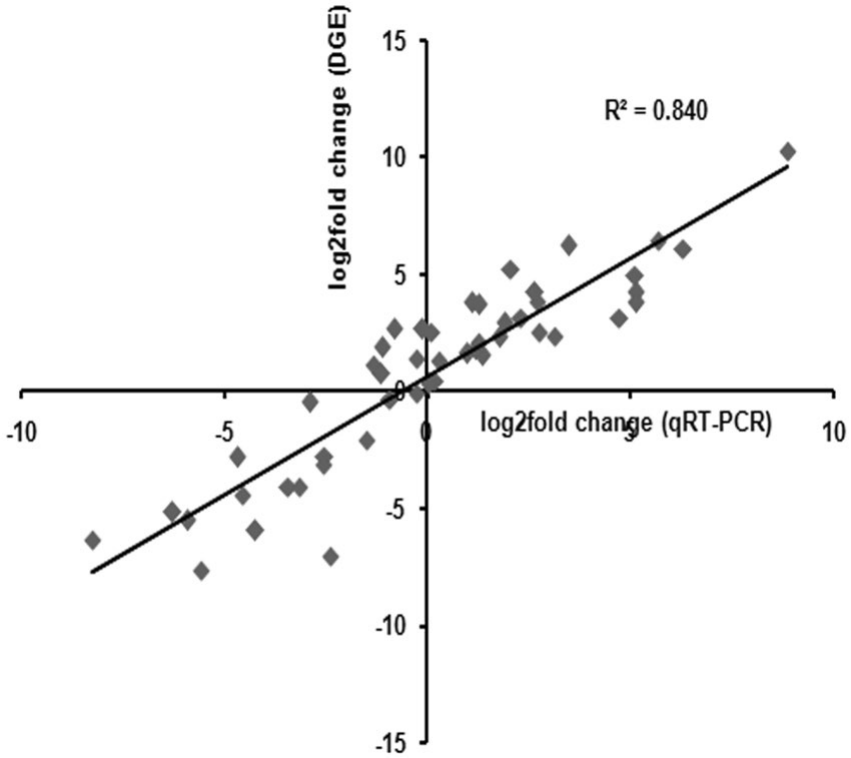
Correlation of qRT-PCR- and DGE- based expression profiles of transcripts. Expression profiles of 12 DEGs from each control vs. stress DGE data were selected for the analysis. X-axis represents average qRT - PCR data of three biological replicates. Y-axis represents log_2_fold change (control vs. stress).

GO term enrichment analysis was also performed for the sets of transcripts exhibiting co-up-regulation and co-down-regulation. 43 GO terms were found to be significantly (FDR ≤ 0.05) enriched for co-up-regulated genes and 21 for significantly (FDR ≤ 0.05) co-down-regulated genes (Supplementary file 5). In the category of ‘biological processes’, most significantly enriched GO term was ‘photosynthesis’ in commonly up-regulated transcripts (followed by ‘generation of precursor metabolites and energy’, ‘DNA metabolic process’, ‘cell cycle’, ‘response to stress’ and others) and ‘carbohydrate metabolic process’ in commonly down-regulated transcripts (followed by ‘DNA metabolic process’, ‘cell cycle’, ‘response to stress’, ‘cellular process’, ‘cellular macromolecule metabolic process’ and others) (Fig. 5). 12 transcripts were selected from each stress DGE data file for the authentication of DGE data through qRT-PCR and it was observed that the log_2_fold change values obtained through qRT-PCR and DGE data exhibited high level of correlation. The overall correlation coefficient (r) was 0.916 (r^2^ = 0.840) (Fig. 6), suggesting a good correlation.

The transcripts identified in this study that were showing common regulation in all four abiotic stresses might prove to be the possible targets for engineering stress-tolerance and stress adaptation in plants.

### Effect of abiotic stresses on artemisinin content and artemisinin biosynthesis pathway genes

**Figure 7:**
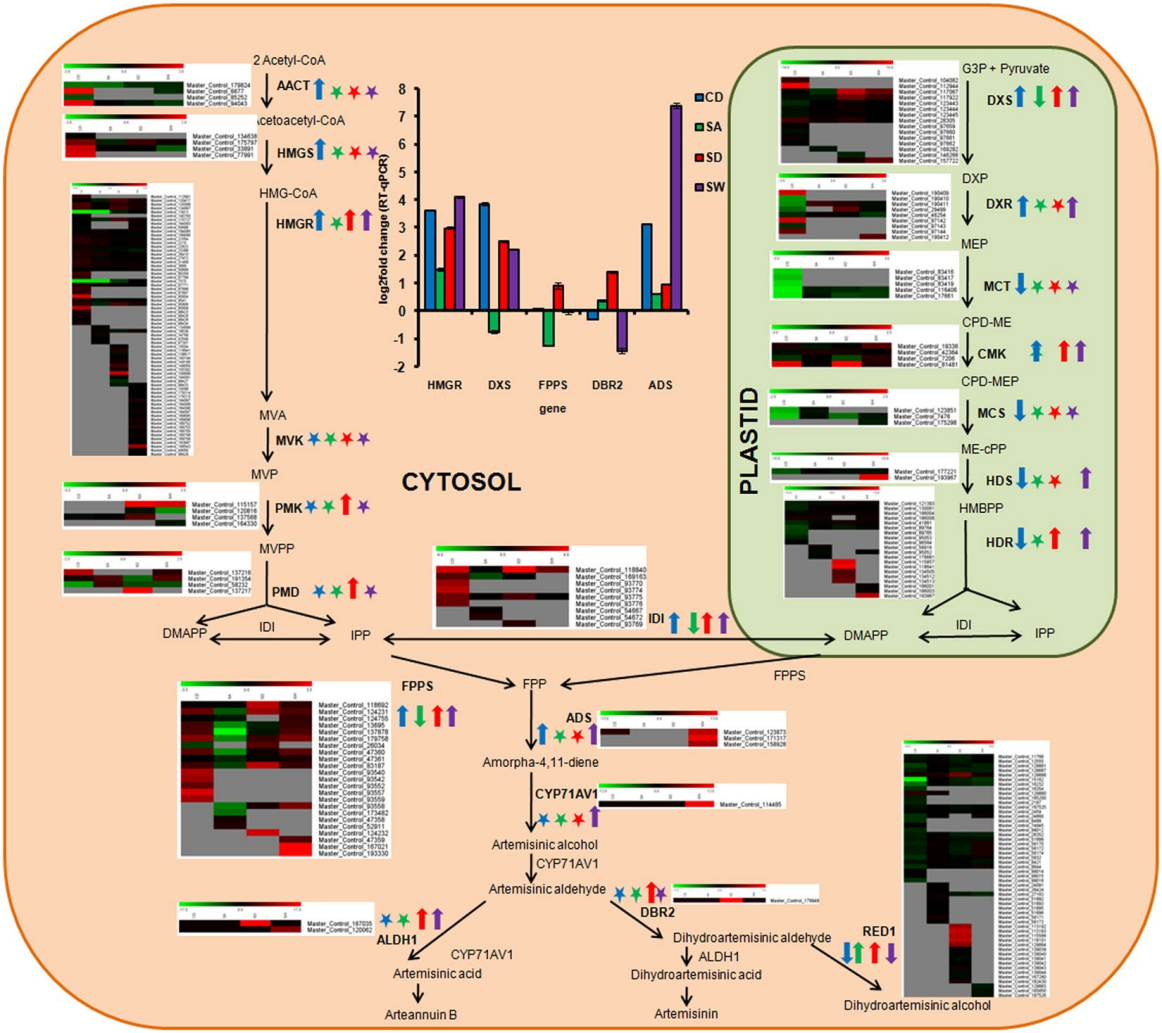
Differentially expressed artemisinin and other terpenoid biosynthetic pathway genes of *A. annua* during abiotic stresses. Expression patterns of artemisinin and other terpenoid biosynthetic pathway genes were represented based on average log_2_fold change values for the genes showing differential expression (calculated from transcripts with log_2_fold change >+1 or <−1). Upward arrows indicate up-regulation, downward arrows indicate down regulation and stars indicate neutral regulation or genes not identified. Graph represents validation of the expression pattern of five differentially expressed genes by RT-qPCR (data was analyzed by three independent repeats and standard deviations were shown with error bars). Color coding: blue for cold, green for salt, red for drought and purple for water-logging stress. Heat maps demonstrate the log_2_fold change values of all differentially expressed transcripts corresponding to a particular gene. Abbreviations used: CD,cold; SA,salinity; SD,drought; SW, water-logging; HMG-CoA, Hydroxymethylglutaryl-coenzyme A; MVA, mevalonic acid; MVP, 5-phosphomevalonate; MVPP, 5-diphosphomevalonate; DMAPP, dimethylallyl diphosphate; IPP, isopentenyl diphosphate; FPP, farnesyl diphosphate; AACT, acetoacetyl-coenzyme A thiolase; HMGS, 3-hydroxy-3-methyl-glutaryl coenzyme A synthase; HMGR, 3-hydroxy-3-methyl-glutaryl coenzyme A reductase; MVK, mevalonate kinase; PMK, phosphomevalonate kinase; PMD, diphosphomevalonate decarboxylase; IDI, isopentenyl diphosphate isomerase; FPPS, farnesyl diphosphate synthase; G3P, glyceraldehyde-3-phosphate; DXP, 1-deoxy-d-xylulose-5-phosphate; MEP, 2-C-methyl-d-erythritol-4-phosphate; CDP-ME, 4-(cytidine 5′-diphospho)-2-C-methyl-d-erythritol; CDP-MEP, 2-phospho-4-(cytidine 5′-dipospho)-2-C-methyl-d-erythritol; ME-cPP, 2-C-methyl-d-erythritol-2,4-cyclodiposphate; HBMPP, hydroxymethylbutenyl-4-diphosphate; DXS, 1-deoxy-d-xylulose-5-phosphate synthase; DXR, 1-deoxy-dxylulose-5-phosphate reductoisomerase; MCT, 2-C-methyl-d-erythritol-4-(cytidyl-5-diphosphate) transferase; CMK, 4-cytidine 5′-diphospho-2-C-methyl-d-erythritol kinase; MCS, 2-C-methyl-d-erythritol-2,4-cyclodiphosphate synthase; HDS, hydroxy-2-methyl-2-(E)-butenyl 4-diphosphate synthase; HDR, hydroxy-2-methyl-2-(E)-butenyl 4-diphosphate reductase, ADS, amorpha-4,11-diene synthase; ALDH1, aldehyde dehydrogenase 1; CYP71AV1, amorphadiene-12-hydroxylase; DBR2, artemisinic aldehyde reductase^17^.

**Figure 8:**
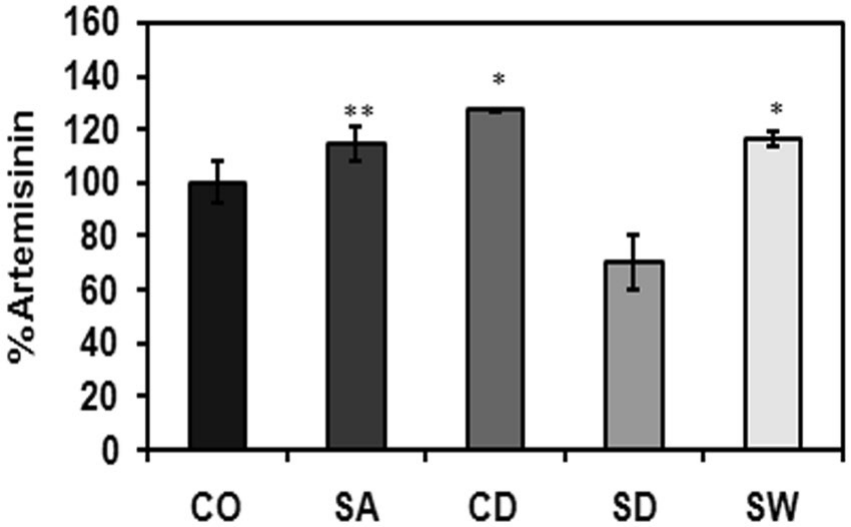
Estimation of artemisinin content. Artemisinin content was quantified as % dry weight of the leaves in the leaves of *A. annua* (CO) control, (SA) salinity, (CD) cold, (SD) drought and (SW) water-logging samples. The data was analyzed by three independent repeats, and standard deviations were shown with error bars. Significant differences between control and stress were indicated by ‘*’ for p-value ≤ 0.05 and ‘**’ for p-value ≤ 0.001.

Artemisinin, a sesquiterpene lactone, is synthesized from isopentenyl diphosphate and dimethylallyl diphosphate which themselves originate from cytosolic mevalonate pathway and plastidial non-mevalonate pathway/MEP pathway^17^. Expression pattern of various artemisinin biosynthetic genes in different abiotic stresses and validation of some of them through qRT-PCR is represented in Fig. 7. Several reports are there which suggest that artemisinin accumulation and biosynthesis is influenced by the environmental conditions. Modulation of artemisinin accumulation by various hormonal treatments, DMSO elicitation, chilling, salt, water deficit etc has already been reported^17^. In the present study, artemisinin content was increased by 14.68% in salt stress, 16.14% in water-logging stress and 27.16% in cold stress (Fig. 8). However, it was decreased by 29.56% in drought stress. Many artemisinin biosynthetic pathway genes exhibited enhanced expression in different abiotic stresses. It has been suggested by several researchers that dihydroartemisinic acid, which is the immediate precursor of artemisinin, acts as free radical scavenger and help the plants get adapted to stressful conditions by quenching high levels of singlet oxygen and, consequently resulting into increased production of artemisinin as the stable end product^41^. In the present study, the results obtained for salt, water-logging and cold stresses were in line with the above hypothesis. But the artemisinin content was lower in case of drought stress. Yadav *et al*.^17^ investigated the effect of prolonged water stress on artemisinin accumulation and found that artemisinin content decreases when stress treatment is continued for longer periods^17^. An increase in artemisinin content in response to salt stress has been reported by many researchers^18,42^. Effect of water-logging stress on artemisinin accumulation has not been investigated yet. Yang *et al*.^41^ studied the effect of 24 hr water-logging treatment on *A. annua in vitro* plants and reported that *DBR2* and *CPR* genes were up-regulated whereas other pathway genes did not show any significant change in expression^41^. Early induction of senescence due to water-logging stress in the present study might be the reason for enhanced artemisinin content and increased expression of the artemisinin biosynthetic pathway genes^41^.

It has been reported that when *in vitro A. annua* plants were kept at 4 °C for 24 hrs, all the major pathway genes including *HMGR, FPPS, DXS, DXR, CYP71AV1* and *ADS* were significantly up-regulated^41^. Yin *et al*.^43^ have also observed that chilling enhanced expression of *ADS* and *CYP71AV1* genes and increased artemisinin accumulation^43^. Thus, it was observed that artemisinin content (except for severe drought) and biosynthetic pathway genes are generally increased in prolonged abiotic stresses.

## Conclusion

In the present study, high-throughput sequencing was used to generate a comprehensive transcriptome resource for *A. annua* under four abiotic stresses (salt, cold, drought and water-logging). Comparative transcriptome analysis revealed many genes commonly or specifically regulated by different abiotic stresses. Some new genes were also identified which might be of interest in exploring the stress tolerance of the plant against climatic changes. Hence, this data represents a fully characterized *A. annua* transcriptome, providing new insights into plant responses to unfavourable environmental conditions, new leads for functional studies of genes involved in multiple abiotic stresses and potential candidate genes for metabolic engineering of multiple stress tolerance in *A. annua* with unaltered artemisinin content.

## Methods

### Plant stress treatment and Sampling

Mature seeds of *Artemisia annua* var. ‘CIM-Arogya’^44^ were obtained from National Gene Bank for Medicinal and Aromatic Plants (NGBMAP) maintained at the Central Institute of Medicinal and Aromatic Plants in India and sown in four square feet large nursery bed. Two months old seedlings were singled out, transferred to pots and allowed to acclimatize for a period of 15 days before stress treatment. Ten plants were subjected to each stress treatment along with natural control. For salt treatment, plants were irrigated with 100 ml of 100 mM NaCl solution continuously for ten days and then on every 3^rd^ day during the entire stress treatment period of 2 months. For 66% drought treatment, plants were irrigated in such a manner so as to maintain a moisture level of 34% of water holding capacity of the soil during the entire stress treatment period (2 months). For cold treatment, plants were kept in the cold room at 4 °C for 2 months with appropriate lightening. Samples were collected after completion of the treatment period. Plants were given water-logging stress after 2 months of transplantation since the morphology of the plants look severely affected by prolonged treatment. Plants were kept immersed in water such that the water level was 2–2.5 inches above the soil level throughout the stress period and sampled after 15 days. Plants maintained in natural conditions with normal watering (maintaining moisture content nearly equal to the water holding capacity of the soil) served as control. Samples were collected and pooled for sequencing in triplicates. Samples were stored in RNAlater (Sigma Aldrich) at −80 °C.

### RNA isolation and library preparation for transcriptome

Total RNA isolation from leaf samples was performed using TRI reagent (Sigma Aldrich). Nanodrop Spectrophotometer and Qubit Fluorometer were used for estimating RNA concentration and purity while RNA integrity was analysed on Bioanalyzer chip (Agilent). RNA with optimal purity, yield and integrity (RNA integrity number > 6.5) was used for library preparation. Illumina TruSeq RNA library protocol (as mentioned in “TruSeq RNA Sample Preparation Guide”) was used for transcriptome library construction for sequencing (Part # 15008136; Rev. A; Nov 2010). Purification of poly-A enriched RNA was carried out from 1 µg of total RNA and it was fragmented for 2 minutes at 94 °C in the presence of divalent cations followed by reverse transcription using Superscript III Reverse transcriptase by priming with random hexamers. DNA Polymerase I and RnaseH were used for second strand cDNA synthesis and Agencourt Ampure XP SPRI beads (Beckman Coulter) for cleaning up the cDNA. End reparation and 3′ end adenylation was performed for the cDNA molecules followed by ligation of illumina adapters. After ligation, SPRI cleanup was carried out followed by library amplification by 8 cycles of PCR to enrich the adapter ligated fragments. Quantification and qualitative validation for the prepared library were performed using Nanodrop and High Sensitivity Bioanalyzer Chip (Agilent), respectively.

### Sequencing, *de novo* assembly and functional annotation

The library was sequenced using Illumina Nextseq 500 platform producing 32.9, 45.8, 64.8, 65.4, 73.3 Mbp of 151-bp paired-end reads for control, salt, cold, drought and water-logging stress samples, respectively. Quality of raw reads was checked by FastQC program (http://www.bioinformatiSample1.babraham.ac.uk/projects/fastqc/). Raw reads obtained after sequencing were filtered to remove adaptor and low quality bases to obtain processed reads. Processed reads were used for *de novo* assembly with Trinity, a short read assembling program^45^, for default k-mers i.e. 25. In brief, firstly contigs were generated by combining reads with certain length of overlap. The reads used were then mapped back to contigs. Finally, the contigs were connected to generate unigenes. In this way, Trinity generated unique transcripts. Trinity assembly is based on the de Bruijn graph^46^. The Trinity assembled transcripts with sequence lengths > = 300 bp were considered for downstream analysis. Clustering of these transcripts with 95% identity was carried out using CD-HIT. The transcripts were annotated against all viridiplantae kingdom protein sequences (from Uniprot Protein Database) using NCBI BLAST 2.2.29^47^. Those Transcripts with more than 30% identity as cut off were taken for further analysis. GO annotations for the transcripts were retrieved from Uniprot database. More than one GO term may be assigned to each annotated sequence that may fall in either same or different GO category (Molecular Function, Biological Process and Cellular Component). Pathway Analysis was performed by using KAAS Server^48^. *Arabidopsis thaliana* (thale cress) and *Solanum lycopersicum* (tomato) were used as reference organisms from the available strains in the database. Annotations were retrieved from KEGG.

### Transcript abundance measurement

Transcripts of all the samples with length > = 300 bp were combined and clustering was performed at 95% identity using CD-HIT, a master control transcript data containing unigenes was thus generated, then alignment of reads of control and treated samples was carried out using Bowtie2 tool and a read count profile was generated. Differential gene expression (DGE) was obtained using DeSeq software (http://www-huber.embl.de/users/anders/DESeq/)^49^ comparing each stress sample with the control sample. For each comparison between control and a stress sample, three expression profiles were generated; transcripts expressed in both control and stress sample, transcripts expressed only in stress and transcripts expressed only in control. Those transcripts that were expressed in both samples involved in comparison were further classified according to their expression pattern (up-regulated, down-regulated and neutral). The negative binomial distribution method was applied for calculating p values and Benjamini-Hochberg method was chosen to adjust for multiple tests. DEGs with p value < = 0.05 were considered significant.

### DGE enrichment analysis

Gene ontology term enrichment analysis was performed using AgriGO (http://bioinfo.cau.edu.cn/agriGO/) supplemented by REVIGO (http://revigo.irb.hr/) visualization toolbox^50^. Enriched GO terms were statistically analyzed by Fisher’s exact test with Yekutieli multi-testing adjustment and significance level of 0.05. The significantly enriched pathways for DEGs were determined by the KEGG Orthology-Based Annotation System (KOBAS) (http://kobas.cbi.pku.edu.cn/home.do)^51^. Hyper geometric distribution was used for p-value calculation and Benjamini and Hochberg method for FDR correction. *Arabidopsis thaliana*, being a model organism was used as background species.

### Validation by qRT-PCR and PCR amplification

Total RNA isolation was carried out from all 5 samples, one control and 4 stress samples using TRIzol method and cDNA synthesis Kit (ThermoScientific, USA) was used for synthesis of template cDNAs from 2 µg of total RNAs. qRT-PCR was performed following the SYBR Green chemistry (Maxima SYBR Green 2 × PCR Master Mix, ThermoScientific, Waltham MA, US) and Fast Real Time PCR system (7900HT Applied Biosystems, USA) for validation of the Illumina sequencing data following the protocol described by Rastogi *et al*.^19^. Relative mRNA levels were quantified with respect to the endogenous control gene ‘actin’ of *A. annua*. All the experiments were repeated using three biological replicates and statistical analysis (±Standard Deviation) of data was carried out. For PCR amplification of stress specific transcripts, primers were designed from the transcriptome sequences and cDNAs synthesized above were used as template. Amplification conditions were as: 95 °C for 1 min followed by 35 cycles of 95 °C for 30 sec, annealing temperature ranging from 48 °C–55 °C for 30 sec, 72 °C for 1 min 30 sec, followed by a final extension of 5 mins at 72 °C. 1.2% agarose gel was prepared to electrophorese and visulalize the amplified product.

### Artemisinin extraction and analysis

Extraction of artemisinin was carried out following the protocol mentioned by Misra *et al*. while Thin layer chromatography technique was used for its estimation^52^. Leaves from all the plants belonging to same treatment group were collected, pooled and used for artemisinin estimation in triplicates. Statistical significance was determined by one-way ANOVA with Student’s t-test at a significance level of 0.05 in Excel software.

### Availability of data

Sequence data generated for the present study has been deposited to NCBI Short Read Archive. The bioproject ID assigned is: PRJNA352660 (http://www.ncbi.nlm.nih.gov/bioproject/352660).

## Electronic supplementary material

supplementary file 1

Dataset 1

Dataset 2

Dataset 3

Dataset 4

Dataset 5

Dataset 6

Dataset 7

Dataset 8

Dataset 9

Dataset 10
