# Supplementary material for: Transcriptome changes induced by abiotic stresses in Artemisia annua
Source: Sci Rep. 2018 Feb 21;8:3423. doi: 10.1038/s41598-018-21598-1 (PMC5821844; doi:10.1038/s41598-018-21598-1)
Supplement: Supplementary file 1 [file 41598_2018_21598_MOESM1_ESM.pdf]

Transcriptome changes induced by abiotic stresses in *Artemisia annua*

SUPPLEMENTARY FILE 1

Divya Vashisth[1], Ritesh Kumar[1], Shubhra Rastogi[2], Vikas Kumar Patel[3], Alok Kalra[3], Madan Mohan Gupta[4], Anil Kumar Gupta[5], Ajit Kumar Shasany[1]*

[1] Biotechnology Division, CSIR-Central Institute of Medicinal and Aromatic Plants, P.O. CIMAP, Lucknow- 226015, U.P., India

[2] Department of Biochemistry, University of Lucknow, Lucknow 226007, U. P., India

[3] Microbial Technology Division, CSIR-Central Institute of Medicinal and Aromatic Plants, P.O. CIMAP, Lucknow- 226015, U. P., India.

[4]Analytical Chemistry Division, CSIR-Central Institute of Medicinal and Aromatic Plants, P.O. CIMAP, Lucknow- 226015, U.P, India.

[5] Genetics and Plant Breeding Division, CSIR-Central Institute of Medicinal and Aromatic Plants, P.O. CIMAP, Lucknow- 226015, U.P., India.

*Correspondence:

Ajit Kumar Shasany,

Biotechnology Division,

CSIR-Central Institute of Medicinal and Aromatic Plants,

P.O. CIMAP, Lucknow-226015, U.P., India

Phone: 91-522-2718548

Fax: 91-522-2342666

Email: ak.shasany@cimap.res.in, akshasany@yahoo.com

Table S1. Raw data Processing and QC

Samples	Platform	Type of reads	Total number of Raw Reads	Total number of Processed Reads
**CO**	Illumina NextSeq	2 x Paired end (151bp max)	32931686	30250682
**SA**	Illumina NextSeq	2 x Paired end (151bp max)	45836540	43173270
**CD**	Illumina NextSeq	2 x Paired end (151bp max)	64825268	60142686
**SD**	Illumina NextSeq	2 x Paired end (151bp max)	65407218	61451072
**SW**	Illumina NextSeq	2 x Paired end (151bp max)	73341744	69453942

Table S2. Transcriptome Denovo Assembly Statistics

Sample Name	CO	SA	CD	SD	SW
Tools used		Trinity			
Hash length		25			
Transcripts Generated	89362	81328	76337	90470	96493
Maximum Transcript Length	11127	15640	13687	15548	15587
Minimum Transcript Length	300	301	301	301	301
Average Transcript Length	1198.4	1032.6	1001.7	1008.7	1014.3
Median Transcript Length	2598	321.5	1839	2731	1818
Total Transcripts Length	107087281	83982122	76467687	91256868	97876620
Total Number of Non-ATGC Characters	0	0	0	0	0
Transcripts >= 300bp	89362	81328	76337	90470	96493
Transcripts > 500bp	65888	53343	51107	60063	63822
Transcripts > 1Kb	42041	29592	27817	32123	34532
Transcripts > 10Kb	15	23	4	15	12
N50 value	1697	1491	1396	1424	1438

Table S3. Transcriptome Annotation

Transcript Annotation Summary	Transcripts				
	CA	SA	CD	SD	SW
**Total Transcripts**	89362	81328	76337	90470	96493
**Total Transcripts (COG/Cluster)**	70669	73777	70546	82135	88109
**Total Annotated Transcripts**	41618	38047	39863	43947	45091
**Total Unannotated Transcripts**	29051	35730	30683	38188	43018

Table S4. Transcript abundance of transcription factors

	CO	SA	CD	SD	SW
ABI3VP1	0	0	0	0	0
Alfin-like	4	1	1	1	1
AP2/ERF/DREB/AP2-EREBP	54	45	70	59	74
ARF	30	45	49	45	48
ARID	3	1	1	0	0
AUX/IAA	27	19	31	36	31
B3	2	1	0	6	0
BBR/BPC	10	8	8	9	8
BES/BZR	8	0	2	0	1
bHLH	29	19	25	35	29
BSD	2	2	2	3	4
BZIP	27	30	25	29	32
C2H2	13	13	13	16	15
C3H	24	30	21	34	26
CAMTA	2	8	3	5	5
CCAAT	3	5	3	4	6
CO-like	6	6	7	5	7
CPP	2	1	0	0	0
CSD	3	3	1	3	3
DBB	1	7	6	6	5
DDT	6	5	5	5	9
Dof	10	9	8	9	9
E2F-DP	3	3	2	1	1
EIL	2	2	3	2	2
FAR1	17	20	11	16	19
FHA	4	4	3	2	3
GATA	3	1	4	5	5
GNAT	3	2	1	0	2
GRAS	9	6	10	10	12
GRF	3	5	4	4	3
HB	43	38	39	35	40

ZF-HD	5	4	5	6	6
HMG	5	6	3	4	4
HSF	17	18	18	16	18
Jumonji	3	5	4	5	5
LIM	1	1	1	1	1
LOB/LBD	2	2	4	3	3
LSD	1	1	1	1	2
MADS	26	9	23	20	13
MBF1	1	1	1	1	1
M-type	6	5	3	4	4
MYB/MYB-related	93	63	65	94	96
NAC	37	31	45	43	39
NF-X1	6	3	4	2	4
Nin-like/NLP	6	3	5	3	2
PHD	19	15	13	17	26
PLATZ	1	1	1	1	3
Pseudo ARR-B	16	3	2	5	4
RAV	1	0	1	1	1
RWP-RK	16	10	5	12	17
SAP	6	3	3	3	3
SBP	4	2	5	8	5
TAZ	2	1	3	2	1
TCP	9	12	10	7	8
TRAF	1	1	3	2	2
Trihelix	0	0	1	0	0
VOZ	0	0	0	0	0
Whirly	1	1	2	1	1
WRKY	63	76	85	81	95
YABBY	0	0	4	1	2
MYC	3	4	6	4	5
Cycloidea-like	4	2	3	9	7
LHY	6	6	6	11	6
Other transcription factors	58	62	53	70	70

Table S5. Total Transcription factors up regulated in different stress samples (with log2fold change>=1 and p value<=0.05)

Transcription factor	CD	SA	SD	SW
MYB and myb related	4	3	4	1
NAC	3	1	4	4
WRKY	2	0	4	3
YABBY transcription factor	1	0	1	1
AP2/ERF/AP2-EREBP	13	0	10	8
Auxin response factor	1	0	1	1
Dof-type zinc finger DNA-binding family protein	1	0	1	2

FAR1; Zinc finger, SWIM-type	0	1	1	2
hb/zf-HD/HD-zip	4	0	7	7
BES1/BZR1	0	0	1	1
bHLH	0	0	2	3
bZIP	0	0	1	0
Ccaat-binding transcription factor	0	1	2	0
Cycloidea-like protein	0	0	2	0
Cys2/His2-type zinc finger protein	3	0	0	0
DREB	1	0	0	0
GRAS family transcription factor	0	0	2	3
GRF zinc finger protein	0	1	0	0
Heat shock transcription factor	0	0	1	0
heat stress transcription factor	0	0	1	2
HMG	0	0	1	0
JUMONJI	3	0	0	0
Late elongated hypocotyl-like	0	0	2	5
LOB domain containing protein	2	0	0	0
MADS-box transcription factor	3	0	1	0
squamosa promoter binding protein	0	0	9	2
TCP	1	0	0	0
zinc finger CCCH protein	0	1	0	0
Zinc finger protein CONSTANS-LIKE	0	1	0	1

Table S6 Unigenes expressed only in stress and common in 3 or more stress.

Unigene_ID	Annotation
Master_Control_78545	hypothetical protein B456_003G053000 [*Gossypium raimondii*]
Master_Control_107193	Unknown
Master_Control_107251	Serine/Threonine kinase, plant-type protein, putative
Master_Control_173524	Unknown
Master_Control_176756	Unknown
Master_Control_121391	Unknown
Master_Control_167914	Uncharacterized protein
Master_Control_170559	Unknown
Master_Control_182019	Coffea canephora DH200=94 genomic scaffold, scaffold_8 (ribonuclease III activity)
Master_Control_182023	uncharacterized protein
Master_Control_79960	Putative non-LTR retroelement reverse transcriptase
Master_Control_125981	uncharacterized protein
Master_Control_182017	Coffea canephora DH200=94 genomic scaffold, scaffold_8 (ribonuclease III activity)
Master_Control_48802	uncharacterized protein (zinc ion binding)
Master_Control_54707	Unknown
Master_Control_131945	short chain alcohol dehydrogenase, putative
Master_Control_95060	Glucose-1-phosphate adenylyltransferase
Master_Control_100008	Endoglucanase (EC 3.2.1.4)

Master_Control_118858	kelch repeat protein
Master_Control_119991	Unknown
Master_Control_122964	Coffea canephora DH200=94 genomic scaffold, scaffold_41
Master_Control_127484	DNA binding protein, putative
Master_Control_128238	Homocysteine S-methyltransferase 1
Master_Control_130527	Pentatricopeptide repeat-containing protein
Master_Control_131796	Chromomethylase(DNA methyltransferase activity)
Master_Control_135752	Unknown
Master_Control_136107	Uncharacterized protein with oxidoreductase activity
Master_Control_137283	Beta-amyrin synthase
Master_Control_139769	GRAS family transcription factor
Master_Control_143435	Xyloglucan endotransglucosylase/hydrolase (EC 2.4.1.207)
Master_Control_165088	Unknown
Master_Control_172275	Uncharacterized protein with hydrolase activity
Master_Control_189133	Putative helicase
Master_Control_35350	Unknown
Master_Control_69406	Uncharacterized protein involved in signal transduction
Master_Control_69433	Spermidine synthase 3 isoform 3
Master_Control_71485	uncharacterized protein
Master_Control_72796	Unknown
Master_Control_78764	Putative helicase
Master_Control_82690	Putative squalene monooxygenase
Master_Control_85614	uncharacterized protein

Table S7. Transcripts unexpressed in all the stresses.

Unigene ID	Annotation
Master_Control_29067	40S ribosomal protein S27
Master_Control_18563	ATP synthase subunit a
Master_Control_20652	Beta-carotene isomerase D27 (Dwarf 27)
Master_Control_14841	Beta-galactosidase (EC 3.2.1.23)
Master_Control_8160	Ca2+ activated outward rectifying K+ channel 6 isoform 1
Master_Control_26067	Cation chloride cotransporter
Master_Control_20232	Component of cytosolic 80S ribosome and 40S small subunit
Master_Control_5462	Cytochrome b
Master_Control_27331	Cytochrome c oxidase subunit 1 (EC 1.9.3.1)
Master_Control_21339	Cytochrome c oxidase subunit 1 (EC 1.9.3.1)
Master_Control_8328	Cytochrome c oxidase subunit 1 (EC 1.9.3.1) (Fragment)
Master_Control_3961	Cytochrome c oxidase subunit 2
Master_Control_26758	Cytochrome c oxidase subunit 3
Master_Control_23696	Cytochrome P450-dependent monooxygenase-like protein
Master_Control_25167	DNA binding protein-like (Os07g0190600 protein)
Master_Control_31202	DNA topoisomerase 6 subunit A (EC 5.99.1.3)
Master_Control_14932	DNase I superfamily protein, putative

Master_Control_18386	Endonuclease/exonuclease/phosphatase family protein
Master_Control_17874	Endonuclease/exonuclease/phosphatase family protein
Master_Control_17024	Fiber protein Fb9 (Fragment)
Master_Control_25679	Germacrene A oxidase (BsGAO)
Master_Control_30970	Glyoxalase/Bleomycin resistance protein/Dioxygenase superfamily protein isoform 2
Master_Control_17483	Hemoglobin
Master_Control_12113	Histone deacetylase 2a-like
Master_Control_7001	Homeobox-leucine zipper protein ROC5
Master_Control_23700	Leucine-rich repeat transmembrane protein kinase, putative
Master_Control_9413	Membrane acyl-CoA binding protein
Master_Control_3853	NADH-ubiquinone oxidoreductase chain 1 (EC 1.6.5.3)
Master_Control_14782	P-loop containing nucleoside triphosphate hydrolases superfamily protein
Master_Control_16603	Predicted protein
Master_Control_14551	Protease-related family protein
Master_Control_7537	Protein kinase APK1B, chloroplast, putative (EC 2.7.10.2)
Master_Control_11426	Putative AP endonuclease/reverse transcriptase
Master_Control_6711	Putative homogentisate 1,2-dioxygenase (Fragment)
Master_Control_12126	Putative non-LTR retroelement reverse transcriptase
Master_Control_1645	Putative PIF1 DNA helicase/replication protein A1-like protein
Master_Control_6596	Putative polyprotein
Master_Control_28236	Putative polyprotein
Master_Control_24989	Putative retrotransposon
Master_Control_8829	Putative reverse transcriptase
Master_Control_5883	Putative reverse transcriptase (Retrotransposon protein, putative, unclassified)
Master_Control_7757	Putative senescence-associated protein (Fragment)
Master_Control_4261	Receptor-like kinase
Master_Control_19288	Retrotransposon protein, putative, unclassified
Master_Control_16779	Retrotransposon, unclassified-like protein
Master_Control_30711	Reverse transcriptase (Fragment)
Master_Control_22069	RNA-directed DNA polymerase (Reverse transcriptase)
Master_Control_5786	RuBisCO subunit binding-protein beta subunit (Fragment)
Master_Control_26955	Senescence-associated protein
Master_Control_22962	Steroid binding protein, putative (EC 1.3.1.74)
Master_Control_26454	Transcription factor GbMYB1
Master_Control_7672	GDP-L-galactose phosphorylase (Uncharacterized protein)
Master_Control_13850	Protein kinase APK1A, chloroplastic (Uncharacterized protein)
Master_Control_29539	Putative uncharacterized protein
Master_Control_16718	Putative uncharacterized protein
Master_Control_31796	Putative uncharacterized protein
Master_Control_27544	Putative uncharacterized protein
Master_Control_14911	Putative uncharacterized protein
Master_Control_27695	Putative uncharacterized protein
Master_Control_307	Putative uncharacterized protein

Master_Control_27813	Putative uncharacterized protein
Master_Control_3395	Putative uncharacterized protein
Master_Control_25143	Putative uncharacterized protein
Master_Control_19373	Putative uncharacterized protein
Master_Control_23939	Putative uncharacterized protein
Master_Control_341	Putative uncharacterized protein
Master_Control_30538	Putative uncharacterized protein
Master_Control_25407	Putative uncharacterized protein
Master_Control_2044	Putative uncharacterized protein
Master_Control_27689	Putative uncharacterized protein
Master_Control_1030	Putative uncharacterized protein
Master_Control_21503	Putative uncharacterized protein
Master_Control_17523	Putative uncharacterized protein
Master_Control_7158	Putative uncharacterized protein
Master_Control_26078	Putative uncharacterized protein
Master_Control_30169	Putative uncharacterized protein
Master_Control_24348	Putative uncharacterized protein
Master_Control_14515	Putative uncharacterized protein
Master_Control_5488	Putative uncharacterized protein CTOS-1
Master_Control_6263	Putative uncharacterized protein Sb04g009250
Master_Control_81	Putative uncharacterized protein T2O9.150
Master_Control_29280	Uncharacterized protein
Master_Control_6229	Uncharacterized protein
Master_Control_27112	Uncharacterized protein
Master_Control_4863	Uncharacterized protein
Master_Control_30999	Uncharacterized protein
Master_Control_22236	Uncharacterized protein
Master_Control_32342	Uncharacterized protein
Master_Control_10332	Uncharacterized protein
Master_Control_19801	Uncharacterized protein
Master_Control_25170	Uncharacterized protein
Master_Control_23827	Uncharacterized protein
Master_Control_20584	Uncharacterized protein
Master_Control_11606	Uncharacterized protein
Master_Control_3861	Uncharacterized protein
Master_Control_11142	Uncharacterized protein
Master_Control_1897	Uncharacterized protein
Master_Control_9905	Uncharacterized protein
Master_Control_1002	Uncharacterized protein
Master_Control_11182	Uncharacterized protein
Master_Control_3969	Uncharacterized protein
Master_Control_1717	Uncharacterized protein
Master_Control_5517	Uncharacterized protein
Master_Control_2023	Uncharacterized protein
Master_Control_7240	Uncharacterized protein

Master_Control_18030	Uncharacterized protein
Master_Control_7362	Uncharacterized protein
Master_Control_7995	Uncharacterized protein
Master_Control_6461	Uncharacterized protein
Master_Control_19654	Uncharacterized protein
Master_Control_26802	Uncharacterized protein
Master_Control_3421	Uncharacterized protein
Master_Control_20462	Uncharacterized protein
Master_Control_18290	Uncharacterized protein
Master_Control_21970	Uncharacterized protein
Master_Control_23555	Uncharacterized protein
Master_Control_13055	Uncharacterized protein
Master_Control_25081	Uncharacterized protein
Master_Control_6295	Uncharacterized protein
Master_Control_6981	Uncharacterized protein
Master_Control_12076	Uncharacterized protein
Master_Control_25562	Uncharacterized protein
Master_Control_6056	Uncharacterized protein
Master_Control_25958	Uncharacterized protein
Master_Control_13796	Uncharacterized protein
Master_Control_1019	Uncharacterized protein
Master_Control_10388	Uncharacterized protein
Master_Control_26665	Uncharacterized protein
Master_Control_17270	Uncharacterized protein
Master_Control_28071	Uncharacterized protein
Master_Control_22801	Uncharacterized protein
Master_Control_301	Uncharacterized protein
Master_Control_18983	Uncharacterized protein
Master_Control_31386	Uncharacterized protein
Master_Control_27727	Uncharacterized protein
Master_Control_24036	Uncharacterized protein
Master_Control_14657	Uncharacterized protein
Master_Control_17154	Uncharacterized protein
Master_Control_25232	Uncharacterized protein
Master_Control_11232	Uncharacterized protein
Master_Control_16527	Uncharacterized protein
Master_Control_26626	Uncharacterized protein
Master_Control_14236	Uncharacterized protein
Master_Control_20566	Uncharacterized protein
Master_Control_5383	Uncharacterized protein
Master_Control_20706	Uncharacterized protein
Master_Control_2228	Uncharacterized protein
Master_Control_26154	Uncharacterized protein
Master_Control_21476	Uncharacterized protein
Master_Control_13833	Uncharacterized protein

Master_Control_2233	Uncharacterized protein
Master_Control_25115	Uncharacterized protein
Master_Control_27525	Uncharacterized protein
Master_Control_22966	Uncharacterized protein
Master_Control_23523	Uncharacterized protein
Master_Control_28829	Uncharacterized protein
Master_Control_3841	Uncharacterized protein
Master_Control_16471	Uncharacterized protein
Master_Control_7917	Uncharacterized protein
Master_Control_5729	Uncharacterized protein
Master_Control_2586	Uncharacterized protein (Fragment)
Master_Control_8714	Uncharacterized protein (Fragment)
Master_Control_5685	Uncharacterized protein (Fragment)
Master_Control_8906	Uncharacterized protein (Fragment)
Master_Control_20181	Uncharacterized protein (Fragment)
Master_Control_27361	Uncharacterized protein (Fragment)
Master_Control_45	Uncharacterized protein (Fragment)
Master_Control_22077	Uncharacterized protein (Fragment)
Master_Control_17414	Uncharacterized protein (Fragment)
Master_Control_29057	Uncharacterized protein (Fragment)
Master_Control_1305	Coffea canephora DH200=94 genomic scaffold, scaffold_10611
Master_Control_5203	Coffea canephora DH200=94 genomic scaffold, scaffold_150
Master_Control_26820	Coffea canephora DH200=94 genomic scaffold, scaffold_16
Master_Control_13540	Coffea canephora DH200=94 genomic scaffold, scaffold_19
Master_Control_21528	Coffea canephora DH200=94 genomic scaffold, scaffold_22
Master_Control_11503	Coffea canephora DH200=94 genomic scaffold, scaffold_3
Master_Control_22861	Coffea canephora DH200=94 genomic scaffold, scaffold_38
Master_Control_17055	Coffea canephora DH200=94 genomic scaffold, scaffold_5
Master_Control_4426	Coffea canephora DH200=94 genomic scaffold, scaffold_5
Master_Control_28102	Coffea canephora DH200=94 genomic scaffold, scaffold_8
Master_Control_29518	Coffea canephora DH200=94 genomic scaffold, scaffold_9
Master_Control_11203	Coffea canephora DH200=94 genomic scaffold, scaffold_94
Master_Control_14734	Coffea canephora DH200=94 genomic scaffold, scaffold_98
Master_Control_13818	NA
Master_Control_18124	NA
Master_Control_24892	NA
Master_Control_26261	NA
Master_Control_15295	NA
Master_Control_15642	NA
Master_Control_7822	NA
Master_Control_31461	NA
Master_Control_15112	NA
Master_Control_30703	NA
Master_Control_3628	NA
Master_Control_5062	NA

Master_Control_29640	NA
Master_Control_17792	NA
Master_Control_17352	NA
Master_Control_1543	NA
Master_Control_14976	NA
Master_Control_8504	NA
Master_Control_21104	NA
Master_Control_17365	NA
Master_Control_6392	NA
Master_Control_18283	NA
Master_Control_5226	NA
Master_Control_10817	NA
Master_Control_1644	NA
Master_Control_9660	NA
Master_Control_20504	NA
Master_Control_30172	NA
Master_Control_26819	NA
Master_Control_18199	NA
Master_Control_1214	NA
Master_Control_7794	NA
Master_Control_30631	NA
Master_Control_24147	NA
Master_Control_29203	NA
Master_Control_20425	NA
Master_Control_10468	NA
Master_Control_11015	NA
Master_Control_10734	NA
Master_Control_20060	NA
Master_Control_3085	NA
Master_Control_122	NA
Master_Control_25871	NA
Master_Control_19470	NA
Master_Control_8562	NA
Master_Control_30	NA
Master_Control_27448	NA
Master_Control_7494	NA
Master_Control_1051	NA
Master_Control_30792	NA
Master_Control_15137	NA
Master_Control_14123	NA
Master_Control_31681	NA
Master_Control_20707	NA
Master_Control_13148	NA
Master_Control_11390	NA
Master_Control_631	NA

Master_Control_13321	NA
Master_Control_5544	NA
Master_Control_957	NA
Master_Control_14915	NA
Master_Control_26486	NA
Master_Control_26263	NA
Master_Control_28191	NA
Master_Control_16252	NA
Master_Control_2381	NA
Master_Control_1349	NA
Master_Control_19827	NA
Master_Control_10111	NA
Master_Control_7029	NA
Master_Control_27032	NA
Master_Control_22430	NA
Master_Control_27320	NA
Master_Control_21687	NA
Master_Control_19174	NA
Master_Control_17820	NA
Master_Control_6670	NA
Master_Control_4461	NA
Master_Control_4982	NA
Master_Control_25256	NA
Master_Control_3458	NA
Master_Control_8618	NA
Master_Control_27445	NA
Master_Control_10223	NA
Master_Control_8965	NA
Master_Control_24293	NA
Master_Control_1418	NA
Master_Control_14314	NA
Master_Control_30851	NA
Master_Control_32293	NA
Master_Control_17502	NA
Master_Control_18593	NA
Master_Control_25633	NA
Master_Control_19322	NA
Master_Control_13870	NA
Master_Control_26178	NA
Master_Control_18987	NA
Master_Control_9306	NA
Master_Control_23397	NA
Master_Control_23951	NA
Master_Control_15066	NA
Master_Control_18363	NA

Master_Control_22360	NA
Master_Control_21086	NA
Master_Control_23081	NA
Master_Control_25017	NA
Master_Control_20399	NA
Master_Control_1482	NA
Master_Control_24016	NA
Master_Control_25060	NA
Master_Control_14066	NA
Master_Control_3951	NA
Master_Control_2038	NA
Master_Control_31342	NA
Master_Control_5857	NA
Master_Control_6800	NA
Master_Control_29536	NA
Master_Control_7544	NA
Master_Control_442	NA
Master_Control_16871	NA
Master_Control_24164	NA
Master_Control_11427	NA
Master_Control_14666	NA
Master_Control_19073	NA
Master_Control_19961	NA
Master_Control_533	NA
Master_Control_28448	NA
Master_Control_10454	NA
Master_Control_16829	NA
Master_Control_12934	NA
Master_Control_22517	NA
Master_Control_6169	NA
Master_Control_13133	NA
Master_Control_16987	NA
Master_Control_17183	NA
Master_Control_4537	NA
Master_Control_10437	NA
Master_Control_17449	NA
Master_Control_7527	NA
Master_Control_12563	NA
Master_Control_21467	NA
Master_Control_27972	NA
Master_Control_24986	NA
Master_Control_32292	NA
Master_Control_31043	NA
Master_Control_7587	NA
Master_Control_11875	NA

Master_Control_19181	NA
Master_Control_27383	NA
Master_Control_21427	NA
Master_Control_23423	NA
Master_Control_20459	NA
Master_Control_17399	NA
Master_Control_8742	NA
Master_Control_1619	NA
Master_Control_28755	NA
Master_Control_713	NA
Master_Control_24809	NA
Master_Control_3485	NA
Master_Control_2477	NA
Master_Control_379	NA
Master_Control_30771	NA
Master_Control_27645	NA
Master_Control_24990	NA
Master_Control_1302	NA
Master_Control_28163	NA
Master_Control_29005	NA
Master_Control_5735	NA
Master_Control_2288	NA
Master_Control_21951	NA
Master_Control_24636	NA
Master_Control_9888	NA
Master_Control_13947	NA
Master_Control_11789	NA
Master_Control_20544	NA
Master_Control_18457	NA
Master_Control_30844	NA
Master_Control_30721	NA
Master_Control_15855	NA
Master_Control_4528	NA
Master_Control_7199	NA
Master_Control_21707	NA
Master_Control_15935	NA
Master_Control_25139	NA
Master_Control_17994	NA
Master_Control_7471	NA
Master_Control_18820	NA
Master_Control_8119	NA
Master_Control_4780	NA
Master_Control_27183	NA
Master_Control_6052	NA
Master_Control_20760	NA

Master_Control_26057	NA
Master_Control_12223	NA
Master_Control_28940	NA
Master_Control_27103	NA
Master_Control_9679	NA
Master_Control_8860	NA
Master_Control_31827	NA
Master_Control_29503	NA
Master_Control_19308	NA
Master_Control_3022	NA
Master_Control_3747	NA
Master_Control_5924	NA
Master_Control_8343	NA
Master_Control_15512	NA
Master_Control_5897	NA
Master_Control_27091	NA
Master_Control_4285	NA
Master_Control_14367	NA
Master_Control_21847	NA
Master_Control_4473	NA
Master_Control_4169	NA
Master_Control_5641	NA
Master_Control_13307	NA
Master_Control_4200	NA
Master_Control_12401	NA
Master_Control_26950	NA
Master_Control_21860	NA
Master_Control_24296	NA
Master_Control_24985	NA
Master_Control_12573	NA
Master_Control_21642	NA
Master_Control_7493	NA
Master_Control_6764	NA
Master_Control_27787	NA
Master_Control_6770	NA
Master_Control_30569	NA
Master_Control_24013	NA
Master_Control_10450	NA
Master_Control_2037	NA
Master_Control_2703	NA
Master_Control_26630	NA
Master_Control_11663	NA
Master_Control_4716	NA
Master_Control_20799	NA
Master_Control_2084	NA

Master_Control_16515	NA
Master_Control_15319	NA
Master_Control_25529	NA
Master_Control_25287	NA
Master_Control_7209	NA
Master_Control_1196	NA
Master_Control_5312	NA
Master_Control_29656	NA
Master_Control_19647	NA
Master_Control_28086	NA
Master_Control_19240	NA
Master_Control_12682	NA
Master_Control_3784	NA
Master_Control_26705	NA
Master_Control_5958	NA
Master_Control_5356	NA
Master_Control_19577	NA
Master_Control_5649	NA
Master_Control_11997	NA
Master_Control_7165	NA
Master_Control_26757	NA
Master_Control_27946	NA
Master_Control_30335	NA
Master_Control_30507	NA
Master_Control_311	NA
Master_Control_6742	NA
Master_Control_15715	NA
Master_Control_30579	NA
Master_Control_27673	NA
Master_Control_17765	NA
Master_Control_10902	NA
Master_Control_8313	NA
Master_Control_13456	NA
Master_Control_30061	NA
Master_Control_1516	NA
Master_Control_17062	NA
Master_Control_10882	NA
Master_Control_9758	NA
Master_Control_420	NA
Master_Control_30107	NA
Master_Control_28005	NA
Master_Control_8258	NA
Master_Control_122842	NA
Master_Control_3921	NA
Master_Control_30383	NA

Master_Control_11104	NA
Master_Control_3599	NA
Master_Control_22934	NA
Master_Control_31101	NA
Master_Control_6902	NA
Master_Control_28289	NA
Master_Control_29541	NA
Master_Control_4400	NA
Master_Control_23360	NA
Master_Control_26949	NA
Master_Control_264	NA
Master_Control_12580	NA
Master_Control_24047	NA
Master_Control_21635	NA
Master_Control_7108	NA
Master_Control_12306	NA
Master_Control_9420	NA
Master_Control_3945	NA
Master_Control_3455	NA
Master_Control_16771	NA
Master_Control_26953	NA
Master_Control_26957	NA
Master_Control_21192	NA
Master_Control_6953	NA
Master_Control_30893	NA
Master_Control_1526	NA
Master_Control_30108	NA
Master_Control_6757	NA
Master_Control_4526	NA
Master_Control_22970	NA
Master_Control_22506	NA
Master_Control_3249	NA
Master_Control_7355	NA
Master_Control_5785	NA
Master_Control_12108	NA
Master_Control_26858	NA
Master_Control_16034	NA
Master_Control_5972	NA
Master_Control_25127	NA
Master_Control_9511	NA
Master_Control_26193	NA
Master_Control_9897	NA
Master_Control_23864	NA
Master_Control_19875	NA
Master_Control_9326	NA

Master_Control_24629	NA
Master_Control_31844	NA
Master_Control_3845	NA
Master_Control_12834	NA
Master_Control_25652	NA
Master_Control_27607	NA
Master_Control_13059	NA
Master_Control_25408	NA
Master_Control_20612	NA
Master_Control_30231	NA
Master_Control_6017	NA
Master_Control_17805	NA
Master_Control_16690	NA
Master_Control_24327	NA
Master_Control_19867	NA
Master_Control_14634	NA
Master_Control_10175	NA
Master_Control_26416	NA
Master_Control_16161	NA
Master_Control_14480	NA
Master_Control_31986	NA
Master_Control_19949	NA
Master_Control_5727	NA
Master_Control_17091	NA
Master_Control_237	NA
Master_Control_28004	NA
Master_Control_31147	NA
Master_Control_11156	NA
Master_Control_19781	NA
Master_Control_19582	NA
Master_Control_5728	NA
Master_Control_15138	NA
Master_Control_28209	NA
Master_Control_13315	NA
Master_Control_3410	NA
Master_Control_28585	NA
Master_Control_10291	NA
Master_Control_26059	NA
Master_Control_6694	NA
Master_Control_32008	NA
Master_Control_26495	NA
Master_Control_23840	NA
Master_Control_28173	NA
Master_Control_6571	NA
Master_Control_14453	NA

Master_Control_15833	NA
Master_Control_7820	NA
Master_Control_4965	NA
Master_Control_14147	NA
Master_Control_14343	NA
Master_Control_29660	NA
Master_Control_1776	NA
Master_Control_29000	NA
Master_Control_30340	NA
Master_Control_32326	NA
Master_Control_24407	NA
Master_Control_9097	NA
Master_Control_19224	NA
Master_Control_3335	NA
Master_Control_15206	NA
Master_Control_13799	NA
Master_Control_32394	NA
Master_Control_9571	NA
Master_Control_18099	NA
Master_Control_12012	NA
Master_Control_14248	NA
Master_Control_18931	NA
Master_Control_23747	NA
Master_Control_24064	NA
Master_Control_22244	NA
Master_Control_4910	NA
Master_Control_27981	NA
Master_Control_11786	NA
Master_Control_30058	NA
Master_Control_5476	NA
Master_Control_4923	NA
Master_Control_16454	NA
Master_Control_18135	NA
Master_Control_11574	NA
Master_Control_10728	NA
Master_Control_31911	NA
Master_Control_17003	NA
Master_Control_8513	NA
Master_Control_23102	NA
Master_Control_16387	NA
Master_Control_3373	NA
Master_Control_7271	NA
Master_Control_10136	NA
Master_Control_11715	NA
Master_Control_656	NA

Master_Control_13110	NA
Master_Control_31338	NA
Master_Control_13563	NA
Master_Control_22593	NA
Master_Control_28702	NA
Master_Control_20488	NA
Master_Control_26334	NA
Master_Control_10840	NA
Master_Control_1192	NA
Master_Control_23574	NA
Master_Control_8283	NA
Master_Control_319	NA
Master_Control_32236	NA
Master_Control_20460	NA
Master_Control_4988	NA
Master_Control_2081	NA
Master_Control_11930	NA
Master_Control_13020	NA
Master_Control_26449	NA
Master_Control_31529	NA
Master_Control_31799	NA
Master_Control_23296	NA
Master_Control_19040	NA
Master_Control_14007	NA
Master_Control_14406	NA
Master_Control_27332	NA
Master_Control_29047	NA
Master_Control_14777	NA
Master_Control_8428	NA
Master_Control_26179	NA
Master_Control_20860	NA
Master_Control_2250	NA
Master_Control_17026	NA
Master_Control_9655	NA
Master_Control_31452	NA
Master_Control_1489	NA
Master_Control_27843	NA
Master_Control_17056	NA
Master_Control_5338	NA
Master_Control_26360	NA
Master_Control_4154	NA
Master_Control_3998	NA
Master_Control_3164	NA
Master_Control_27477	NA
Master_Control_849	NA

Master_Control_9115	NA
Master_Control_11692	NA
Master_Control_12896	NA
Master_Control_2735	NA
Master_Control_13729	NA
Master_Control_19546	NA
Master_Control_20872	NA
Master_Control_314	NA
Master_Control_12141	NA
Master_Control_4371	NA
Master_Control_17272	NA
Master_Control_9466	NA
Master_Control_10270	NA
Master_Control_26488	NA
Master_Control_2579	NA
Master_Control_5088	NA
Master_Control_5964	NA
Master_Control_16731	NA
Master_Control_2576	NA
Master_Control_23240	NA
Master_Control_31641	NA
Master_Control_32212	NA
Master_Control_6162	NA
Master_Control_24540	NA
Master_Control_384	NA
Master_Control_25685	NA
Master_Control_8208	NA
Master_Control_13033	NA
Master_Control_12938	NA
Master_Control_19652	NA
Master_Control_2255	NA
Master_Control_17240	NA
Master_Control_23347	NA
Master_Control_13593	NA
Master_Control_30636	NA
Master_Control_4235	NA
Master_Control_8529	NA
Master_Control_21394	NA
Master_Control_15699	NA
Master_Control_746	NA
Master_Control_25760	NA
Master_Control_26886	NA
Master_Control_14930	NA
Master_Control_15992	NA
Master_Control_25090	NA

Master_Control_23641	NA
Master_Control_19637	NA
Master_Control_12596	NA
Master_Control_30270	NA
Master_Control_14620	NA
Master_Control_8945	NA
Master_Control_298	NA
Master_Control_22876	NA
Master_Control_785	NA
Master_Control_15988	NA
Master_Control_7062	NA
Master_Control_22322	NA
Master_Control_20604	NA
Master_Control_17606	NA
Master_Control_4355	NA
Master_Control_13289	NA
Master_Control_1748	NA
Master_Control_13558	NA
Master_Control_13421	NA
Master_Control_19836	NA
Master_Control_31955	NA
Master_Control_2566	NA
Master_Control_19495	NA
Master_Control_14037	NA
Master_Control_20357	NA
Master_Control_19900	NA
Master_Control_4191	NA
Master_Control_27690	NA
Master_Control_23821	NA
Master_Control_24932	NA
Master_Control_8137	NA
Master_Control_28898	NA
Master_Control_1698	NA
Master_Control_9852	NA
Master_Control_19756	NA
Master_Control_18632	NA
Master_Control_10947	NA
Master_Control_18892	NA
Master_Control_8525	NA
Master_Control_19666	NA
Master_Control_15529	NA
Master_Control_27944	NA
Master_Control_14321	NA
Master_Control_5909	NA
Master_Control_23363	NA

Master_Control_426	NA
Master_Control_29318	NA
Master_Control_22061	NA
Master_Control_13661	NA
Master_Control_22479	NA
Master_Control_7616	NA
Master_Control_15454	NA
Master_Control_2848	NA
Master_Control_25551	NA
Master_Control_16027	NA
Master_Control_18151	NA
Master_Control_29419	NA
Master_Control_28499	NA
Master_Control_5489	NA
Master_Control_2532	NA
Master_Control_14285	NA
Master_Control_1005	NA
Master_Control_27075	NA
Master_Control_31203	NA
Master_Control_29918	NA
Master_Control_12400	NA
Master_Control_7713	NA
Master_Control_31521	NA
Master_Control_11535	NA
Master_Control_26980	NA
Master_Control_30347	NA
Master_Control_11811	NA
Master_Control_28358	NA
Master_Control_17083	NA
Master_Control_15533	NA
Master_Control_7671	NA
Master_Control_5994	NA
Master_Control_16347	NA
Master_Control_8697	NA
Master_Control_24041	NA
Master_Control_2624	NA
Master_Control_8711	NA
Master_Control_21236	NA
Master_Control_32440	NA
Master_Control_6502	NA
Master_Control_11154	NA
Master_Control_25604	NA
Master_Control_7546	NA
Master_Control_24553	NA
Master_Control_26983	NA

Master_Control_9421	NA
Master_Control_26329	NA
Master_Control_28836	NA
Master_Control_13637	NA
Master_Control_3567	NA
Master_Control_21874	NA
Master_Control_6248	NA
Master_Control_18565	NA
Master_Control_16761	NA
Master_Control_3059	NA
Master_Control_30166	NA
Master_Control_6690	NA
Master_Control_14200	NA
Master_Control_24842	NA
Master_Control_19791	NA
Master_Control_6924	NA
Master_Control_23037	NA
Master_Control_6036	NA
Master_Control_5666	NA
Master_Control_18833	NA
Master_Control_22189	NA
Master_Control_1569	NA
Master_Control_31409	NA
Master_Control_28138	NA
Master_Control_13290	NA
Master_Control_2707	NA
Master_Control_14897	NA
Master_Control_2486	NA
Master_Control_262	NA
Master_Control_11632	NA
Master_Control_2753	NA
Master_Control_19089	NA
Master_Control_31923	NA
Master_Control_2808	NA
Master_Control_13466	NA
Master_Control_24507	NA
Master_Control_1342	NA
Master_Control_9162	NA
Master_Control_22325	NA
Master_Control_18100	NA
Master_Control_23600	NA
Master_Control_21487	NA
Master_Control_9270	NA
Master_Control_21864	NA
Master_Control_24271	NA

Master_Control_91	NA
Master_Control_25355	NA
Master_Control_7102	NA
Master_Control_18182	NA
Master_Control_6019	NA
Master_Control_19087	NA
Master_Control_30494	NA
Master_Control_8551	NA
Master_Control_5200	NA
Master_Control_20718	NA
Master_Control_14115	NA
Master_Control_20375	NA
Master_Control_12646	NA
Master_Control_30966	NA
Master_Control_5581	NA
Master_Control_16059	NA
Master_Control_21306	NA
Master_Control_14701	NA
Master_Control_6543	NA
Master_Control_8162	NA
Master_Control_29702	NA
Master_Control_25833	NA
Master_Control_31228	NA
Master_Control_20058	NA
Master_Control_1617	NA
Master_Control_11465	NA
Master_Control_19143	NA
Master_Control_32180	NA
Master_Control_25575	NA
Master_Control_25503	NA

Table S8. Genes commonly up-regulated.

Unigene_ID	Protein_Names
Master_Control_11791	Meiotic recombination protein DMC1
Master_Control_1480	Meiotic recombination protein DMC1
Master_Control_17708	Meiotic recombination protein DMC1
Master_Control_26098	Meiotic recombination protein DMC1
Master_Control_6445	Meiotic recombination protein DMC1
Master_Control_1354	NADH-dependent glutamate synthase 1 isoform 4
Master_Control_142258	NADH-dependent glutamate synthase 1 isoform 4
Master_Control_189921	NADH-dependent glutamate synthase 1 isoform 4
Master_Control_21062	NADH-dependent glutamate synthase 1 isoform 4
Master_Control_27365	NADH-dependent glutamate synthase 1 isoform 4
Master_Control_9911	NADH-dependent glutamate synthase 1 isoform 4
Master_Control_87314	Non-specific serine/threonine protein kinase (EC 2.7.11.1)

Master_Control_192118	Pto-like serine/threonine kinase
Master_Control_2284	Putative leucine-rich repeat receptor-like serine/threonine-protein kinase
Master_Control_17763	Serine/threonine-protein kinase (EC 2.7.11.1)
Master_Control_204216	Serine-threonine protein kinase, plant-type, putative
Master_Control_10675	Photosystem Q(B) protein (EC 1.10.3.9) (32 kDa thylakoid membrane protein) (Photosystem II protein D1)
Master_Control_13345	Photosystem Q(B) protein (EC 1.10.3.9) (32 kDa thylakoid membrane protein) (Photosystem II protein D1)
Master_Control_13346	Photosystem Q(B) protein (EC 1.10.3.9) (32 kDa thylakoid membrane protein) (Photosystem II protein D1)
Master_Control_13412	Photosystem Q(B) protein (EC 1.10.3.9) (32 kDa thylakoid membrane protein) (Photosystem II protein D1)
Master_Control_15024	Photosystem Q(B) protein (EC 1.10.3.9) (32 kDa thylakoid membrane protein) (Photosystem II protein D1)
Master_Control_16322	Photosystem Q(B) protein (EC 1.10.3.9) (32 kDa thylakoid membrane protein) (Photosystem II protein D1)
Master_Control_23793	Photosystem Q(B) protein (EC 1.10.3.9) (32 kDa thylakoid membrane protein) (Photosystem II protein D1)
Master_Control_24489	Photosystem Q(B) protein (EC 1.10.3.9) (32 kDa thylakoid membrane protein) (Photosystem II protein D1)
Master_Control_3688	Photosystem Q(B) protein (EC 1.10.3.9) (32 kDa thylakoid membrane protein) (Photosystem II protein D1)
Master_Control_6331	Photosystem Q(B) protein (EC 1.10.3.9) (32 kDa thylakoid membrane protein) (Photosystem II protein D1)
Master_Control_6880	Photosystem Q(B) protein (EC 1.10.3.9) (32 kDa thylakoid membrane protein) (Photosystem II protein D1)
Master_Control_7115	Photosystem Q(B) protein (EC 1.10.3.9) (32 kDa thylakoid membrane protein) (Photosystem II protein D1)
Master_Control_8068	Photosystem Q(B) protein (EC 1.10.3.9) (32 kDa thylakoid membrane protein) (Photosystem II protein D1)
Master_Control_9524	Photosystem Q(B) protein (EC 1.10.3.9) (32 kDa thylakoid membrane protein) (Photosystem II protein D1)
Master_Control_113386	(8,11)-linoleoyl desaturase (Fragment)
Master_Control_178458	1-aminocyclopropapne-1-carboxylic acid oxidase
Master_Control_135774	3-isopropylmalate dehydrogenase (Fragment)
Master_Control_46457	Albumin-2 protein (Hemopexin; D-tyrosyl-tRNA(Tyr) deacylase)
Master_Control_176432	Alcohol dehydrogenases homologous
Master_Control_135821	Anthocyanin malonyltransferase homolog
Master_Control_136900	AP-3 complex subunit beta
Master_Control_136901	AP-3 complex subunit beta
Master_Control_52867	AP-3 complex subunit beta
Master_Control_89848	ARM repeat protein
Master_Control_181591	ATP synthase subunit b, chloroplastic (ATP synthase F(0) sector subunit b) (ATPase subunit I)
Master_Control_93043	ATP synthase subunit beta, chloroplastic (EC 3.6.3.14) (ATP synthase F1 sector subunit beta) (F-ATPase subunit beta)
Master_Control_82518	Beta-1,3-glucanase
Master_Control_123816	BnaA06g13620D protein
Master_Control_174056	BnaA10g12960D protein
Master_Control_127057	BnaC09g29270D protein
Master_Control_129888	Broad substrate reductase/dehydrogenase
Master_Control_129237	Cell wall-associated hydrolase (Fragment)
Master_Control_93682	Cell wall-associated hydrolase (Fragment)
Master_Control_187706	Cyclophilin (Fragment)
Master_Control_186972	Cytochrome b559 subunit alpha (PSII reaction center subunit V)

Master_Control_79935	DNA (cytosine-5)-methyltransferase (EC 2.1.1.37)
Master_Control_126963	DNA polymerase (EC 2.7.7.7)
Master_Control_185933	DNA polymerase (EC 2.7.7.7)
Master_Control_159264	DNA topoisomerase 6 subunit A (EC 5.99.1.3)
Master_Control_95306	DNA-directed RNA polymerase subunit beta'' (EC 2.7.7.6) (PEP) (Plastid-encoded RNA polymerase subunit beta'')
Master_Control_188886	Endoplasmatic reticulum retrieval protein 1B isoform 1
Master_Control_189811	ERECTA-like 1
Master_Control_174015	FAD2-8 protein (EC 1.14.19.6)
Master_Control_92902	FAD2-8 protein (EC 1.14.19.6)
Master_Control_37933	Glycerol-3-phosphate dehydrogenase [NAD()] (EC 1.1.1.8)
Master_Control_177038	HVA22-like protein J, putative
Master_Control_26850	Hydroxycinnamoyl-CoA:quinate hydroxycinnamoyltransferase
Master_Control_125767	Late elongated hypocotyl-like
Master_Control_125769	Late elongated hypocotyl-like
Master_Control_125770	Late elongated hypocotyl-like
Master_Control_133587	Lon protease homolog, mitochondrial (EC 3.4.21.-)
Master_Control_180335	Maturase K (Intron maturase)
Master_Control_17725	Midasin
Master_Control_93407	Midasin
Master_Control_51537	MUTS
Master_Control_94864	NADH-ubiquinone oxidoreductase chain 4 (EC 1.6.5.3)
Master_Control_105730	NADPH--cytochrome P450 reductase (EC 1.6.2.4)
Master_Control_128835	Non-LTR retroelement reverse transcriptase-like protein
Master_Control_188488	Non-LTR retroelement reverse transcriptase-like protein
Master_Control_15814	Pectin lyase-like superfamily protein isoform 2
Master_Control_90659	Phosphatase 2c, putative isoform 2
Master_Control_97590	Photosystem I P700 chlorophyll a apoprotein A1 (EC 1.97.1.12) (PSI-A) (PsaA)
Master_Control_75599	Polygalacturonase-inhibiting protein 4
Master_Control_39115	Polyphenol oxidase (EC 1.10.3.2)
Master_Control_161290	Predicted protein
Master_Control_179628	Putative ethylene receptor ETR2
Master_Control_115975	Putative expansin (Fragment)
Master_Control_101176	Replication factor-A carboxy-terminal domain protein
Master_Control_179833	S-acyltransferase (EC 2.3.1.225) (Palmitoyltransferase)
Master_Control_187747	Salt overly sensitive
Master_Control_17852	Structural maintenance of chromosomes 6A, putative isoform 1
Master_Control_182811	Subunit H2, putative
Master_Control_57072	Tic20, putative
Master_Control_18101	Trehalose 6-phosphate phosphatase (EC 3.1.3.12)
Master_Control_126262	Zn-dependent exopeptidases superfamily protein
Master_Control_134676	Coffea canephora DH200=94 genomic scaffold, scaffold_135
Master_Control_175677	Coffea canephora DH200=94 genomic scaffold, scaffold_19
Master_Control_187861	Coffea canephora DH200=94 genomic scaffold, scaffold_19
Master_Control_191100	Coffea canephora DH200=94 genomic scaffold, scaffold_21
Master_Control_188865	Coffea canephora DH200=94 genomic scaffold, scaffold_3
Master_Control_37864	Coffea canephora DH200=94 genomic scaffold, scaffold_45
Master_Control_83038	Coffea canephora DH200=94 genomic scaffold, scaffold_5
Master_Control_150502	Coffea canephora DH200=94 genomic scaffold, scaffold_6
Master_Control_40350	Coffea canephora DH200=94 genomic scaffold, scaffold_68
Master_Control_73748	Coffea canephora DH200=94 genomic scaffold, scaffold_7
Master_Control_163463	Coffea canephora DH200=94 genomic scaffold, scaffold_92

Master_Control_98746	Coronatine-insensitive 1 (Uncharacterized protein)
Master_Control_10642	NA
Master_Control_10662	NA
Master_Control_107496	NA
Master_Control_113204	NA
Master_Control_114132	NA
Master_Control_114678	NA
Master_Control_115664	NA
Master_Control_117252	NA
Master_Control_117888	NA
Master_Control_119331	NA
Master_Control_12048	NA
Master_Control_12280	NA
Master_Control_123244	NA
Master_Control_126783	NA
Master_Control_132268	NA
Master_Control_134234	NA
Master_Control_13555	NA
Master_Control_136768	NA
Master_Control_139216	NA
Master_Control_13946	NA
Master_Control_139756	NA
Master_Control_139757	NA
Master_Control_140192	NA
Master_Control_141598	NA
Master_Control_14385	NA
Master_Control_1445	NA
Master_Control_148095	NA
Master_Control_152253	NA
Master_Control_15276	NA
Master_Control_157982	NA
Master_Control_158343	NA
Master_Control_161077	NA
Master_Control_162223	NA
Master_Control_162691	NA
Master_Control_163471	NA
Master_Control_163482	NA
Master_Control_166190	NA
Master_Control_166598	NA
Master_Control_166613	NA
Master_Control_167386	NA
Master_Control_167406	NA
Master_Control_169207	NA
Master_Control_170567	NA
Master_Control_171510	NA
Master_Control_172404	NA
Master_Control_175290	NA
Master_Control_17871	NA
Master_Control_185249	NA
Master_Control_1890	NA
Master_Control_190140	NA
Master_Control_190255	NA

Master_Control_191774	NA
Master_Control_195127	NA
Master_Control_196208	NA
Master_Control_20198	NA
Master_Control_24015	NA
Master_Control_2411	NA
Master_Control_24184	NA
Master_Control_2670	NA
Master_Control_27508	NA
Master_Control_27566	NA
Master_Control_27646	NA
Master_Control_29971	NA
Master_Control_30064	NA
Master_Control_30829	NA
Master_Control_31484	NA
Master_Control_35404	NA
Master_Control_35803	NA
Master_Control_36024	NA
Master_Control_37680	NA
Master_Control_38485	NA
Master_Control_412	NA
Master_Control_41670	NA
Master_Control_42542	NA
Master_Control_44656	NA
Master_Control_4636	NA
Master_Control_47070	NA
Master_Control_51541	NA
Master_Control_57560	NA
Master_Control_65992	NA
Master_Control_67240	NA
Master_Control_73116	NA
Master_Control_75392	NA
Master_Control_76499	NA
Master_Control_78584	NA
Master_Control_79416	NA
Master_Control_80032	NA
Master_Control_85343	NA
Master_Control_86430	NA
Master_Control_8718	NA
Master_Control_88571	NA
Master_Control_91415	NA
Master_Control_9185	NA
Master_Control_93	NA
Master_Control_93352	NA
Master_Control_96220	NA
Master_Control_97247	NA
Master_Control_97253	NA
Master_Control_97260	NA
Master_Control_98011	NA
Master_Control_9811	NA
Master_Control_100590	Putative uncharacterized protein
Master_Control_125021	Putative uncharacterized protein

Master_Control_125114	Putative uncharacterized protein
Master_Control_127672	Putative uncharacterized protein
Master_Control_135075	Putative uncharacterized protein
Master_Control_135826	Putative uncharacterized protein
Master_Control_135827	Putative uncharacterized protein
Master_Control_143170	Putative uncharacterized protein
Master_Control_164084	Putative uncharacterized protein
Master_Control_178975	Putative uncharacterized protein
Master_Control_182232	Putative uncharacterized protein
Master_Control_182233	Putative uncharacterized protein
Master_Control_185896	Putative uncharacterized protein
Master_Control_186537	Putative uncharacterized protein
Master_Control_186813	Putative uncharacterized protein
Master_Control_192094	Putative uncharacterized protein
Master_Control_38726	Putative uncharacterized protein
Master_Control_39288	Putative uncharacterized protein
Master_Control_40066	Putative uncharacterized protein
Master_Control_42428	Putative uncharacterized protein
Master_Control_53730	Putative uncharacterized protein
Master_Control_77554	Putative uncharacterized protein
Master_Control_88119	Putative uncharacterized protein
Master_Control_93995	Putative uncharacterized protein
Master_Control_9967	Putative uncharacterized protein
Master_Control_50521	Putative uncharacterized protein (Fragment)
Master_Control_3208	Putative uncharacterized protein Sb04g017405 (Fragment)
Master_Control_11181	Uncharacterized protein
Master_Control_11280	Uncharacterized protein
Master_Control_113494	Uncharacterized protein
Master_Control_115820	Uncharacterized protein
Master_Control_117097	Uncharacterized protein
Master_Control_119720	Uncharacterized protein
Master_Control_121515	Uncharacterized protein
Master_Control_122443	Uncharacterized protein
Master_Control_122771	Uncharacterized protein
Master_Control_125028	Uncharacterized protein
Master_Control_125721	Uncharacterized protein
Master_Control_128482	Uncharacterized protein
Master_Control_131785	Uncharacterized protein
Master_Control_133767	Uncharacterized protein
Master_Control_133951	Uncharacterized protein
Master_Control_134172	Uncharacterized protein
Master_Control_135575	Uncharacterized protein
Master_Control_137991	Uncharacterized protein
Master_Control_141956	Uncharacterized protein
Master_Control_143939	Uncharacterized protein
Master_Control_14954	Uncharacterized protein
Master_Control_163848	Uncharacterized protein
Master_Control_164893	Uncharacterized protein
Master_Control_165869	Uncharacterized protein
Master_Control_166156	Uncharacterized protein
Master_Control_166540	Uncharacterized protein
Master_Control_168127	Uncharacterized protein

Master_Control_169338	Uncharacterized protein
Master_Control_171397	Uncharacterized protein
Master_Control_172635	Uncharacterized protein
Master_Control_1743	Uncharacterized protein
Master_Control_176395	Uncharacterized protein
Master_Control_177647	Uncharacterized protein
Master_Control_182500	Uncharacterized protein
Master_Control_183567	Uncharacterized protein
Master_Control_189966	Uncharacterized protein
Master_Control_192244	Uncharacterized protein
Master_Control_201253	Uncharacterized protein
Master_Control_21856	Uncharacterized protein
Master_Control_22197	Uncharacterized protein
Master_Control_24402	Uncharacterized protein
Master_Control_25094	Uncharacterized protein
Master_Control_28646	Uncharacterized protein
Master_Control_34017	Uncharacterized protein
Master_Control_34806	Uncharacterized protein
Master_Control_3523	Uncharacterized protein
Master_Control_37628	Uncharacterized protein
Master_Control_3831	Uncharacterized protein
Master_Control_38757	Uncharacterized protein
Master_Control_42698	Uncharacterized protein
Master_Control_44715	Uncharacterized protein
Master_Control_47166	Uncharacterized protein
Master_Control_50537	Uncharacterized protein
Master_Control_56461	Uncharacterized protein
Master_Control_58640	Uncharacterized protein
Master_Control_6635	Uncharacterized protein
Master_Control_7396	Uncharacterized protein
Master_Control_75573	Uncharacterized protein
Master_Control_76560	Uncharacterized protein
Master_Control_77280	Uncharacterized protein
Master_Control_78428	Uncharacterized protein
Master_Control_845	Uncharacterized protein
Master_Control_85307	Uncharacterized protein
Master_Control_87249	Uncharacterized protein
Master_Control_87646	Uncharacterized protein
Master_Control_88363	Uncharacterized protein
Master_Control_91858	Uncharacterized protein
Master_Control_9802	Uncharacterized protein
Master_Control_128631	Uncharacterized protein (Fragment)
Master_Control_204336	Uncharacterized protein (Fragment)
Master_Control_24785	Uncharacterized protein (Fragment)
Master_Control_46876	Uncharacterized protein (Fragment)
Master_Control_94296	Uncharacterized protein (Fragment)

Table S9. transcripts commonly down-regulated.

Unigene_ID	Protein_Names
Master_Control_31050	Sucrose transporter
Master_Control_17023	Sucrose transporter 2

Master_Control_19	Sucrose transporter 2
Master_Control_6750	Sucrose transporter 2
Master_Control_7792	Sucrose transporter 2
Master_Control_132201	N-acetylglucosaminyltransferase I (EC 2.4.1.101)
Master_Control_15718	N-acetylglucosaminyltransferase I (EC 2.4.1.101)
Master_Control_19976	N-acetylglucosaminyltransferase I (EC 2.4.1.101)
Master_Control_2180	N-acetylglucosaminyltransferase I (EC 2.4.1.101)
Master_Control_22600	N-acetylglucosaminyltransferase I (EC 2.4.1.101)
Master_Control_23401	N-acetylglucosaminyltransferase I (EC 2.4.1.101)
Master_Control_25223	N-acetylglucosaminyltransferase I (EC 2.4.1.101)
Master_Control_25664	N-acetylglucosaminyltransferase I (EC 2.4.1.101)
Master_Control_26767	N-acetylglucosaminyltransferase I (EC 2.4.1.101)
Master_Control_27272	N-acetylglucosaminyltransferase I (EC 2.4.1.101)
Master_Control_29604	N-acetylglucosaminyltransferase I (EC 2.4.1.101)
Master_Control_31267	N-acetylglucosaminyltransferase I (EC 2.4.1.101)
Master_Control_31839	N-acetylglucosaminyltransferase I (EC 2.4.1.101)
Master_Control_32368	N-acetylglucosaminyltransferase I (EC 2.4.1.101)
Master_Control_4631	N-acetylglucosaminyltransferase I (EC 2.4.1.101)
Master_Control_4848	N-acetylglucosaminyltransferase I (EC 2.4.1.101)
Master_Control_15571	CC-NBS-LRR
Master_Control_3372	CC-NBS-LRR resistance-like protein RGC203
Master_Control_6510	CC-NBS-LRR resistance-like protein RGC203
Master_Control_9144	CC-NBS-LRR resistance-like protein RGC203
Master_Control_9564	NBS protein (Fragment)
Master_Control_11570	NBS-LRR protein
Master_Control_8003	NBS-LRR protein
Master_Control_192907	NBS-LRR resistance-like protein RGC260
Master_Control_28378	NBS-LRR resistance-like protein RGC260
Master_Control_5256	NBS-LRR resistance-like protein RGC260
Master_Control_11773	TIR-NBS-LRR resistance-like protein RGC151
Master_Control_17300	TIR-NBS-LRR resistance-like protein RGC151
Master_Control_18084	TIR-NBS-LRR resistance-like protein RGC151
Master_Control_18284	TIR-NBS-LRR resistance-like protein RGC151
Master_Control_18305	TIR-NBS-LRR resistance-like protein RGC151
Master_Control_19314	TIR-NBS-LRR resistance-like protein RGC151
Master_Control_2570	TIR-NBS-LRR resistance-like protein RGC151
Master_Control_30774	TIR-NBS-LRR resistance-like protein RGC151
Master_Control_31700	TIR-NBS-LRR resistance-like protein RGC151
Master_Control_12119	ATP-dependent DNA helicase PIF1
Master_Control_13322	ATP-dependent DNA helicase PIF1
Master_Control_24937	ATP-dependent DNA helicase PIF1
Master_Control_1178	17.6 kDa heat-shock protein
Master_Control_565	17.6 kDa heat-shock protein
Master_Control_11130	17.67 kDa heat-shock protein
Master_Control_21112	17.67 kDa heat-shock protein
Master_Control_323	17.67 kDa heat-shock protein
Master_Control_31836	17.7 KD class I small heat-shock protein
Master_Control_19258	18.6 kDa heat-shock protein
Master_Control_10617	Heat shock protein 60 isoform 1
Master_Control_21043	Heat shock protein 60 isoform 1
Master_Control_28269	Heat shock protein 60 isoform 1
Master_Control_5254	Heat shock protein 60 isoform 1

Master_Control_59350	Heat shock protein 60 isoform 1
Master_Control_4719	1,2-beta-fructan 1F-fructosyltransferase (EC 2.4.1.100)
Master_Control_18256	2-alkenal reductase
Master_Control_18661	2-alkenal reductase
Master_Control_131474	50S ribosomal protein L16, chloroplastic
Master_Control_24618	60I2G03 protein
Master_Control_20412	Aldose 1-epimerase (EC 5.1.3.3) (Galactose mutarotase)
Master_Control_178486	Alliinase family protein
Master_Control_280	Alpha/beta hydrolase, putative
Master_Control_49306	Aquaporin SIP2.1 family protein
Master_Control_27118	At3g47680 (DNA binding protein) (Putative uncharacterized protein T23J7.10)
Master_Control_88374	ATP synthase protein I-related protein (Fragment)
Master_Control_3311	Beta-1,3-N-acetylglucosaminyltransferase family protein
Master_Control_46927	Beta-caryophyllene synthase (EC 4.2.3.57)
Master_Control_30628	Beta-fructofuranosidase, putative
Master_Control_52336	Bifunctional dihydroflavonol 4-reductase/flavanone 4-reductase isoform 1
Master_Control_127042	BnaA07g15650D protein
Master_Control_194893	BnaC02g21710D protein
Master_Control_18899	Casein kinase I isoform delta-like protein
Master_Control_1925	Cellulose synthase 3B (Fragment)
Master_Control_3234	Centrin2 isoform 1
Master_Control_59353	Chaperonin-60kD, ch60, putative
Master_Control_30933	Chloroplast RNA-binding protein 33
Master_Control_30453	ChNRRa
Master_Control_40235	ChNRRa
Master_Control_19003	Cinnamoyl-CoA reductase, putative (EC 1.1.1.219)
Master_Control_20960	Cinnamoyl-CoA reductase, putative (EC 1.1.1.219)
Master_Control_16709	Cold-shock DNA-binding family protein
Master_Control_27154	Common plant regulatory factor 6
Master_Control_24789	Condensin complex subunit 2
Master_Control_11884	Cyclin-dependent kinase inhibitor 7
Master_Control_8744	Cytochrome P450 superfamily protein
Master_Control_137837	D-cysteine desulfhydrase (Uncharacterized protein)
Master_Control_15422	Dead box ATP-dependent RNA helicase, putative
Master_Control_23836	Diphthamide biosynthesis protein, putative
Master_Control_20024	D-isomer specific 2-hydroxyacid dehydrogenase family protein
Master_Control_179641	DnaJ homolog subfamily C member 3 homolog
Master_Control_10519	DNAse I-like superfamily protein
Master_Control_31016	Double bond reductase
Master_Control_53015	DREB6-like protein
Master_Control_16448	D-tyrosyl-tRNA(Tyr) deacylase (EC 3.1.-.-)
Master_Control_170839	Dual specificity protein phosphatase (DsPTP1) family protein isoform 1
Master_Control_30876	Early light-induced protein, chloroplastic (ELIP)
Master_Control_12453	Endo-1,4-beta-xylanase C, putative (EC 3.2.1.8)
Master_Control_27232	Endoglucanase (EC 3.2.1.4)
Master_Control_29751	Extra-large GTP-binding protein 3
Master_Control_15684	FAR1; Zinc finger, SWIM-type
Master_Control_28744	F-box and associated interaction domains-containing-like protein
Master_Control_23763	F-box family protein
Master_Control_18034	F-box family protein, putative isoform 4
Master_Control_9210	Ferric reduction oxidase 7 isoform 2

Master_Control_155077	Flower development transporter PI
Master_Control_183553	Fructan:fructan 1-fructosyltransferase (EC 2.4.1.100)
Master_Control_22940	Gag-Pol
Master_Control_192660	Gag-pol polymerase (Fragment)
Master_Control_1154	Glucose acyltransferase
Master_Control_136109	GLUCOSE INSENSITIVE 1 family protein
Master_Control_115066	Glucose-6-phosphate 1-dehydrogenase (EC 1.1.1.49)
Master_Control_75441	Glucose-6-phosphate 1-dehydrogenase (EC 1.1.1.49) (Fragment)
Master_Control_182766	Glucosyltransferase (EC 2.4.1.-)
Master_Control_198100	Glutamate dehydrogenase (Fragment)
Master_Control_7518	Glutamyl-tRNA(Gln) amidotransferase subunit A, chloroplastic/mitochondrial (Glu-AdT subunit A) (EC 6.3.5.7)
Master_Control_30563	Glutathione S-transferase zeta class (EC 2.5.1.18)
Master_Control_7293	Glycolate oxidase
Master_Control_14735	Golgin candidate 6 isoform 2
Master_Control_122656	Hexokinase
Master_Control_173496	Hexokinase
Master_Control_42438	Histone deacetylase (EC 3.5.1.98)
Master_Control_10904	Hydrogen peroxide induced protein 1
Master_Control_16999	Hydroxycinnamoyl-CoA transferase 2
Master_Control_40922	Lactosylceramide 4-alpha-galactosyltransferase, putative (EC 2.4.1.228)
Master_Control_50773	Luminal binding protein
Master_Control_59355	Malic enzyme
Master_Control_21312	MIXTA-like 2
Master_Control_8483	Mixta-like 2.3 protein (Fragment)
Master_Control_184257	MYB
Master_Control_25079	MYB1 protein
Master_Control_164739	NAC-domain protein
Master_Control_4144	NAD(P)-binding rossmann-fold protein
Master_Control_15074	NAD-dependent protein deacetylase (EC 3.5.1.-) (Regulatory protein SIR2 homolog)
Master_Control_11795	Natural resistance-associated macrophage protein, putative
Master_Control_45633	Nucleic acid binding protein, putative
Master_Control_12993	Nucleic acid-binding protein, putative
Master_Control_20936	Orf124 protein
Master_Control_5060	Orf124 protein
Master_Control_162324	Os01g0879800 protein
Master_Control_1159	Os04g0283700 protein (Fragment)
Master_Control_2325	OSIGBa0134J07.9 protein
Master_Control_137287	OSJNBa0074B10.9 protein
Master_Control_15444	OSJNBa0074B10.9 protein
Master_Control_20847	OSJNBa0074B10.9 protein
Master_Control_27973	OSJNBa0074B10.9 protein
Master_Control_29266	OSJNBa0074B10.9 protein
Master_Control_6246	Pectate lyase (EC 4.2.2.2)
Master_Control_9757	Pectate lyase (EC 4.2.2.2)
Master_Control_119264	Pectinesterase (EC 3.1.1.11)
Master_Control_26560	Pentatricopeptide repeat-containing protein, putative
Master_Control_166085	Peptidyl-prolyl cis-trans isomerase
Master_Control_23175	Peptidyl-prolyl cis-trans isomerase
Master_Control_23032	Peroxisomal membrane carrier protein (Uncharacterized protein)
Master_Control_2215	Phosphate transporter 1
Master_Control_24773	Polyprotein

Master_Control_21071	Protein argonaute
Master_Control_23860	Protein phosphatase 2c, putative (EC 3.1.3.16)
Master_Control_21374	P-type ATPase
Master_Control_21809	P-type ATPase
Master_Control_122654	Purple acid phosphatase (EC 3.1.3.2)
Master_Control_9641	Putative arogenate dehydratase
Master_Control_13360	Putative BPI/LBP family protein
Master_Control_29841	Putative importin-7-like protein
Master_Control_26590	Putative inactive purple acid phosphatase 27
Master_Control_1524	Putative polyprotein
Master_Control_12590	Putative reverse transcriptase
Master_Control_29149	Ribosomal protein L13
Master_Control_15989	RNA binding protein, putative
Master_Control_10234	RNI-like superfamily protein
Master_Control_23777	RNI-like superfamily protein
Master_Control_13896	RuBisCO large subunit-binding protein subunit alpha
Master_Control_18966	Rubisco methyltransferase family protein (Uncharacterized protein)
Master_Control_30975	Rubisco methyltransferase family protein (Uncharacterized protein)
Master_Control_129946	Senescence-related gene 3 isoform 1
Master_Control_23152	Serine/threonine-protein phosphatase (EC 3.1.3.16)
Master_Control_475	Sodium:hydrogen antiporter 1 (Fragment)
Master_Control_21884	Sodium-bile acid cotransporter, putative
Master_Control_2828	Sodium-bile acid cotransporter, putative
Master_Control_8390	Sterile nodes
Master_Control_6007	Systemin receptor SR160, putative (EC 1.3.1.74)
Master_Control_27902	T28P6.8 protein
Master_Control_15984	TLD-domain containing nucleolar protein isoform 2
Master_Control_19142	TMV resistance protein N, putative
Master_Control_177959	Transcription initiation factor IIE subunit beta
Master_Control_23275	Transducin/WD40 repeat-like superfamily protein
Master_Control_25343	Transducin/WD40 repeat-like superfamily protein
Master_Control_2956	Transducin/WD40 repeat-like superfamily protein
Master_Control_22031	Translation initiation factor IF-3
Master_Control_25808	Translation initiation factor IF-3
Master_Control_3838	Transmembrane Fragile-X-F-associated protein isoform 2
Master_Control_163996	Transmembrane protein, putative
Master_Control_23216	Ubiquitin-protein ligase, putative
Master_Control_46260	U-box domain-containing protein 26
Master_Control_21625	UDP-GlcNac-dolichyl-phosphate N-acetylglucosaminephosphotransferase (EC 2.7.8.15)
Master_Control_51074	UDP-glycosyltransferase 73A17
Master_Control_189137	UDP-glycosyltransferase 73C3
Master_Control_21420	VDAC3.1 family protein
Master_Control_123078	WRKY1 transcription factor
Master_Control_27243	WRKY1 transcription factor
Master_Control_30487	Zinc knuckle family protein, putative isoform 2
Master_Control_8442	ZIP family metal transporter
Master_Control_26877	Coffea canephora DH200=94 genomic scaffold, scaffold_105
Master_Control_2001	Coffea canephora DH200=94 genomic scaffold, scaffold_11
Master_Control_22065	Coffea canephora DH200=94 genomic scaffold, scaffold_11624
Master_Control_76905	Coffea canephora DH200=94 genomic scaffold, scaffold_144
Master_Control_7699	Coffea canephora DH200=94 genomic scaffold, scaffold_15

Master_Control_29253	Coffea canephora DH200=94 genomic scaffold, scaffold_19
Master_Control_3405	Coffea canephora DH200=94 genomic scaffold, scaffold_19
Master_Control_11995	Coffea canephora DH200=94 genomic scaffold, scaffold_196
Master_Control_10169	Coffea canephora DH200=94 genomic scaffold, scaffold_2
Master_Control_123186	Coffea canephora DH200=94 genomic scaffold, scaffold_2
Master_Control_28363	Coffea canephora DH200=94 genomic scaffold, scaffold_2
Master_Control_116953	Coffea canephora DH200=94 genomic scaffold, scaffold_22
Master_Control_30710	Coffea canephora DH200=94 genomic scaffold, scaffold_22
Master_Control_7882	Coffea canephora DH200=94 genomic scaffold, scaffold_22
Master_Control_56009	Coffea canephora DH200=94 genomic scaffold, scaffold_23
Master_Control_164738	Coffea canephora DH200=94 genomic scaffold, scaffold_24
Master_Control_5814	Coffea canephora DH200=94 genomic scaffold, scaffold_256
Master_Control_28819	Coffea canephora DH200=94 genomic scaffold, scaffold_3
Master_Control_13067	Coffea canephora DH200=94 genomic scaffold, scaffold_30
Master_Control_679	Coffea canephora DH200=94 genomic scaffold, scaffold_30
Master_Control_40902	Coffea canephora DH200=94 genomic scaffold, scaffold_31
Master_Control_21353	Coffea canephora DH200=94 genomic scaffold, scaffold_34
Master_Control_10625	Coffea canephora DH200=94 genomic scaffold, scaffold_35
Master_Control_160688	Coffea canephora DH200=94 genomic scaffold, scaffold_35
Master_Control_176004	Coffea canephora DH200=94 genomic scaffold, scaffold_36
Master_Control_21716	Coffea canephora DH200=94 genomic scaffold, scaffold_36
Master_Control_2751	Coffea canephora DH200=94 genomic scaffold, scaffold_36
Master_Control_24267	Coffea canephora DH200=94 genomic scaffold, scaffold_38
Master_Control_10056	Coffea canephora DH200=94 genomic scaffold, scaffold_4
Master_Control_1211	Coffea canephora DH200=94 genomic scaffold, scaffold_4
Master_Control_1590	Coffea canephora DH200=94 genomic scaffold, scaffold_4
Master_Control_18286	Coffea canephora DH200=94 genomic scaffold, scaffold_4
Master_Control_2163	Coffea canephora DH200=94 genomic scaffold, scaffold_4
Master_Control_26280	Coffea canephora DH200=94 genomic scaffold, scaffold_4
Master_Control_24124	Coffea canephora DH200=94 genomic scaffold, scaffold_40
Master_Control_17877	Coffea canephora DH200=94 genomic scaffold, scaffold_41
Master_Control_2608	Coffea canephora DH200=94 genomic scaffold, scaffold_41
Master_Control_128586	Coffea canephora DH200=94 genomic scaffold, scaffold_45
Master_Control_128587	Coffea canephora DH200=94 genomic scaffold, scaffold_45
Master_Control_27982	Coffea canephora DH200=94 genomic scaffold, scaffold_45
Master_Control_52984	Coffea canephora DH200=94 genomic scaffold, scaffold_45
Master_Control_21757	Coffea canephora DH200=94 genomic scaffold, scaffold_5
Master_Control_47462	Coffea canephora DH200=94 genomic scaffold, scaffold_50
Master_Control_11161	Coffea canephora DH200=94 genomic scaffold, scaffold_53
Master_Control_180825	Coffea canephora DH200=94 genomic scaffold, scaffold_6
Master_Control_27925	Coffea canephora DH200=94 genomic scaffold, scaffold_62
Master_Control_31000	Coffea canephora DH200=94 genomic scaffold, scaffold_67
Master_Control_29635	Coffea canephora DH200=94 genomic scaffold, scaffold_75
Master_Control_28942	Coffea canephora DH200=94 genomic scaffold, scaffold_76
Master_Control_11772	Coffea canephora DH200=94 genomic scaffold, scaffold_763
Master_Control_2594	Coffea canephora DH200=94 genomic scaffold, scaffold_8
Master_Control_29076	Coffea canephora DH200=94 genomic scaffold, scaffold_8
Master_Control_26493	Coffea canephora DH200=94 genomic scaffold, scaffold_803
Master_Control_32308	Coffea canephora DH200=94 genomic scaffold, scaffold_87
Master_Control_21706	Coffea canephora DH200=94 genomic scaffold, scaffold_870
Master_Control_22603	Coffea canephora DH200=94 genomic scaffold, scaffold_870
Master_Control_29448	Coffea canephora DH200=94 genomic scaffold, scaffold_870

Master_Control_15402	Coffea canephora DH200=94 genomic scaffold, scaffold_9
Master_Control_2811	Coffea canephora DH200=94 genomic scaffold, scaffold_9
Master_Control_8930	Coffea canephora DH200=94 genomic scaffold, scaffold_9
Master_Control_5436	Coffea canephora DH200=94 genomic scaffold, scaffold_91
Master_Control_125013	Coffea canephora DH200=94 genomic scaffold, scaffold_98
Master_Control_39145	Coffea canephora DH200=94 genomic scaffold, scaffold_98
Master_Control_10148	NA
Master_Control_10220	NA
Master_Control_1024	NA
Master_Control_10294	NA
Master_Control_10309	NA
Master_Control_10319	NA
Master_Control_10417	NA
Master_Control_10436	NA
Master_Control_10578	NA
Master_Control_105955	NA
Master_Control_10645	NA
Master_Control_1067	NA
Master_Control_10707	NA
Master_Control_1072	NA
Master_Control_10755	NA
Master_Control_10815	NA
Master_Control_10834	NA
Master_Control_10859	NA
Master_Control_10920	NA
Master_Control_11021	NA
Master_Control_110658	NA
Master_Control_11099	NA
Master_Control_1119	NA
Master_Control_11268	NA
Master_Control_113683	NA
Master_Control_11452	NA
Master_Control_11456	NA
Master_Control_115667	NA
Master_Control_11589	NA
Master_Control_116010	NA
Master_Control_11608	NA
Master_Control_11665	NA
Master_Control_1169	NA
Master_Control_11700	NA
Master_Control_11702	NA
Master_Control_117317	NA
Master_Control_11762	NA
Master_Control_11816	NA
Master_Control_11892	NA
Master_Control_11895	NA
Master_Control_119436	NA
Master_Control_11950	NA
Master_Control_11959	NA
Master_Control_120304	NA
Master_Control_120507	NA
Master_Control_12052	NA

Master_Control_121131	NA
Master_Control_121422	NA
Master_Control_12144	NA
Master_Control_12145	NA
Master_Control_12168	NA
Master_Control_121944	NA
Master_Control_12291	NA
Master_Control_123299	NA
Master_Control_12338	NA
Master_Control_1234	NA
Master_Control_12385	NA
Master_Control_12558	NA
Master_Control_125949	NA
Master_Control_125956	NA
Master_Control_12599	NA
Master_Control_126881	NA
Master_Control_12698	NA
Master_Control_12702	NA
Master_Control_12819	NA
Master_Control_12842	NA
Master_Control_12874	NA
Master_Control_12898	NA
Master_Control_12910	NA
Master_Control_13023	NA
Master_Control_13101	NA
Master_Control_13175	NA
Master_Control_13213	NA
Master_Control_13283	NA
Master_Control_133034	NA
Master_Control_13406	NA
Master_Control_13435	NA
Master_Control_13459	NA
Master_Control_13472	NA
Master_Control_13548	NA
Master_Control_136595	NA
Master_Control_13667	NA
Master_Control_13748	NA
Master_Control_13770	NA
Master_Control_13800	NA
Master_Control_138815	NA
Master_Control_139391	NA
Master_Control_13990	NA
Master_Control_14047	NA
Master_Control_14067	NA
Master_Control_141545	NA
Master_Control_14159	NA
Master_Control_141673	NA
Master_Control_14178	NA
Master_Control_142907	NA
Master_Control_14295	NA
Master_Control_14328	NA
Master_Control_14329	NA

Master_Control_143727	NA
Master_Control_14388	NA
Master_Control_145	NA
Master_Control_146273	NA
Master_Control_14656	NA
Master_Control_1475	NA
Master_Control_14771	NA
Master_Control_147990	NA
Master_Control_1488	NA
Master_Control_148879	NA
Master_Control_149011	NA
Master_Control_149629	NA
Master_Control_14989	NA
Master_Control_150415	NA
Master_Control_15086	NA
Master_Control_150877	NA
Master_Control_15103	NA
Master_Control_15149	NA
Master_Control_15232	NA
Master_Control_15270	NA
Master_Control_153121	NA
Master_Control_153240	NA
Master_Control_15335	NA
Master_Control_153368	NA
Master_Control_15360	NA
Master_Control_15424	NA
Master_Control_154925	NA
Master_Control_15587	NA
Master_Control_15652	NA
Master_Control_156667	NA
Master_Control_15697	NA
Master_Control_15803	NA
Master_Control_15835	NA
Master_Control_15847	NA
Master_Control_159543	NA
Master_Control_159795	NA
Master_Control_16022	NA
Master_Control_16037	NA
Master_Control_161635	NA
Master_Control_161866	NA
Master_Control_16210	NA
Master_Control_16214	NA
Master_Control_162524	NA
Master_Control_162554	NA
Master_Control_162557	NA
Master_Control_162575	NA
Master_Control_16260	NA
Master_Control_162656	NA
Master_Control_16284	NA
Master_Control_1629	NA
Master_Control_162998	NA
Master_Control_16363	NA

Master_Control_1638	NA
Master_Control_163908	NA
Master_Control_164002	NA
Master_Control_16406	NA
Master_Control_164334	NA
Master_Control_164384	NA
Master_Control_164429	NA
Master_Control_16453	NA
Master_Control_165180	NA
Master_Control_16556	NA
Master_Control_16561	NA
Master_Control_166255	NA
Master_Control_16673	NA
Master_Control_166895	NA
Master_Control_16741	NA
Master_Control_16748	NA
Master_Control_167726	NA
Master_Control_167859	NA
Master_Control_167862	NA
Master_Control_168037	NA
Master_Control_168358	NA
Master_Control_1684	NA
Master_Control_16841	NA
Master_Control_168678	NA
Master_Control_16945	NA
Master_Control_169843	NA
Master_Control_170545	NA
Master_Control_17090	NA
Master_Control_17096	NA
Master_Control_171239	NA
Master_Control_171695	NA
Master_Control_1720	NA
Master_Control_17223	NA
Master_Control_1726	NA
Master_Control_17261	NA
Master_Control_17306	NA
Master_Control_17324	NA
Master_Control_17334	NA
Master_Control_17507	NA
Master_Control_17521	NA
Master_Control_17547	NA
Master_Control_17569	NA
Master_Control_17601	NA
Master_Control_176153	NA
Master_Control_177430	NA
Master_Control_17751	NA
Master_Control_17822	NA
Master_Control_179432	NA
Master_Control_17950	NA
Master_Control_18005	NA
Master_Control_18083	NA
Master_Control_182302	NA

Master_Control_18277	NA
Master_Control_1830	NA
Master_Control_18362	NA
Master_Control_18397	NA
Master_Control_18432	NA
Master_Control_184498	NA
Master_Control_184501	NA
Master_Control_185280	NA
Master_Control_18590	NA
Master_Control_18725	NA
Master_Control_187959	NA
Master_Control_188004	NA
Master_Control_18808	NA
Master_Control_18866	NA
Master_Control_18881	NA
Master_Control_18907	NA
Master_Control_18974	NA
Master_Control_190374	NA
Master_Control_19169	NA
Master_Control_1917	NA
Master_Control_192158	NA
Master_Control_192528	NA
Master_Control_19290	NA
Master_Control_1930	NA
Master_Control_19353	NA
Master_Control_19382	NA
Master_Control_193993	NA
Master_Control_194157	NA
Master_Control_19426	NA
Master_Control_19540	NA
Master_Control_19593	NA
Master_Control_19614	NA
Master_Control_19685	NA
Master_Control_1969	NA
Master_Control_19760	NA
Master_Control_198180	NA
Master_Control_19919	NA
Master_Control_199437	NA
Master_Control_20002	NA
Master_Control_20016	NA
Master_Control_20021	NA
Master_Control_200288	NA
Master_Control_20052	NA
Master_Control_20082	NA
Master_Control_20092	NA
Master_Control_201019	NA
Master_Control_20119	NA
Master_Control_201930	NA
Master_Control_202073	NA
Master_Control_202114	NA
Master_Control_20228	NA
Master_Control_202318	NA

Master_Control_20296	NA
Master_Control_2030	NA
Master_Control_203001	NA
Master_Control_203596	NA
Master_Control_203749	NA
Master_Control_204896	NA
Master_Control_20567	NA
Master_Control_20616	NA
Master_Control_20635	NA
Master_Control_20655	NA
Master_Control_20657	NA
Master_Control_206986	NA
Master_Control_20726	NA
Master_Control_20734	NA
Master_Control_208569	NA
Master_Control_20933	NA
Master_Control_20961	NA
Master_Control_21039	NA
Master_Control_21172	NA
Master_Control_21185	NA
Master_Control_21201	NA
Master_Control_21301	NA
Master_Control_21435	NA
Master_Control_21440	NA
Master_Control_21468	NA
Master_Control_21471	NA
Master_Control_2149	NA
Master_Control_2150	NA
Master_Control_21634	NA
Master_Control_21835	NA
Master_Control_21904	NA
Master_Control_21909	NA
Master_Control_22004	NA
Master_Control_22112	NA
Master_Control_22166	NA
Master_Control_22207	NA
Master_Control_22266	NA
Master_Control_22329	NA
Master_Control_22361	NA
Master_Control_22368	NA
Master_Control_22392	NA
Master_Control_22433	NA
Master_Control_22510	NA
Master_Control_22562	NA
Master_Control_22619	NA
Master_Control_22678	NA
Master_Control_22701	NA
Master_Control_22724	NA
Master_Control_22725	NA
Master_Control_22746	NA
Master_Control_22823	NA
Master_Control_22829	NA

Master_Control_22918	NA
Master_Control_22938	NA
Master_Control_22964	NA
Master_Control_22965	NA
Master_Control_22972	NA
Master_Control_23195	NA
Master_Control_2323	NA
Master_Control_23323	NA
Master_Control_2349	NA
Master_Control_2351	NA
Master_Control_23547	NA
Master_Control_2359	NA
Master_Control_23622	NA
Master_Control_23709	NA
Master_Control_23713	NA
Master_Control_23735	NA
Master_Control_23820	NA
Master_Control_23853	NA
Master_Control_23897	NA
Master_Control_24249	NA
Master_Control_24253	NA
Master_Control_24305	NA
Master_Control_24420	NA
Master_Control_24582	NA
Master_Control_24583	NA
Master_Control_24642	NA
Master_Control_24711	NA
Master_Control_24713	NA
Master_Control_24747	NA
Master_Control_24921	NA
Master_Control_24928	NA
Master_Control_24964	NA
Master_Control_25014	NA
Master_Control_25068	NA
Master_Control_25147	NA
Master_Control_25261	NA
Master_Control_25297	NA
Master_Control_25404	NA
Master_Control_25556	NA
Master_Control_25579	NA
Master_Control_25588	NA
Master_Control_25590	NA
Master_Control_25683	NA
Master_Control_25753	NA
Master_Control_25837	NA
Master_Control_25857	NA
Master_Control_25930	NA
Master_Control_2601	NA
Master_Control_26054	NA
Master_Control_26122	NA
Master_Control_26124	NA
Master_Control_26140	NA

Master_Control_2623	NA
Master_Control_26456	NA
Master_Control_26618	NA
Master_Control_26648	NA
Master_Control_26708	NA
Master_Control_26789	NA
Master_Control_268	NA
Master_Control_26803	NA
Master_Control_26935	NA
Master_Control_27078	NA
Master_Control_2710	NA
Master_Control_27130	NA
Master_Control_2725	NA
Master_Control_27300	NA
Master_Control_27366	NA
Master_Control_27372	NA
Master_Control_27414	NA
Master_Control_27437	NA
Master_Control_27462	NA
Master_Control_27465	NA
Master_Control_27534	NA
Master_Control_27565	NA
Master_Control_27576	NA
Master_Control_27589	NA
Master_Control_27704	NA
Master_Control_27785	NA
Master_Control_2782	NA
Master_Control_27996	NA
Master_Control_28084	NA
Master_Control_28096	NA
Master_Control_28117	NA
Master_Control_28178	NA
Master_Control_2822	NA
Master_Control_28276	NA
Master_Control_28282	NA
Master_Control_28533	NA
Master_Control_28632	NA
Master_Control_2868	NA
Master_Control_28697	NA
Master_Control_28728	NA
Master_Control_28869	NA
Master_Control_28877	NA
Master_Control_28921	NA
Master_Control_28952	NA
Master_Control_28982	NA
Master_Control_29036	NA
Master_Control_29131	NA
Master_Control_29177	NA
Master_Control_29211	NA
Master_Control_29311	NA
Master_Control_29322	NA
Master_Control_29331	NA

Master_Control_29358	NA
Master_Control_29444	NA
Master_Control_29449	NA
Master_Control_295	NA
Master_Control_29645	NA
Master_Control_2967	NA
Master_Control_29699	NA
Master_Control_29739	NA
Master_Control_29829	NA
Master_Control_29899	NA
Master_Control_29946	NA
Master_Control_29962	NA
Master_Control_29981	NA
Master_Control_29990	NA
Master_Control_30157	NA
Master_Control_30179	NA
Master_Control_30184	NA
Master_Control_30197	NA
Master_Control_30291	NA
Master_Control_30556	NA
Master_Control_30601	NA
Master_Control_30632	NA
Master_Control_30763	NA
Master_Control_30823	NA
Master_Control_30831	NA
Master_Control_30868	NA
Master_Control_30937	NA
Master_Control_3094	NA
Master_Control_30946	NA
Master_Control_31106	NA
Master_Control_31169	NA
Master_Control_31223	NA
Master_Control_31243	NA
Master_Control_31245	NA
Master_Control_3125	NA
Master_Control_31257	NA
Master_Control_31308	NA
Master_Control_31319	NA
Master_Control_31369	NA
Master_Control_31665	NA
Master_Control_31671	NA
Master_Control_31788	NA
Master_Control_31807	NA
Master_Control_31826	NA
Master_Control_31889	NA
Master_Control_3191	NA
Master_Control_32200	NA
Master_Control_32206	NA
Master_Control_32250	NA
Master_Control_32286	NA
Master_Control_32291	NA
Master_Control_32299	NA

Master_Control_32327	NA
Master_Control_32336	NA
Master_Control_32372	NA
Master_Control_32446	NA
Master_Control_32452	NA
Master_Control_33461	NA
Master_Control_3351	NA
Master_Control_3356	NA
Master_Control_3453	NA
Master_Control_34656	NA
Master_Control_34841	NA
Master_Control_3522	NA
Master_Control_36095	NA
Master_Control_37533	NA
Master_Control_37785	NA
Master_Control_37902	NA
Master_Control_38112	NA
Master_Control_38294	NA
Master_Control_402	NA
Master_Control_4092	NA
Master_Control_4093	NA
Master_Control_41180	NA
Master_Control_4152	NA
Master_Control_4187	NA
Master_Control_42067	NA
Master_Control_4253	NA
Master_Control_42961	NA
Master_Control_42992	NA
Master_Control_44116	NA
Master_Control_4433	NA
Master_Control_4455	NA
Master_Control_4486	NA
Master_Control_4523	NA
Master_Control_4578	NA
Master_Control_45827	NA
Master_Control_4605	NA
Master_Control_4619	NA
Master_Control_462	NA
Master_Control_4625	NA
Master_Control_470	NA
Master_Control_4789	NA
Master_Control_4862	NA
Master_Control_4864	NA
Master_Control_5043	NA
Master_Control_5102	NA
Master_Control_5159	NA
Master_Control_52959	NA
Master_Control_5433	NA
Master_Control_5478	NA
Master_Control_5608	NA
Master_Control_5683	NA
Master_Control_5782	NA

Master_Control_5880	NA
Master_Control_5889	NA
Master_Control_5908	NA
Master_Control_59756	NA
Master_Control_601	NA
Master_Control_6068	NA
Master_Control_6073	NA
Master_Control_6140	NA
Master_Control_6191	NA
Master_Control_62051	NA
Master_Control_6258	NA
Master_Control_637	NA
Master_Control_64074	NA
Master_Control_6439	NA
Master_Control_6470	NA
Master_Control_65259	NA
Master_Control_6638	NA
Master_Control_66683	NA
Master_Control_67650	NA
Master_Control_6785	NA
Master_Control_6806	NA
Master_Control_6848	NA
Master_Control_6850	NA
Master_Control_68783	NA
Master_Control_69123	NA
Master_Control_69142	NA
Master_Control_6916	NA
Master_Control_6931	NA
Master_Control_6991	NA
Master_Control_7022	NA
Master_Control_7147	NA
Master_Control_71493	NA
Master_Control_717	NA
Master_Control_7170	NA
Master_Control_71705	NA
Master_Control_72011	NA
Master_Control_7262	NA
Master_Control_7297	NA
Master_Control_7325	NA
Master_Control_7388	NA
Master_Control_7415	NA
Master_Control_75156	NA
Master_Control_75272	NA
Master_Control_7568	NA
Master_Control_7574	NA
Master_Control_7606	NA
Master_Control_7638	NA
Master_Control_767	NA
Master_Control_7864	NA
Master_Control_79107	NA
Master_Control_79108	NA
Master_Control_8083	NA

Master_Control_8113	NA
Master_Control_8129	NA
Master_Control_8292	NA
Master_Control_8321	NA
Master_Control_8342	NA
Master_Control_836	NA
Master_Control_8364	NA
Master_Control_8371	NA
Master_Control_8530	NA
Master_Control_85407	NA
Master_Control_8556	NA
Master_Control_86030	NA
Master_Control_8607	NA
Master_Control_8641	NA
Master_Control_86905	NA
Master_Control_8796	NA
Master_Control_8820	NA
Master_Control_8824	NA
Master_Control_8892	NA
Master_Control_8902	NA
Master_Control_8909	NA
Master_Control_8990	NA
Master_Control_9008	NA
Master_Control_9039	NA
Master_Control_9047	NA
Master_Control_9052	NA
Master_Control_9080	NA
Master_Control_9093	NA
Master_Control_9123	NA
Master_Control_9224	NA
Master_Control_9325	NA
Master_Control_933	NA
Master_Control_9422	NA
Master_Control_9431	NA
Master_Control_9463	NA
Master_Control_9476	NA
Master_Control_9496	NA
Master_Control_95609	NA
Master_Control_9561	NA
Master_Control_9566	NA
Master_Control_9618	NA
Master_Control_9648	NA
Master_Control_9670	NA
Master_Control_9816	NA
Master_Control_9873	NA
Master_Control_9892	NA
Master_Control_9960	NA
Master_Control_9991	NA
Master_Control_10914	Putative uncharacterized protein
Master_Control_13009	Putative uncharacterized protein
Master_Control_13282	Putative uncharacterized protein
Master_Control_13339	Putative uncharacterized protein

Master_Control_13426	Putative uncharacterized protein
Master_Control_13660	Putative uncharacterized protein
Master_Control_139306	Putative uncharacterized protein
Master_Control_140875	Putative uncharacterized protein
Master_Control_15583	Putative uncharacterized protein
Master_Control_15887	Putative uncharacterized protein
Master_Control_16367	Putative uncharacterized protein
Master_Control_16566	Putative uncharacterized protein
Master_Control_166097	Putative uncharacterized protein
Master_Control_16970	Putative uncharacterized protein
Master_Control_173634	Putative uncharacterized protein
Master_Control_17434	Putative uncharacterized protein
Master_Control_17465	Putative uncharacterized protein
Master_Control_175241	Putative uncharacterized protein
Master_Control_177747	Putative uncharacterized protein
Master_Control_17923	Putative uncharacterized protein
Master_Control_179640	Putative uncharacterized protein
Master_Control_18002	Putative uncharacterized protein
Master_Control_18042	Putative uncharacterized protein
Master_Control_18056	Putative uncharacterized protein
Master_Control_18424	Putative uncharacterized protein
Master_Control_18664	Putative uncharacterized protein
Master_Control_18732	Putative uncharacterized protein
Master_Control_18776	Putative uncharacterized protein
Master_Control_19055	Putative uncharacterized protein
Master_Control_19139	Putative uncharacterized protein
Master_Control_19537	Putative uncharacterized protein
Master_Control_19818	Putative uncharacterized protein
Master_Control_19877	Putative uncharacterized protein
Master_Control_19880	Putative uncharacterized protein
Master_Control_20490	Putative uncharacterized protein
Master_Control_20962	Putative uncharacterized protein
Master_Control_20963	Putative uncharacterized protein
Master_Control_210	Putative uncharacterized protein
Master_Control_21624	Putative uncharacterized protein
Master_Control_21839	Putative uncharacterized protein
Master_Control_22084	Putative uncharacterized protein
Master_Control_22160	Putative uncharacterized protein
Master_Control_22371	Putative uncharacterized protein
Master_Control_22713	Putative uncharacterized protein
Master_Control_23146	Putative uncharacterized protein
Master_Control_23624	Putative uncharacterized protein
Master_Control_24037	Putative uncharacterized protein
Master_Control_24165	Putative uncharacterized protein
Master_Control_25656	Putative uncharacterized protein
Master_Control_25762	Putative uncharacterized protein
Master_Control_26290	Putative uncharacterized protein
Master_Control_26610	Putative uncharacterized protein
Master_Control_2841	Putative uncharacterized protein
Master_Control_28772	Putative uncharacterized protein
Master_Control_28774	Putative uncharacterized protein
Master_Control_29130	Putative uncharacterized protein

Master_Control_29313	Putative uncharacterized protein
Master_Control_29408	Putative uncharacterized protein
Master_Control_29677	Putative uncharacterized protein
Master_Control_29766	Putative uncharacterized protein
Master_Control_29987	Putative uncharacterized protein
Master_Control_30177	Putative uncharacterized protein
Master_Control_30658	Putative uncharacterized protein
Master_Control_30871	Putative uncharacterized protein
Master_Control_31081	Putative uncharacterized protein
Master_Control_31284	Putative uncharacterized protein
Master_Control_31914	Putative uncharacterized protein
Master_Control_32227	Putative uncharacterized protein
Master_Control_3347	Putative uncharacterized protein
Master_Control_3591	Putative uncharacterized protein
Master_Control_4282	Putative uncharacterized protein
Master_Control_4718	Putative uncharacterized protein
Master_Control_50303	Putative uncharacterized protein
Master_Control_58525	Putative uncharacterized protein
Master_Control_5942	Putative uncharacterized protein
Master_Control_6127	Putative uncharacterized protein
Master_Control_6225	Putative uncharacterized protein
Master_Control_6565	Putative uncharacterized protein
Master_Control_6790	Putative uncharacterized protein
Master_Control_76929	Putative uncharacterized protein
Master_Control_7898	Putative uncharacterized protein
Master_Control_8314	Putative uncharacterized protein
Master_Control_8408	Putative uncharacterized protein
Master_Control_85973	Putative uncharacterized protein
Master_Control_8893	Putative uncharacterized protein
Master_Control_918	Putative uncharacterized protein
Master_Control_9337	Putative uncharacterized protein
Master_Control_9515	Putative uncharacterized protein
Master_Control_97729	Putative uncharacterized protein
Master_Control_9862	Putative uncharacterized protein
Master_Control_10262	Putative uncharacterized protein (Fragment)
Master_Control_193018	Putative uncharacterized protein At2g36410 (Uncharacterized protein)
Master_Control_22431	Putative uncharacterized protein Sb02g006460
Master_Control_100097	Uncharacterized protein
Master_Control_10107	Uncharacterized protein
Master_Control_10564	Uncharacterized protein
Master_Control_10748	Uncharacterized protein
Master_Control_1084	Uncharacterized protein
Master_Control_10886	Uncharacterized protein
Master_Control_10917	Uncharacterized protein
Master_Control_10948	Uncharacterized protein
Master_Control_109579	Uncharacterized protein
Master_Control_11110	Uncharacterized protein
Master_Control_11145	Uncharacterized protein
Master_Control_11326	Uncharacterized protein
Master_Control_11436	Uncharacterized protein
Master_Control_11454	Uncharacterized protein
Master_Control_11528	Uncharacterized protein

Master_Control_11758	Uncharacterized protein
Master_Control_1176	Uncharacterized protein
Master_Control_11770	Uncharacterized protein
Master_Control_118565	Uncharacterized protein
Master_Control_11946	Uncharacterized protein
Master_Control_12067	Uncharacterized protein
Master_Control_122516	Uncharacterized protein
Master_Control_12421	Uncharacterized protein
Master_Control_12456	Uncharacterized protein
Master_Control_12670	Uncharacterized protein
Master_Control_127364	Uncharacterized protein
Master_Control_127800	Uncharacterized protein
Master_Control_12953	Uncharacterized protein
Master_Control_13048	Uncharacterized protein
Master_Control_13172	Uncharacterized protein
Master_Control_13332	Uncharacterized protein
Master_Control_13354	Uncharacterized protein
Master_Control_13460	Uncharacterized protein
Master_Control_13468	Uncharacterized protein
Master_Control_13572	Uncharacterized protein
Master_Control_138870	Uncharacterized protein
Master_Control_13891	Uncharacterized protein
Master_Control_139104	Uncharacterized protein
Master_Control_140124	Uncharacterized protein
Master_Control_14031	Uncharacterized protein
Master_Control_14352	Uncharacterized protein
Master_Control_14704	Uncharacterized protein
Master_Control_14962	Uncharacterized protein
Master_Control_15289	Uncharacterized protein
Master_Control_1534	Uncharacterized protein
Master_Control_15502	Uncharacterized protein
Master_Control_15540	Uncharacterized protein
Master_Control_15623	Uncharacterized protein
Master_Control_16118	Uncharacterized protein
Master_Control_16137	Uncharacterized protein
Master_Control_1628	Uncharacterized protein
Master_Control_162992	Uncharacterized protein
Master_Control_16583	Uncharacterized protein
Master_Control_166463	Uncharacterized protein
Master_Control_16647	Uncharacterized protein
Master_Control_16707	Uncharacterized protein
Master_Control_16730	Uncharacterized protein
Master_Control_167546	Uncharacterized protein
Master_Control_16872	Uncharacterized protein
Master_Control_16896	Uncharacterized protein
Master_Control_17012	Uncharacterized protein
Master_Control_17019	Uncharacterized protein
Master_Control_1719	Uncharacterized protein
Master_Control_17233	Uncharacterized protein
Master_Control_17250	Uncharacterized protein
Master_Control_17309	Uncharacterized protein
Master_Control_17328	Uncharacterized protein

Master_Control_17340	Uncharacterized protein
Master_Control_174376	Uncharacterized protein
Master_Control_17464	Uncharacterized protein
Master_Control_17512	Uncharacterized protein
Master_Control_17517	Uncharacterized protein
Master_Control_17534	Uncharacterized protein
Master_Control_176106	Uncharacterized protein
Master_Control_1763	Uncharacterized protein
Master_Control_17678	Uncharacterized protein
Master_Control_177154	Uncharacterized protein
Master_Control_177211	Uncharacterized protein
Master_Control_177486	Uncharacterized protein
Master_Control_17847	Uncharacterized protein
Master_Control_17880	Uncharacterized protein
Master_Control_17943	Uncharacterized protein
Master_Control_179790	Uncharacterized protein
Master_Control_185587	Uncharacterized protein
Master_Control_185882	Uncharacterized protein
Master_Control_186113	Uncharacterized protein
Master_Control_18642	Uncharacterized protein
Master_Control_18656	Uncharacterized protein
Master_Control_18692	Uncharacterized protein
Master_Control_18704	Uncharacterized protein
Master_Control_18748	Uncharacterized protein
Master_Control_188311	Uncharacterized protein
Master_Control_18921	Uncharacterized protein
Master_Control_189242	Uncharacterized protein
Master_Control_189960	Uncharacterized protein
Master_Control_19141	Uncharacterized protein
Master_Control_191570	Uncharacterized protein
Master_Control_191786	Uncharacterized protein
Master_Control_19253	Uncharacterized protein
Master_Control_1948	Uncharacterized protein
Master_Control_19520	Uncharacterized protein
Master_Control_19636	Uncharacterized protein
Master_Control_19981	Uncharacterized protein
Master_Control_20206	Uncharacterized protein
Master_Control_20207	Uncharacterized protein
Master_Control_20315	Uncharacterized protein
Master_Control_20318	Uncharacterized protein
Master_Control_20527	Uncharacterized protein
Master_Control_20709	Uncharacterized protein
Master_Control_20856	Uncharacterized protein
Master_Control_20939	Uncharacterized protein
Master_Control_21064	Uncharacterized protein
Master_Control_21162	Uncharacterized protein
Master_Control_21264	Uncharacterized protein
Master_Control_21520	Uncharacterized protein
Master_Control_21604	Uncharacterized protein
Master_Control_21861	Uncharacterized protein
Master_Control_22293	Uncharacterized protein
Master_Control_22525	Uncharacterized protein

Master_Control_23002	Uncharacterized protein
Master_Control_23015	Uncharacterized protein
Master_Control_23111	Uncharacterized protein
Master_Control_23136	Uncharacterized protein
Master_Control_23174	Uncharacterized protein
Master_Control_23184	Uncharacterized protein
Master_Control_23325	Uncharacterized protein
Master_Control_2338	Uncharacterized protein
Master_Control_23454	Uncharacterized protein
Master_Control_23731	Uncharacterized protein
Master_Control_23826	Uncharacterized protein
Master_Control_24050	Uncharacterized protein
Master_Control_24151	Uncharacterized protein
Master_Control_24245	Uncharacterized protein
Master_Control_2436	Uncharacterized protein
Master_Control_24863	Uncharacterized protein
Master_Control_24870	Uncharacterized protein
Master_Control_24924	Uncharacterized protein
Master_Control_24960	Uncharacterized protein
Master_Control_25119	Uncharacterized protein
Master_Control_2531	Uncharacterized protein
Master_Control_25721	Uncharacterized protein
Master_Control_25769	Uncharacterized protein
Master_Control_25866	Uncharacterized protein
Master_Control_2602	Uncharacterized protein
Master_Control_2627	Uncharacterized protein
Master_Control_26316	Uncharacterized protein
Master_Control_26365	Uncharacterized protein
Master_Control_26474	Uncharacterized protein
Master_Control_26720	Uncharacterized protein
Master_Control_26790	Uncharacterized protein
Master_Control_26809	Uncharacterized protein
Master_Control_27050	Uncharacterized protein
Master_Control_27051	Uncharacterized protein
Master_Control_2720	Uncharacterized protein
Master_Control_2764	Uncharacterized protein
Master_Control_28234	Uncharacterized protein
Master_Control_28248	Uncharacterized protein
Master_Control_28333	Uncharacterized protein
Master_Control_28530	Uncharacterized protein
Master_Control_28554	Uncharacterized protein
Master_Control_28556	Uncharacterized protein
Master_Control_28610	Uncharacterized protein
Master_Control_2875	Uncharacterized protein
Master_Control_28863	Uncharacterized protein
Master_Control_29028	Uncharacterized protein
Master_Control_29053	Uncharacterized protein
Master_Control_29066	Uncharacterized protein
Master_Control_29099	Uncharacterized protein
Master_Control_29138	Uncharacterized protein
Master_Control_29355	Uncharacterized protein
Master_Control_2968	Uncharacterized protein

Master_Control_29725	Uncharacterized protein
Master_Control_29940	Uncharacterized protein
Master_Control_2997	Uncharacterized protein
Master_Control_30264	Uncharacterized protein
Master_Control_30553	Uncharacterized protein
Master_Control_30600	Uncharacterized protein
Master_Control_30744	Uncharacterized protein
Master_Control_30862	Uncharacterized protein
Master_Control_30875	Uncharacterized protein
Master_Control_30922	Uncharacterized protein
Master_Control_30979	Uncharacterized protein
Master_Control_31155	Uncharacterized protein
Master_Control_31157	Uncharacterized protein
Master_Control_31268	Uncharacterized protein
Master_Control_31350	Uncharacterized protein
Master_Control_31360	Uncharacterized protein
Master_Control_3150	Uncharacterized protein
Master_Control_3196	Uncharacterized protein
Master_Control_32006	Uncharacterized protein
Master_Control_32100	Uncharacterized protein
Master_Control_32239	Uncharacterized protein
Master_Control_32249	Uncharacterized protein
Master_Control_32268	Uncharacterized protein
Master_Control_32415	Uncharacterized protein
Master_Control_3262	Uncharacterized protein
Master_Control_3386	Uncharacterized protein
Master_Control_34406	Uncharacterized protein
Master_Control_3447	Uncharacterized protein
Master_Control_3482	Uncharacterized protein
Master_Control_3486	Uncharacterized protein
Master_Control_3514	Uncharacterized protein
Master_Control_3542	Uncharacterized protein
Master_Control_3643	Uncharacterized protein
Master_Control_37305	Uncharacterized protein
Master_Control_37931	Uncharacterized protein
Master_Control_3849	Uncharacterized protein
Master_Control_3889	Uncharacterized protein
Master_Control_3893	Uncharacterized protein
Master_Control_39704	Uncharacterized protein
Master_Control_4038	Uncharacterized protein
Master_Control_4075	Uncharacterized protein
Master_Control_41046	Uncharacterized protein
Master_Control_42046	Uncharacterized protein
Master_Control_4384	Uncharacterized protein
Master_Control_4390	Uncharacterized protein
Master_Control_43920	Uncharacterized protein
Master_Control_43922	Uncharacterized protein
Master_Control_463	Uncharacterized protein
Master_Control_48759	Uncharacterized protein
Master_Control_4972	Uncharacterized protein
Master_Control_5012	Uncharacterized protein
Master_Control_5014	Uncharacterized protein

Master_Control_5138	Uncharacterized protein
Master_Control_5163	Uncharacterized protein
Master_Control_5286	Uncharacterized protein
Master_Control_52985	Uncharacterized protein
Master_Control_56130	Uncharacterized protein
Master_Control_5621	Uncharacterized protein
Master_Control_5625	Uncharacterized protein
Master_Control_5691	Uncharacterized protein
Master_Control_5703	Uncharacterized protein
Master_Control_5845	Uncharacterized protein
Master_Control_59357	Uncharacterized protein
Master_Control_6198	Uncharacterized protein
Master_Control_6314	Uncharacterized protein
Master_Control_6410	Uncharacterized protein
Master_Control_6478	Uncharacterized protein
Master_Control_6654	Uncharacterized protein
Master_Control_6679	Uncharacterized protein
Master_Control_6883	Uncharacterized protein
Master_Control_69987	Uncharacterized protein
Master_Control_7116	Uncharacterized protein
Master_Control_7152	Uncharacterized protein
Master_Control_7290	Uncharacterized protein
Master_Control_7395	Uncharacterized protein
Master_Control_7432	Uncharacterized protein
Master_Control_7534	Uncharacterized protein
Master_Control_7633	Uncharacterized protein
Master_Control_8252	Uncharacterized protein
Master_Control_8331	Uncharacterized protein
Master_Control_8374	Uncharacterized protein
Master_Control_8389	Uncharacterized protein
Master_Control_8675	Uncharacterized protein
Master_Control_8694	Uncharacterized protein
Master_Control_8923	Uncharacterized protein
Master_Control_9247	Uncharacterized protein
Master_Control_9398	Uncharacterized protein
Master_Control_9443	Uncharacterized protein
Master_Control_9525	Uncharacterized protein
Master_Control_9586	Uncharacterized protein
Master_Control_959	Uncharacterized protein
Master_Control_9974	Uncharacterized protein
Master_Control_12971	Uncharacterized protein (Fragment)
Master_Control_157	Uncharacterized protein (Fragment)
Master_Control_1573	Uncharacterized protein (Fragment)
Master_Control_17940	Uncharacterized protein (Fragment)
Master_Control_20293	Uncharacterized protein (Fragment)
Master_Control_22007	Uncharacterized protein (Fragment)
Master_Control_25920	Uncharacterized protein (Fragment)
Master_Control_26401	Uncharacterized protein (Fragment)
Master_Control_27888	Uncharacterized protein (Fragment)
Master_Control_29054	Uncharacterized protein (Fragment)
Master_Control_29542	Uncharacterized protein (Fragment)
Master_Control_3101	Uncharacterized protein (Fragment)

Master_Control_31902	Uncharacterized protein (Fragment)
Master_Control_3758	Uncharacterized protein (Fragment)
Master_Control_4090	Uncharacterized protein (Fragment)
Master_Control_4744	Uncharacterized protein (Fragment)
Master_Control_5037	Uncharacterized protein (Fragment)
Master_Control_5648	Uncharacterized protein (Fragment)
Master_Control_6842	Uncharacterized protein (Fragment)
Master_Control_7220	Uncharacterized protein (Fragment)
Master_Control_81565	Uncharacterized protein (Fragment)
Master_Control_9083	Uncharacterized protein (Fragment)

Table S10. List of primers

Primer	Sequence
**Real time primers**	**Primer sequence**
mc_15684_F	5’ TGACATAGGTTCATTGCGGAAA 3’
mc_15684_R	5’ ACAAGTGGCTAAGTGACATGTTTG 3’
mc_17923_F	5’ CAGTACCCGTAGAGCTTCACAATG 3’
mc_17923_R	5’ TTGATCGCAACAGCTTCTTGA 3’
mc_18042_F	5’ CAGACAAGGTAGCGATGGACTCT 3’
mc_18042_R	5’ TTGGTGCTTTCATTCACTCGAA 3’
mc_125770_F	5’ TTTGCCGTGGTTGAATCATG 3’
mc_125770_R	5’ TGATGGTAAATTAAACACCGAGCTAA 3’
mc_126262_F	5’ GCTCCAATAATTGCTGCCACTA 3’
mc_126262_R	5’ TGGGAATGATGGGCTCTCTT 3’
mc_105730_F	5’ GTTCTGGGCCGAAGTTTGAC 3’
mc_105730_R	5’ GCCACATTGGCTCTTATTGGA 3’
mc_113386_F	5’ CGAATGACAGATCGTTCAAAGC 3’
mc_113386_R	5’ CCCAGTTGATCCACCGTTCT 3’
ADS_F	5’ GAATGGTGACCGCTCTTCCTT 3’
ADS_R	5’ GATTGCTTGAACGCTCCATTTT 3’
DXS_F	5’ GTGGGACCGGGATGAATTTA 3’
DXS_R	5’ GTCCTTCGCACGCCAATC 3’
HMGR_F	5’ CCAAAAGCCTCAGACGCATT 3’
HMGR_R	5’ GATCTTGTCGCGCCATCTGT 3’
mc_76560_F	5’ TGATAAGTTTGCGCTTTGTTTAGC 3’
mc_76560_R	5’ AAGGCCCGTCGCCTAAAA 3’
mc_3514_F	5’ TGACCCAATAATCGAATCAAGACTT 3’
mc_3514_R	5’ TGCTTCAAGGCGATTCATCA 3’
mc_122443_F	5’ AGCAAGTTCAAAGGCTTTCAAAA 3’
mc_122443_R	5’ GATCTCTCGATTGGATGTGTATGC 3’
mc_11791_F	5’ TGCCGACGTTTGGGATGT 3’
mc_11791_R	5’ CCCGTAGGCGTGCCACTA 3’
FPPS_F	5’ CGATGATTCCCGTCAATGG 3’
FPPS_R	5’ CCCCGGTTCAGCTTTCCT 3’

DBR2_F	5’ AGCATCCCATCAAGTGTACCAA 3’
DBR2_R	5’ AATGGGCTTGCTTGTTGATGA 3’
ACTIN_F	5’ GAATGGTTAAGGCTGGATTTGCA 3’
ACTIN_R	5’ CGGTAATTTCCTTGCTCATCCTATC 3’
mc_43833_F	5’ GCCTATCTGGTGCCCATGAC 3’
mc_43833_R	5’ TGTTATCCCAAAAGCAAACGATT 3’
mc_96454_F	5’ CAGACCGTCTAACTGCTCATGAA 3’
mc_96454_R	5’ TCAGGAGCAACACCATTTTCG 3’
EGF-1alpha_F	5’ AGCCCAAGAGACCATCAGACA 3’
EGF-1alpha_R	5’TTCCAATACCACCAATCTTGTAAACA 3’
**Primers (for stress specific TFs amplification)**	**Primer sequence**
c22337_g1_i1_F	5’ CACACTTGCTTCCTCTACC 3’
c22337_g1_i1_R	5’ GTCACTAGACTGCGGGATAG 3’
c27716_g3_i4_F	5’ GTTTAAAGTACACACATAGTTAAGAAAC 3’
c27716_g3_i4_R	5’ GTTGACTCCTCCATCTTCTTA 3’
c20206_g1_i3_F	5’ TGTGAACCAAATTATATCCATCTA 3’
c20206_g1_i3_R	5’ ATCAAAACGCATGTCATTG 3’
c44388_g1_i1_F	TTGTTCATAGAAAGCGATTAA
c44388_g1_i1_R	TCGAAGCTAATATAAATGCACA
c42073_g1_i1_F	ATACTATCTACTGCTCCATCTCGA
c42073_g1_i1_R	TTTTGAAGCCTAGTGGGA
c21577_g1_i1_F	GTGAAACTACCAACAAATCTATCCATA
c21577_g1_i1_R	GCATTGAATCCTGACTTGTTT
c44152_g1_i1_F	TCTTCAAGATTTGCTAGGGTT
c44152_g1_i1_R	CTTAACACACATTTTTGCTTTC
c31649_g1_i3_F	CCCAACTGAAACAAGAAGAAA
c31649_g1_i3_R	GTTACCATGTGCACACCAAA
c31442_g4_i1_F	GAAAGCATAGTATTTATAGTACTGAAT
c31442_g4_i1_R	TATACGACTATACGGCTGATG
c16201_g1_i1_F	CAGCCTCAAAGAAAAAGAAAG
c16201_g1_i1_R	TCATACTTGGTGGAATATAAAATT

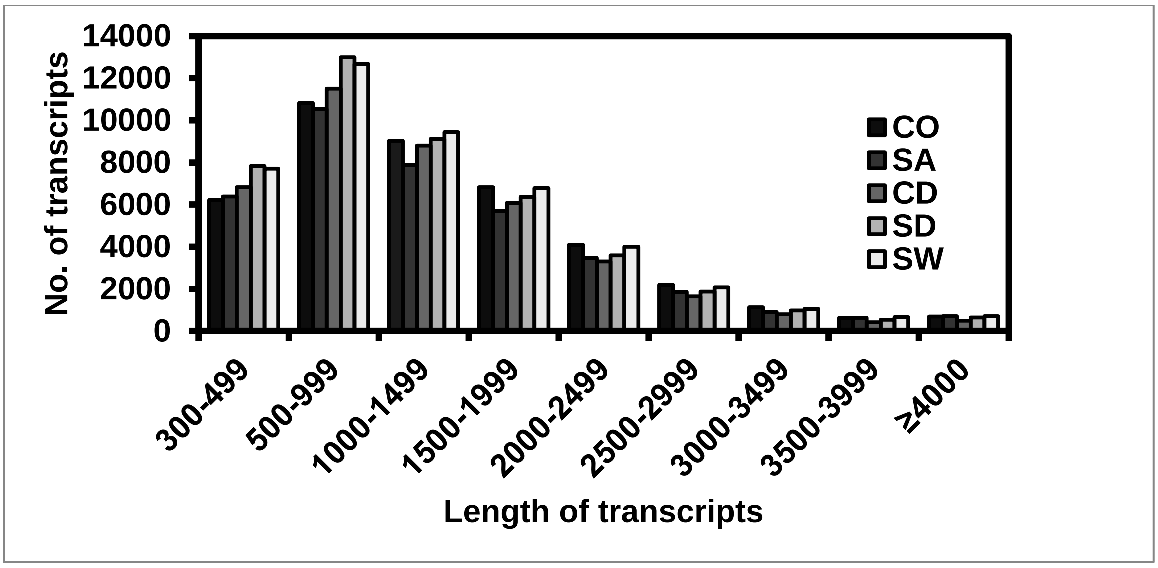

Figure S1. Size distribution of the assembled transcripts. Length of assembled transcripts *vs.* Number of transcripts plotted for *A. annua* control(CO), salinity(SA), cold(CD), severe drought(SD) and severe water-logging(SW) stress samples.

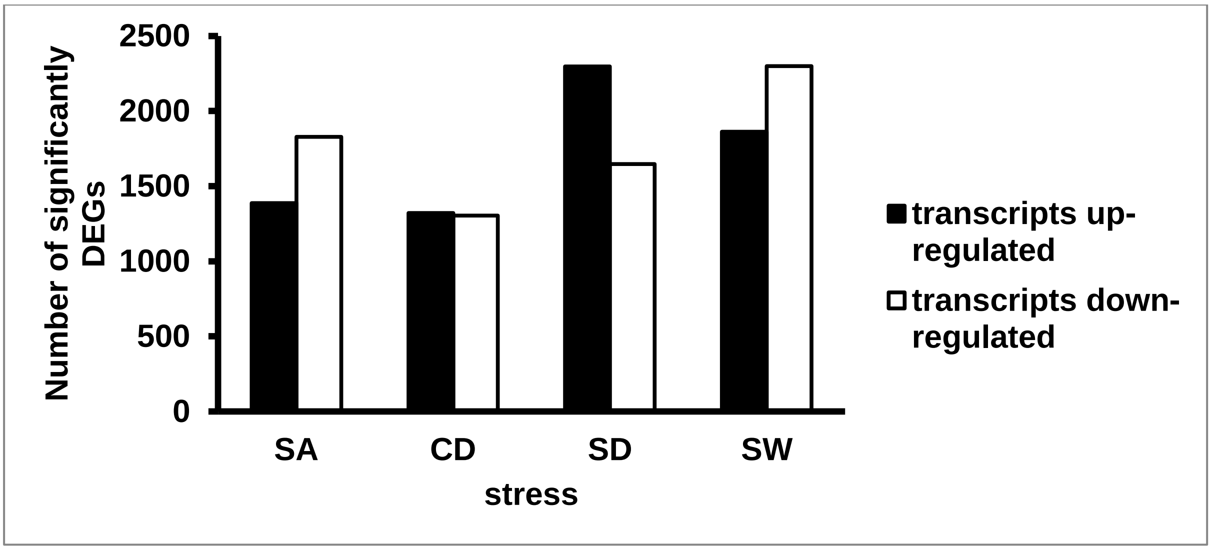

Figure S2. The number of DEGs up- and down- regulated in each library. The DEGs were identified using a threshold of p value≤0.05 and log2 fold change≥1

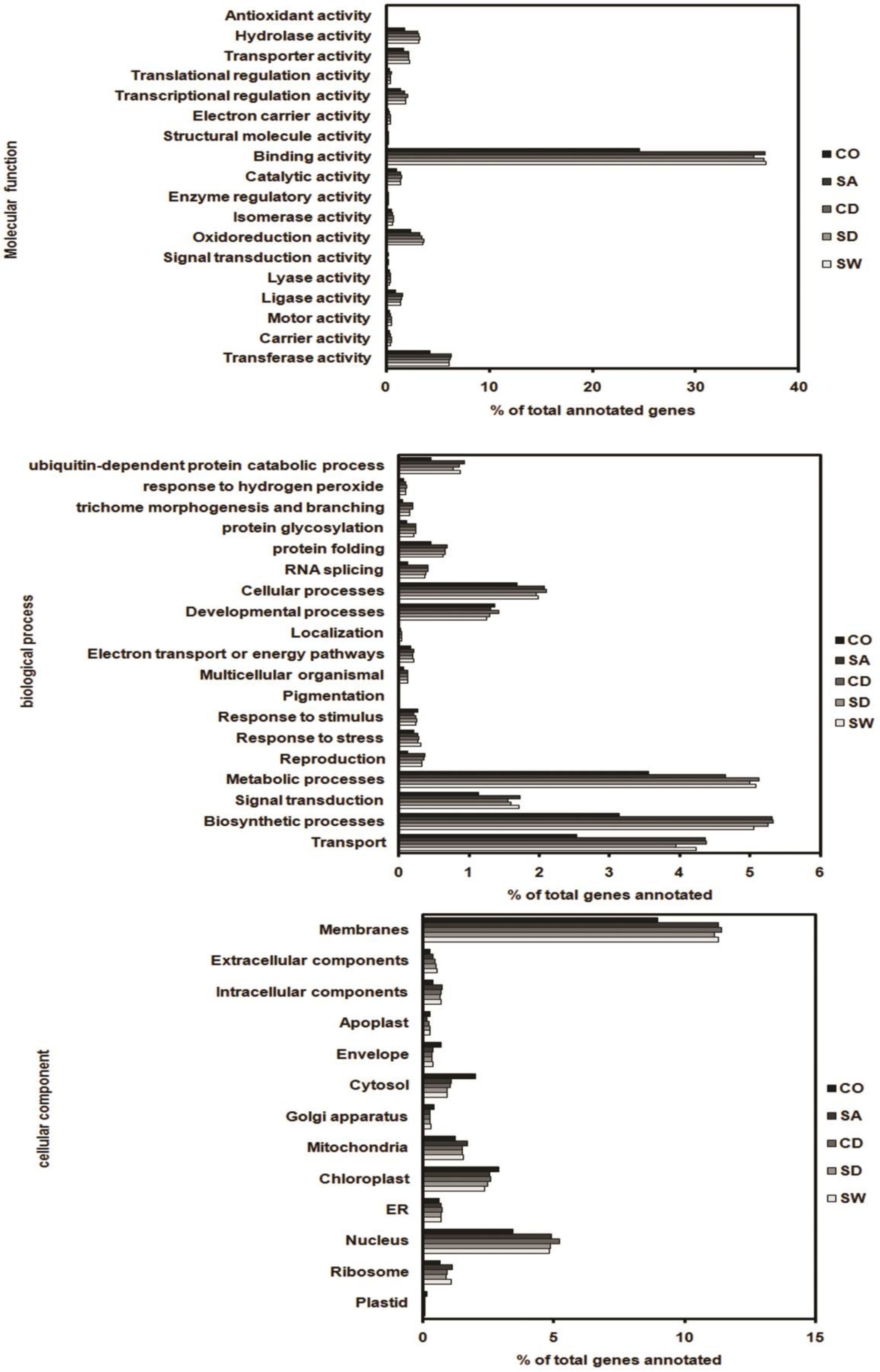

Figure S3. Distribution of *A. annua* CO, SA, CD, SD and SW transcripts across various GO terms.

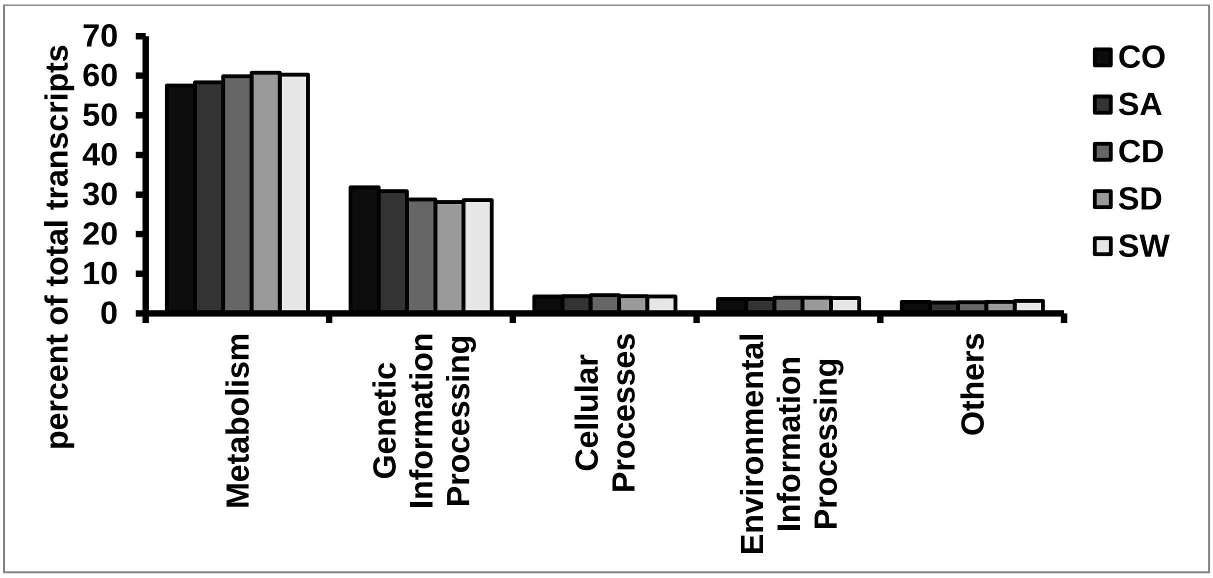

Figure S4. KEGG classification.

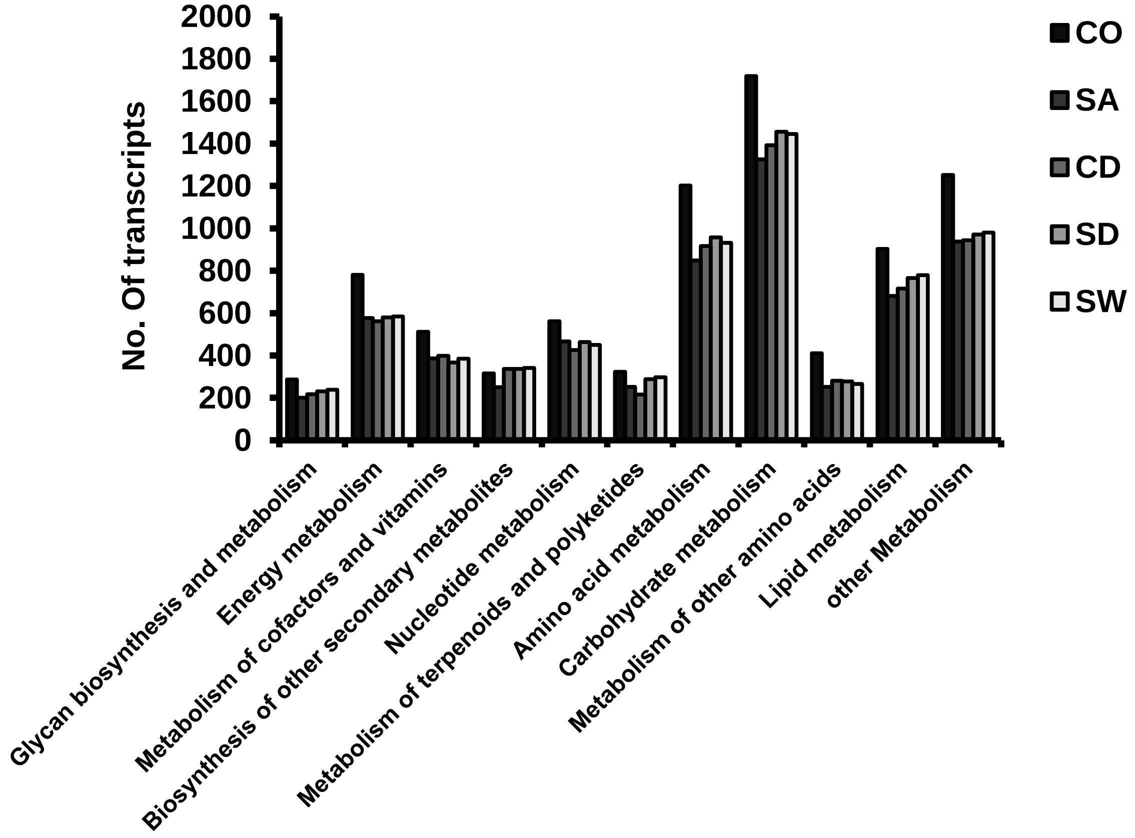

Figure S5. KEGG classification based on metabolism categories. Transcripts of *A. annua* control(CO), salinity(SA), cold(CD), drought(SD) and water-logging(SW) samples assigned to metabolism category were classified to 11 groups based on metabolism categories of KEGG.

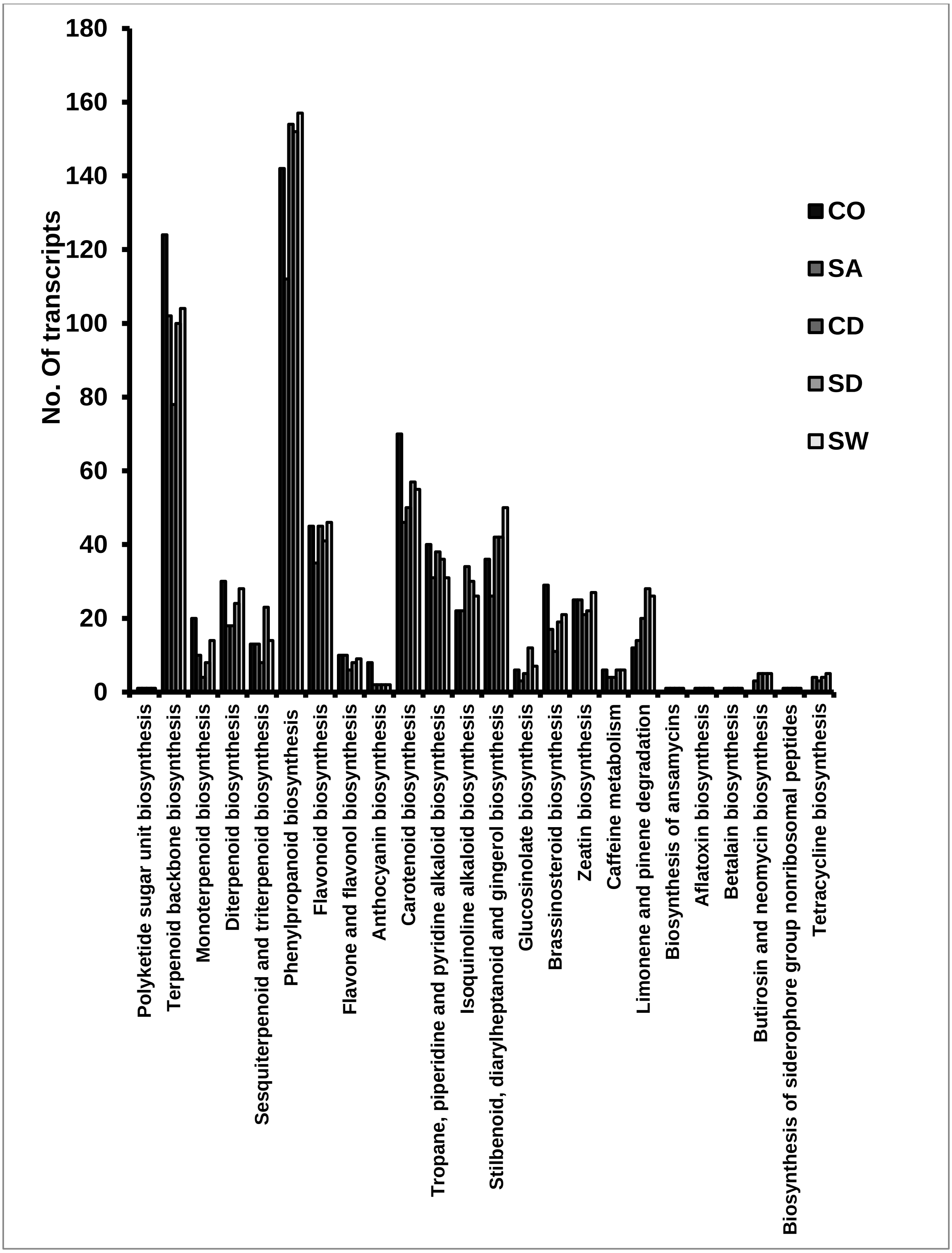

Figure S6. KEGG classification based on secondary metabolism categories. Transcripts of *A. annua* control(CO), salinity(SA), cold(CD), drought(SD) and water-logging(SW) samples assigned to secondary metabolism category were classified in 24 groups based on secondary metabolism categories of KEGG.

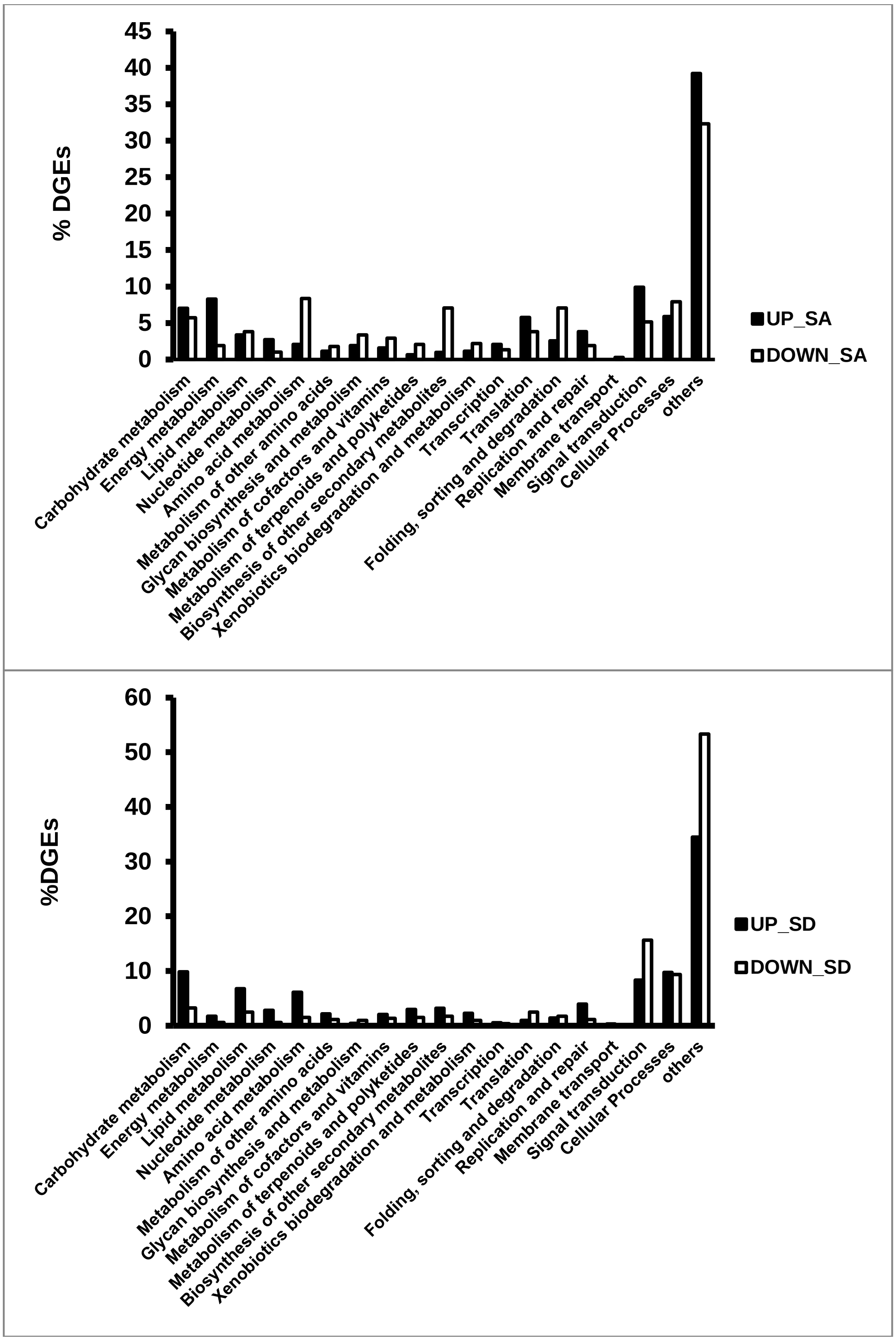

% DGEs
45
40
35
30
25
20
15
10
5
0
UP_SA
DOWN_SA
Carbohydrate metabolism
Energy metabolism
Lipid metabolism
Nucleotide metabolism
Amino acid metabolism
Metabolism of other amino acids
Glycan biosynthesis and metabolism
Metabolism of cofactors and vitamins
Metabolism of terpenoids and polyketides
Biosynthesis of other secondary metabolites
Xenobiotics biodegradation and metabolism
Transcription
Translation
Folding, sorting and degradation
Replication and repair
Membrane transport
Signal transduction
Cellular Processes
others
%DGEs
60
50
40
30
20
10
0
UP_SD
DOWN_SD
Carbohydrate metabolism
Energy metabolism
Lipid metabolism
Nucleotide metabolism
Amino acid metabolism
Metabolism of other amino acids
Glycan biosynthesis and metabolism
Metabolism of cofactors and vitamins
Metabolism of terpenoids and polyketides
Biosynthesis of other secondary metabolites
Xenobiotics biodegradation and metabolism
Transcription
Translation
Folding, sorting and degradation
Replication and repair
Membrane transport
Signal transduction
Cellular Processes
others

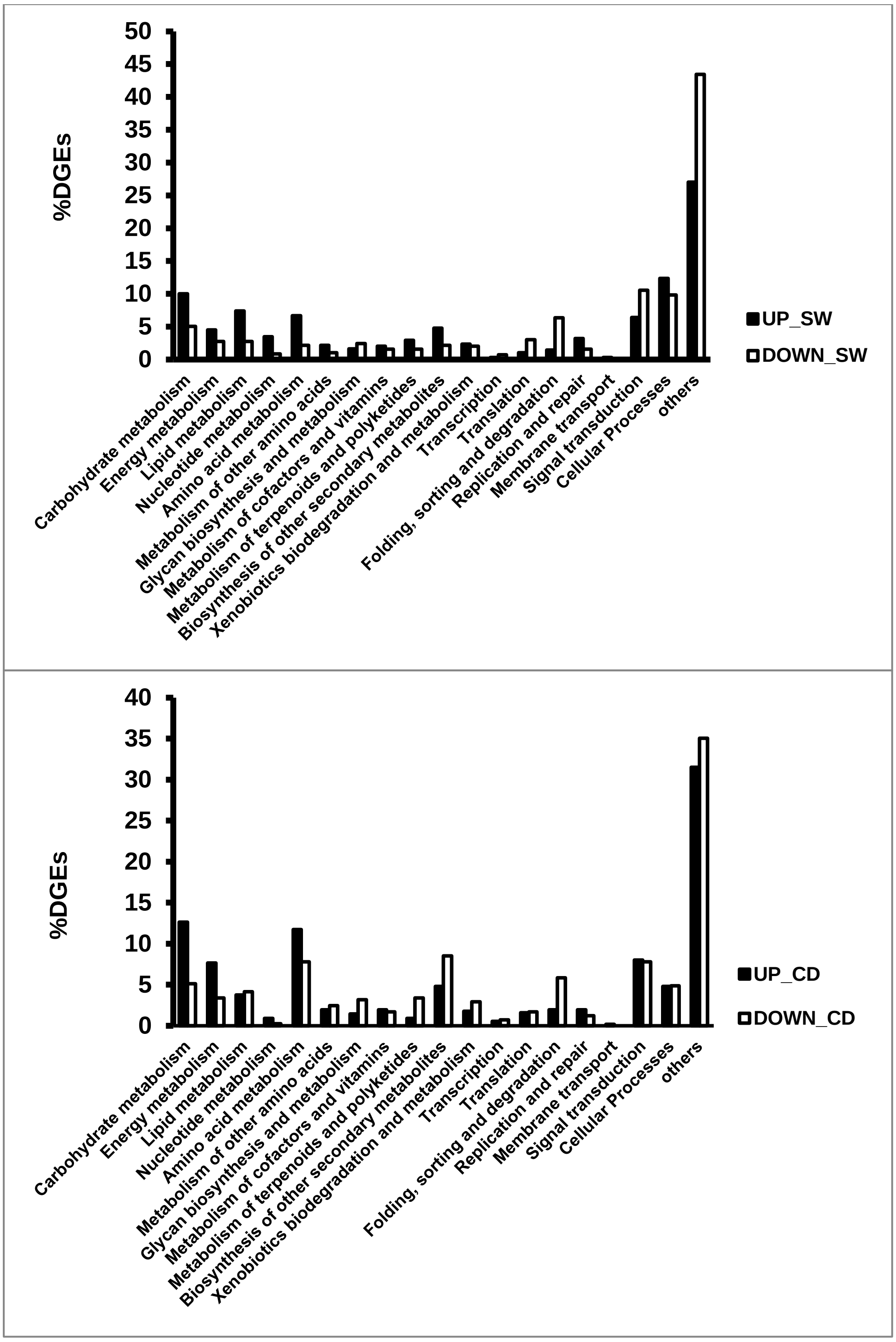

Figure S7. KEGG classification for significantly differentially expressed genes. Transcripts of *A. annua* showing significant differential expression (log2fold change≥1 and p value≤0.05) in different stresses, SA(salinity),CD(cold),SD(drought) and SW(water-logging), were assigned to KEGG categories.

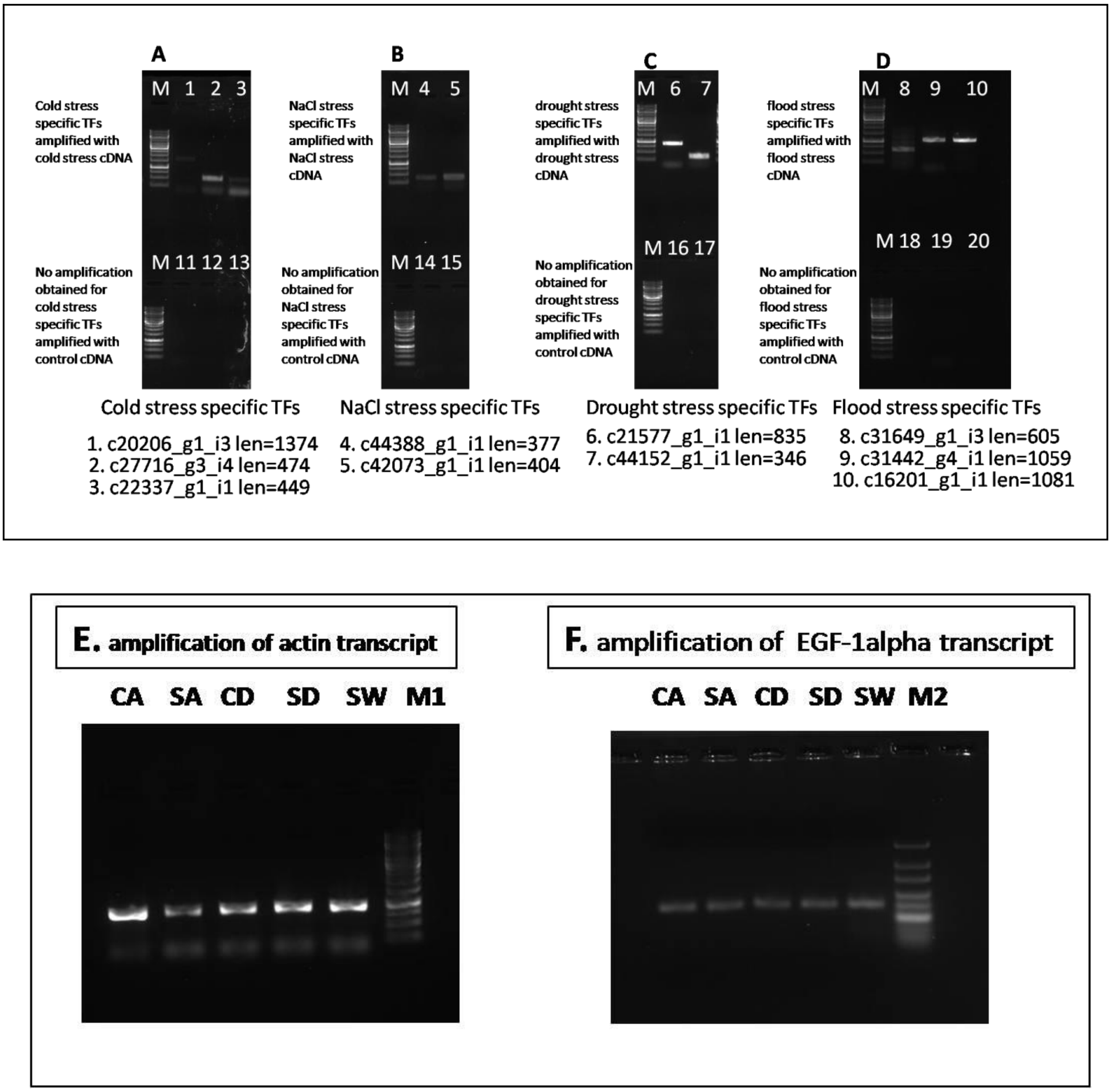

Figure S8: Amplification of stress specific transcription factors:
A 1,2,3: cold stress specific TFs (transcription factors) (1. c20206_g1_i3 length=1374bp, 2. c27716_g3_i4 length=474bp, 3. c22337_g1_i1 length=449bp) amplification with cold stress cDNA. **A 11, 12, 13**: no amplification obtained for cold stress specific TFs amplified with control cDNA. **B 4, 5**: NaCl stress specific TFs (4. c44388_g1_i1 length=377bp, 5. c42073_g1_i1 length=404bp) amplification with NaCl stress cDNA. **B 14, 15**: no amplification obtained for NaCl stress specific TFs amplified with control cDNA. **C 6, 7**: Drought stress specific TFs (6. c21577_g1_i1 length=835bp, 7. c44152_g1_i1 length=346bp) amplification with drought stress cDNA. **C16, 17**: no amplification obtained for drought stress specific TFs amplified with control cDNA. **D 8, 9, 10**: flood stress specific TFs (8. c31649_g1_i3 length=605bp, 9. c31442_g4_i1 length=1059bp, 10. c16201_g1_i1 length=1081bp) amplification with flood stress cDNA. **D 18, 19, 20**: no amplification obtained for flood stress specific TFs amplified with control cDNA. **M: 1 kb ladder. (Original Agarose gel picture is given below) E.** actin transcript (700bp) amplification**, F.** Elongation factor 1 alpha transcript (71bp) amplification. CA:control, SA:salt stress, CD:cold stress, SD: drought stress, SW: water-logging stress, M1: 1kb ladder, M2: ultra low range DNA ladder (Liu et al., 2015).

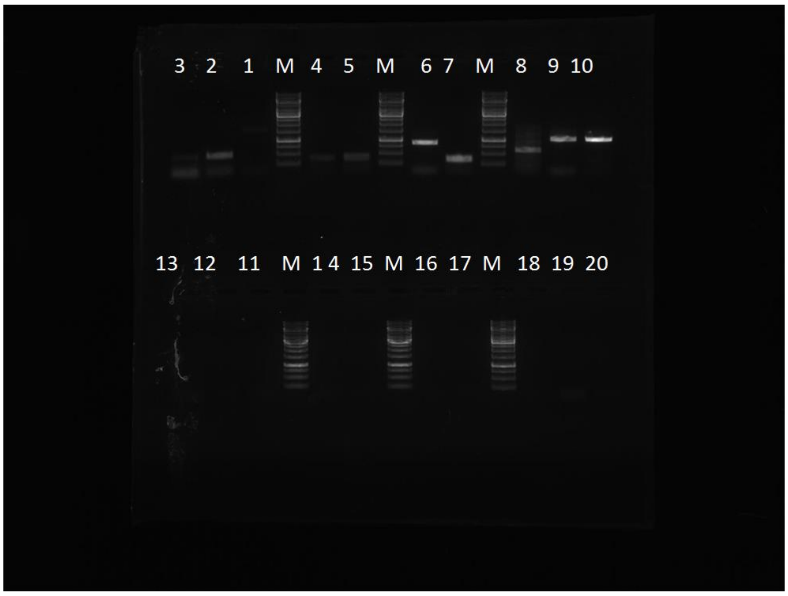

Transcritome Data Analysis Plan

Objective of the project:

i) De-novo Transcriptome Analysis

ii) Annotation of assembled transcripts

iii) Pathway Analysis

iv) Differential Gene Expression

Sample name: CO, CD, SA, SD, SW.

Sequencing platforms: Illumina Next*Seq500*

Data analysis tools: FastQC[1] , Trinity[2] , ncbi-BLAST-2.2.29+[3], CD-HIT[4], KAAS[5], DESeq[6]

Module1: Raw data processing and QC

1. The Illumina NextSeq paired end raw reads were quality checked using FastQC[1].

2. Illumina raw reads were processed by in-house script for adapters and low quality bases trimming towards 3'-end.

Module2: De-novo Assembly of Sequenced Transcriptome Data

De-novo assembly of Illumina NextSeq data was performed using Trinity[2] for default k-mers i.e 25.

Module 3: Transcripts Annotation

Transcripts were annotated using NCBI BLAST 2.2.29[3] with the proteins of Viridiplantae taken from Uniprot database. For annotation we have considered transcripts having length >=300 bp, followed by Clustering of these transcripts with 95% identity using CD-HIT[4] which resulted into COG's.

Module 4: Pathways Analysis

Pathway Analysis was done by using KAAS[5] Server. Arabidopsis thaliana (thale cress) and Solanum lycopersicum (tomato) were used as reference.

Workflow of Denovo Transcriptome Analysis

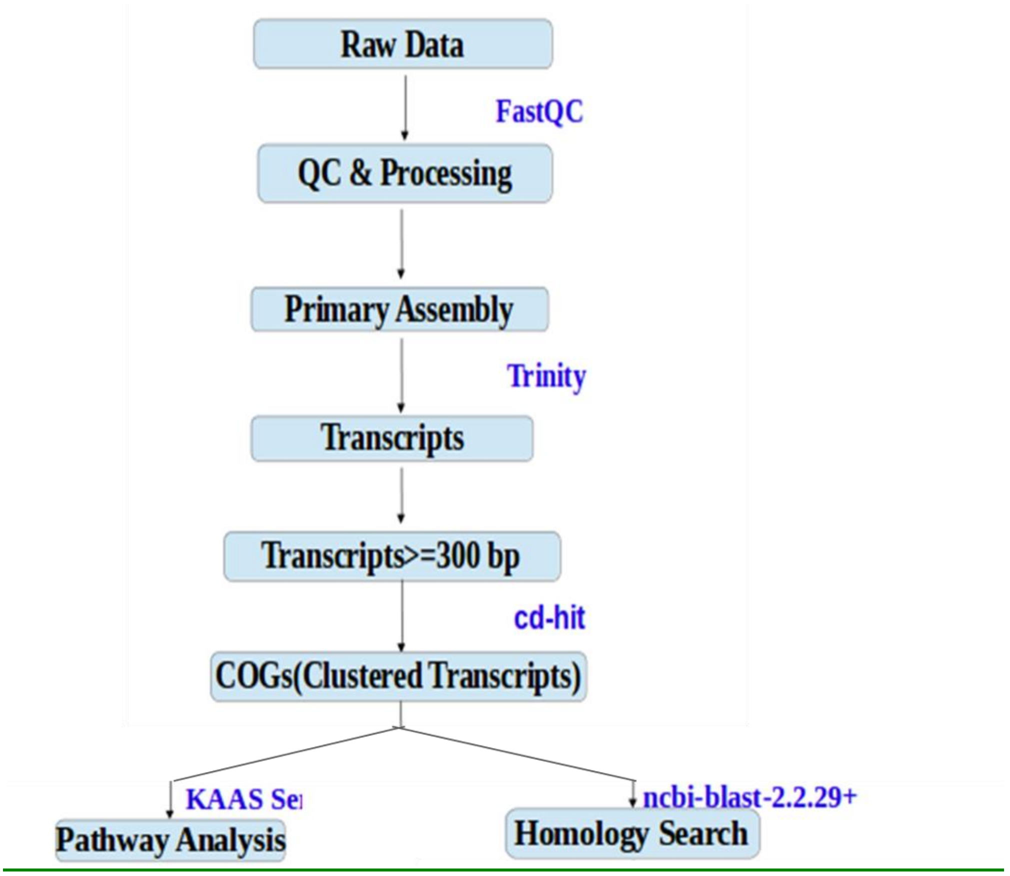

Module 5 : Differential Gene Expression Analysis

DGE

Sample name:CA, CD, SA, SD and SW

Sequencing platforms: Illumina Next*Seq500*

Data analysis tools: DESeq[6]

DESeq[6] was used for DGE, process of analysis is explained below:

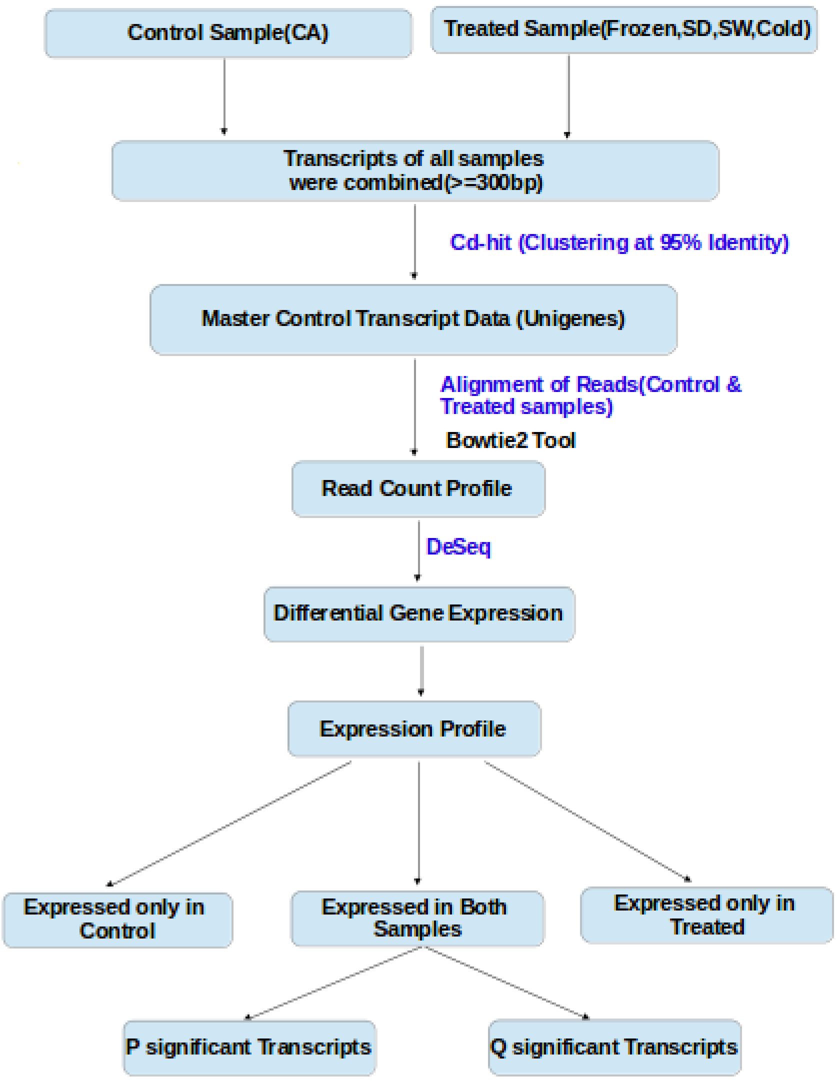

Analysis Tools Used

1. FastQC: http://www.bioinformatiSample1.babraham.ac.uk/projects/fastqc/

2. Trinity: Tool used for Denovo transcriptome assembly.

Citations: Grabherr MG, Haas BJ, Yassour M, Levin JZ, Thompson DA, Amit I, Adiconis X, Fan L, Raychowdhury R, Zeng Q, Chen Z, Mauceli E, Hacohen N, Gnirke A, Rhind N, di Palma F, Birren BW, Nusbaum C, Lindblad-Toh K, Friedman N, Regev A. Full-length transcriptome assembly from RNA-seq data without a reference genome.Nat Biotechnol. 2011 May 15;29(7):644-52. doi: 10.1038/nbt.1883.PubMed PMID: 21572440.

3. ncbi-BLAST-2.2.29+: Tool used for homology search.

Citation: Altschul, S; Gish, W; Miller, W; Myers, E; Lipman, D (October 1990). "Basic local alignment search tool". Journal of Molecular Biology 215 (3): 403–410. doi:10.1016/S0022-2836(05)80360-2. PMID 2231712.

4. CD-HIT: a fast program for clustering and comparing large sets of protein or nucleotide sequences.

Citation: Weizhong Li & Adam Godzik Bioinformatics, (2006) 22:1658-9

5. KAAS: Web server used for pathway analysis.

Citation: Moriya, Y., Itoh, M., Okuda, S., Yoshizawa, A., and Kanehisa, M.; KAAS: an automatic genome annotation and pathway reconstruction server. Nucleic Acids Res. 35, W182-W185 (2007)

6. DeSeq software (http://www-huber.embl.de/users/anders/DESeq/)

Citation: Anders, S. & Huber, W. Differential expression analysis for sequence count data. Genome Biol. 11(10), R106 (2010).

Databases used for the Analysis

1. Uniprot database (http://www.uniprot.org/)
